# Efficacy and cost-effectiveness of therapist-guided internet-delivered behaviour therapy for children and adolescents with Tourette syndrome: study protocol for a single-blind randomised controlled trial

**DOI:** 10.1186/s13063-021-05592-z

**Published:** 2021-09-30

**Authors:** Per Andrén, Lorena Fernández de la Cruz, Kayoko Isomura, Fabian Lenhard, Charlotte L. Hall, E. Bethan Davies, Tara Murphy, Chris Hollis, Filipa Sampaio, Inna Feldman, Matteo Bottai, Eva Serlachius, Erik Andersson, David Mataix-Cols

**Affiliations:** 1 Centre for Psychiatry Research, Department of Clinical Neuroscience, Karolinska Institutet, Gävlegatan 22, 113 30 Stockholm, Sweden; 2 Stockholm Health Care Services, Region Stockholm, Stockholm, Sweden; 3 Institute of Mental Health, Mental Health & Clinical Neurosciences, University of Nottingham, Nottingham, UK; 4 NIHR MindTech MedTech Co-operative, Institute of Mental Health, School of Medicine, Mental Health & Clinical Neurosciences, University of Nottingham, Innovation Park, Triumph Road, Nottingham, UK; 5 UCL Great Ormond Street Institute of Child Health (ICH), 30 Guilford Street, London, WC1N 1EH UK; 6 Psychological and Mental Health Services, Great Ormond Street Hospital for Children NHS Foundation Trust, Great Ormond Street, London, UK; 7 NIHR Nottingham Biomedical Research Centre, Institute of Mental Health, Division of Psychiatry and Applied Psychology, University of Nottingham, Innovation Park, Triumph Road, Nottingham, UK; 8 Department of Public Health and Caring Sciences, Uppsala University, Uppsala, Sweden; 9 Unit of Biostatistics, Institute of Environmental Medicine, Karolinska Institutet, Stockholm, Sweden

**Keywords:** Tourette syndrome, Tic disorders, Tics, Behaviour therapy, Exposure with response prevention, Internet-based interventions, Self-help

## Abstract

**Background:**

Treatment guidelines recommend behaviour therapy (BT) for patients with Tourette syndrome (TS) and chronic tic disorder (CTD). However, BT is rarely accessible due to limited availability of trained therapists and long travel distances to specialist clinics. Internet-delivered BT has the potential of overcoming these barriers through remote delivery of treatment with minimal therapist support. In the current protocol, we outline the design and methods of a randomised controlled trial (RCT) evaluating an internet-delivered BT programme referred to as BIP TIC. The trial’s primary objective is to determine the clinical efficacy of BIP TIC for reducing tic severity in young people with TS/CTD, compared with an active control intervention. Secondary objectives are to investigate the 12-month durability of the treatment effects and to perform a health economic evaluation of the intervention.

**Methods:**

In this single-blind superiority RCT, 220 participants (9–17 years) with TS/CTD throughout Sweden will be randomised to 10–12 weeks of either therapist-supported internet-delivered BT based on exposure with response prevention (*BIP TIC*) or therapist-supported internet-delivered education. Data will be collected at baseline, 3 and 5 weeks into the treatment, at post-treatment, and 3, 6, and 12 months post-treatment. The primary endpoint is the 3-month follow-up. The primary outcome is tic severity as measured by the Yale Global Tic Severity Scale – Total Tic Severity Score. Treatment response is operationalised as scores of “Very much improved” or “Much improved” on the Clinical Global Impression – Improvement scale, administered at the primary endpoint. Outcome assessors will be blind to treatment condition at all assessment points. A health economic evaluation of BIP TIC will be performed, both in the short term (primary endpoint) and the long term (12-month follow-up). There are no planned interim analyses.

**Discussion:**

Participant recruitment started on 26 April 2019 and finished on 9 April 2021. The total number of included participants was 221. The final participant is expected to reach the primary endpoint in September 2021 and the 12-month follow-up in June 2022. Data analysis for the primary objective will commence after the last participant reaches the primary endpoint.

**Trial registration:**

ClinicalTrials.gov NCT03916055. Registered on 16 April 2019.

**Supplementary Information:**

The online version contains supplementary material available at 10.1186/s13063-021-05592-z.

## Background

Tourette syndrome (TS) and chronic tic disorder (CTD) are childhood-onset neurodevelopmental disorders characterised by the presence of motor and/or vocal tics lasting longer than 1 year [1]. TS/CTD are associated with substantially impaired quality of life, academic performance, social adjustment, and emotional well-being [2, 3]. For a majority of individuals, the tics co-exist with a range of neurodevelopmental and psychiatric conditions, such as attention deficit/hyperactivity disorder (ADHD) or obsessive-compulsive disorder (OCD) [4].

Both European and American treatment guidelines recommend behaviour therapy (BT) as the first-line intervention for patients with TS/CTD [5, 6]. Among several modalities of BT, there is most evidence for the efficacy of *Comprehensive Behavioural Intervention for Tics* (CBIT) and its primary component *habit reversal training* (HRT) [7, 8]. Additionally, there is also support for the efficacy of *exposure with response prevention* (ERP) [9]. However, surveys have shown that BT is rarely available to patients with TS/CTD [4]. Reported reasons include a lack of information about TS/CTD among service users and providers, limited availability of trained therapists, and long travel distances to specialist treatment providers [10]. Two pilot studies have demonstrated that it is feasible to deliver BT for children and adolescents with TS/CTD in real time via videoconferencing software [11, 12]. This treatment format reduces the need for travel to the clinic but it still requires the same amount of therapist time as regular face-to-face BT.

A treatment format with the potential of overcoming both the long travel distances and the shortage of trained therapists is internet-delivered BT (IBT). In IBT, the participant logs into a secure online platform where the treatment is presented as a series of self-help texts, illustrations, and audio-visual materials, accompanied by homework assignments. During the treatment, a therapist (not necessarily an expert) provides guidance and gives feedback through text messages in a built-in messaging system [13]. Further, IBT only requires a fraction of the therapist time associated with regular BT. Evidence is growing to support the efficacy and cost-effectiveness of IBT for a wide range of mental and functional disorders in both children and adults [14–16].

The Child Internet Project (BIP in its Swedish acronym, *Barninternetprojektet*) is an IBT platform specifically designed for young people and their parents. Several trials using this platform have demonstrated that IBT is acceptable, efficacious, and cost-effective for children and adolescents with anxiety disorders [17–19], OCD [20–22], and functional gastrointestinal disorders [23, 24]. Given the limited availability of BT for TS/CTD and the success of previous BIP randomised controlled trials (RCTs), our team developed a first version of an IBT programme for TS/CTD, referred to as BIP TIC, in 2016. We subsequently evaluated the feasibility of two different versions of BIP TIC (based on HRT and ERP techniques, respectively) in a pilot RCT [25]. The results showed that both HRT and ERP could be delivered online with high adherence and satisfaction, while only requiring minimal therapist time (about 25 min per participant and week). However, only the ERP version of BIP TIC was found to significantly reduce tic severity, suggesting that ERP may be more easily adapted to an online format [23].

Before BIP TIC ERP (henceforth BIP TIC for simplicity) can be recommended for implementation in regular healthcare, rigorously designed RCTs evaluating its efficacy and cost-effectiveness are needed. Hence, BIP TIC is currently being evaluated in parallel in two large-scale superiority RCTs. The first RCT, called the *Online Remote Behavioural Intervention for Tics* (ORBIT) study, is based at two separate sites in England. Full details of the ORBIT study can be found elsewhere [26, 27]. The second RCT, called the BIP TIC RCT, is being conducted in Sweden and is described in the present study protocol.

The primary objective of the BIP TIC RCT is to determine the clinical efficacy of BIP TIC for reducing tic severity in children and adolescents with TS or CTD, compared with an active control intervention. Secondary objectives are to establish the 12-month durability of the treatment effects, and to conduct a health economic evaluation of the intervention, both in the short term (primary endpoint) and the long term (12-month follow-up).

## Methods

### Study design and setting

**Fig. 1:**
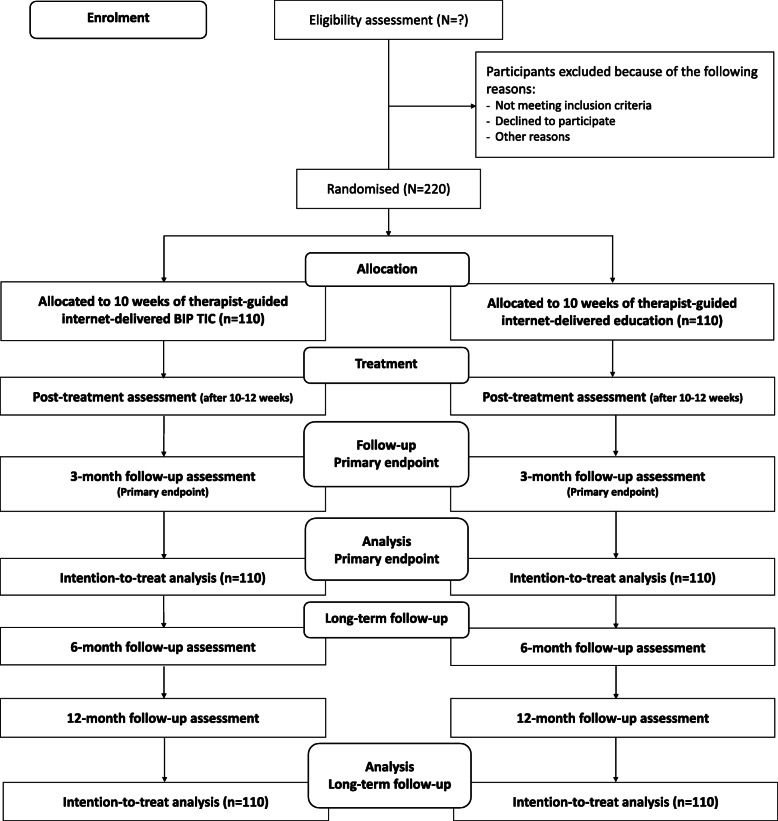
CONSORT 2010 flow diagram

The study is a single-blind, parallel group, randomised controlled superiority trial, comprising a 10- to 12-week intervention with a 12-month follow-up period. Participants will be randomised to either therapist-supported internet-delivered BT (ERP) for TS/CTD (henceforth referred to as *BIP TIC*) or therapist-supported internet-delivered education (henceforth referred to as the *comparator*). Assessment points comprise baseline, 3 weeks into the treatment, 5 weeks into the treatment, directly after the end of treatment (post-treatment), and follow-ups 3, 6, and 12 months post-treatment. The primary endpoint is the 3-month follow-up. During this phase, participants are encouraged not to start alternative treatments or change TS/CTD medication (compared to baseline). During the follow-up phase (6- and 12-month follow-ups post-treatment), participants may use alternative treatments or change their TS/CTD medication, in accordance with standard practice recommended by their treating clinician. A *Consolidated Standards of Reporting Trials* (CONSORT) 2010 flow diagram [28] of the study design is shown in Fig. 1. The study will be carried out at a single site, the Child and Adolescent Psychiatry Research Center, a research clinic within the Child and Adolescent Mental Health Services in Stockholm, Sweden. The study was prospectively registered with ClinicalTrials.gov (NCT03916055) on 16 April 2019 before inclusion of the first participant.

As previously mentioned, the study runs in parallel to the ORBIT trial [26]. The interventions are identical but there are some differences in the design (primary endpoint), follow-up schedule, inclusion and exclusion criteria, and outcome measures across the two trials (for an overview of the similarities and differences between the two trials, please see Table 1).

**Table 1:** Similarities and differences between the current trial (BIP TIC RCT) and the parallel Online Remote Behavioural Intervention for Tics (ORBIT) trial

Objectives	Both studies have identical main objectives (efficacy, durability, and cost-effectiveness). The ORBIT study further includes objectives on optimising the design and delivery of BIP TIC, undertaking an internal pilot, and conducting a process evaluation.
Study design and setting	Both studies are single-blind, parallel group, randomised controlled superiority trials, comprising two 10- to 12-week interventions. The primary endpoint of the BIP TIC RCT is 3 months after the end of treatment, whereas the primary endpoint of the ORBIT study is circa post-treatment (referred to as 3 months post-randomisation in the ORBIT protocol). The BIP TIC RCT includes assessment points at post-treatment, 3FU (primary endpoint), 6FU, and 12FU, while the ORBIT study includes assessment points at circa post-treatment (primary endpoint), 3FU, 9FU, and 15FU. Both studies maintain per protocol parallel group follow-up to circa 3 months post-treatment. After this point, participants in both trials may use alternative treatments for their tics. Both studies recruit nationally but the BIP TIC RCT is run from a single site (Stockholm), whereas the ORBIT study has two research sites (Nottingham and London).
Participants	Both studies recruit children and adolescents (9-17 years) with TS or CTD. There are some slight differences in the eligibility criteria, the primary being that the ORBIT study does not exclude participants with autism spectrum disorder or organic brain disorder.
Randomisation	Both studies randomise participants at a 1:1 ratio using block randomisation with varying block sizes. The ORBIT study further uses stratification by study site.
Interventions	Both studies evaluate the same two interventions (BIP TIC and the comparator), delivered through the same IBT platform (BIP). All chapters share the same overall content and are presented in the same order. Due to translation (from Swedish to English and back) and slight cultural adaptations, the exact content (e.g. wording, illustrations, and video scripts) may differ somewhat between the two studies. The key homework assignments are identical in both studies.
Outcome measures	Both studies share the same primary outcome measure (tic severity measured by the YGTSS-TTSS), and the same definition of treatment response (“Very much improved” or “Much improved” on the CGI-I). Several secondary measures such as the YGTSS Impairment, PTQ, C&A-GTS-QOL, and CGAS are identical, while other secondary measures differ between the studies. Cost measures differ across the two trials.
Blinding	Both studies use assessors who are blind to treatment allocation at all assessment points. Both studies take extensive measures to preserve blindness integrity. Statistical analyses are performed blindly.
Power analysis	Both studies aim to recruit 220 participants. The power calculations were performed using median-based methods (BIP TIC RCT) vs mean-based methods (ORBIT).
Statistical analyses	The statistical analyses of the primary outcome will be performed using a linear quantile mixed model, supplemented by a linear mixed model (BIP TIC RCT) vs linear regression (ORBIT).
Health economic evaluation	Both trials will perform a cost-effectiveness analysis (disorder-specific) and a cost-utility analysis (generic analysis with generic units [QALYs]). The outcomes for the disorder-specific analysis are the CGI-I-derived responder rate (BIP TIC RCT) and point change in YGTSS (ORBIT). In the BIP TIC RCT, QALYs are estimated by mapping the KIDSCREEN-10 onto CHU9D utilities, while ORBIT uses CHU9D directly. Data on healthcare and societal resource use are collected through the TiC-P (BIP TIC RCT) and the CSRI and CA-SUS (ORBIT).

Abbreviations: *3FU-15FU* assessment points 3–15 months post-treatment, *BIP* Barninternetprojektet (Swedish for “The Child Internet Project”), *BIP TIC* therapist-guided internet-delivered behaviour therapy (exposure with response prevention) for children and adolescents with Tourette syndrome or chronic tic disorder, *C&A-GTS-QOL* Child and Adolescent Gilles de la Tourette Syndrome–Quality of life scale, *CA-SUS* Child and Adolescent Service Use Schedule, *CGAS* Children’s Global Assessment Scale, *CGI-I* Clinical Global Impression – Improvement scale, *CHU9D* Child Health Utility 9 Dimensions, *comparator* therapist-guided internet-delivered education for children and adolescents with Tourette syndrome or chronic tic disorder, *CSRI* Client Service Receipt Inventory, *IBT* internet-delivered behaviour therapy, *ORBIT* Online Remote Behavioural Intervention for Tics, *post-treatment* assessment point directly after the end of treatment, *PTQ* Parent Tic Questionnaire, *TiC-P* Trimbos/iMTA questionnaire for costs associated with psychiatric illness, *YGTSS* Yale Global Tic Severity Scale, *QALY* quality-adjusted life year, *YGTSS-TTSS* Yale Global Tic Severity Scale – Total Tic Severity Score

### Participants

#### Eligibility criteria

The inclusion criteria for participation are as follows: (1) aged 9 to 17 years; (2) a diagnosis of *Tourette’s disorder* (i.e. TS) or *persistent (chronic) motor or vocal tic disorder* (i.e. CTD), based on the 5th edition of the Diagnostic and statistical manual of mental disorders (DSM-5) (1); (3) a *Yale Global Tic Severity Scale* (YGTSS) *Total Tic Severity Score* (TTSS) of > 15 (or > 10 if only motor or vocal tics, but not both, have been present during the last week) [29]; (4) at least one available parent/caregiver (henceforth referred to as parent) to support the child/adolescent (henceforth referred to as child) throughout the treatment; and (5) regular access to a computer connected to the internet, with the ability to receive emails, as well as a mobile phone to receive text messages (one of each per family is enough).

The exclusion criteria are as follows: (1) at least 8 previous sessions of BT for tics with a qualified therapist within the 12 months prior to assessment; (2) simultaneous psychological treatment for TS or CTD; (3) initiation or adjustment of any psychotropic medication for TS or CTD within the 8 weeks prior to assessment; (4) a diagnosis of organic brain disorder, intellectual disability, autism spectrum disorder, psychosis, bipolar disorder, anorexia nervosa, or alcohol/substance dependence; (5) immediate risk to self or others requiring urgent medical attention, such as suicidality or self-injurious tics; (6) the child or parent are not able to read and communicate in Swedish; or (7) a close relative (e.g. sibling or cousin) is already enrolled in the trial (to remove the risk of them being randomised into two different groups).

#### Recruitment

The trial is open for participants from every region of Sweden. Participants can either self-refer to the trial through the study website or be referred by a healthcare professional to our specialist TS/CTD clinic. The study will be advertised to health care services, patient organisations, and directly to the public via the study website and social media. We will also publish paid advertisements in print and digital media.

Following referral, the participant will be assigned a screening ID and the parent will be contacted via telephone by a member of the research team to provide information about the trial and perform a preliminary eligibility screening. If they are interested in participating and potentially eligible, the participant will be booked for an inclusion assessment, face-to-face at the clinic or via videoconference software (depending on the family’s preference and geographical location [no travel expenses are reimbursed]). Prior to the inclusion assessment, the family will be sent information via regular mail, including the informed consent form, an age-appropriate participant information sheet, and login information to complete child- and parent-reported questionnaires online (see “Outcome measures” section). The rationale for collecting these baseline data before the inclusion assessment is to improve the clinical assessment and to promote participant safety by screening for risk factors (such as depressive symptoms).

The inclusion assessment will be conducted by a clinical psychologist under supervision of a clinical expert (PA) with both the child and at least one of the parents present (face-to-face or via video conference). The assessment includes verifying the diagnosis of TS or CTD according to DSM-5 criteria [1], the administration of the YGTSS (symptom checklist and symptom ratings) [29] to assess tic severity and tic-related impairment, the Mini-International Neuropsychiatric Interview for children and adolescents (MINI-KID) [30] and supplementary modules for the assessment of obsessive-compulsive and related disorders to assess comorbidities, and the collection of socio-demographic information. Right after this assessment, more information about the trial will be provided, a final verification of the eligibility criteria will be performed, and the informed consent forms will be signed by the child and both parents. If the assessor is uncertain about the eligibility of a potential participant, the principal investigator (DM-C) will have the final decision. Excluded participants may be eligible for future re-screening if the eligibility criteria are then met (e.g. when 8 weeks on stable medication for TS/CTD have passed). Once the informed consent forms have been signed, the participant will be assigned a study ID, be randomised, and start treatment within 1 week from randomisation. Excluded participants who still require clinical attention will be referred to other suitable services, whenever possible. Screening IDs and reasons for exclusion will be stored for reporting purposes.

### Randomisation and allocation concealment

Participants will be randomised to either BIP TIC or the comparator at a 1:1 ratio. Randomisation will be conducted by several assigned researchers (according to a task delegation list) using an online randomisation service (Randomize.net) [31], set up and monitored by the Karolinska Trial Alliance (KTA), which is an independent clinical trials unit [32]. Randomly varying block sizes (inaccessible to the research team) will be generated using a computer random number generator. Several assigned researchers will be responsible for enrolling participants and assigning participants to therapists. Participants will be informed that they will be allocated to one out of two behavioural interventions for TS/CTD, without providing specific detail about each of the interventions’ content.

### Interventions

#### Treatment format and therapist support

Both interventions will be delivered via the BIP platform and will include age-appropriate self-help texts, illustrations, instructional videos, worksheets, exercises, and homework assignments. Supplementary file 1 shows screenshots of various functions of the BIP platform and the two interventions.

During the treatment, both the child and the parent will have individual asynchronous access to a designated therapist who will support the families through the BIP platform, primarily through text messages (emails or comments on worksheets). If clinically needed, the child/parent can also contact their therapist via telephone, but this will generally be kept to a minimum. The therapist’s role is to provide feedback, answer questions, and encourage uptake and adherence to the interventions. The child/parent can write to their therapist at any time, while the therapist logs in to provide guidance at least every 48 h (on workdays). There are no specified limits to the therapist support. However, based on our pilot trial [25], we expect the average therapist time to be around 25 min per participant and week. All therapist time is logged, either automatically (text communication within the platform) or manually (phone calls). The therapists are clinical psychologists or trainee psychologists (under supervision of a senior clinical psychologist) trained in BT. All therapists will receive training (before the trial starts) and supervision (throughout the trial) by a clinical expert (PA). For a complete description of the therapist training procedures, see the full study protocol in Supplementary file 2.

In addition to the therapist, the child will also receive support from their parents during the treatment. The designated parent will have their own separate login to the BIP platform and access to their own modules. Typically, only one parent per family will have this supporting role but it is possible to have two parallel parent logins if the parents do not co-habit. The parent’s role is to support the child throughout the treatment, which includes a variety of tasks such as helping the child to log in, assisting on treatment specific exercises, and using parental coping strategies.

Each intervention consists of 10 chapters (modules) for both the child and the parent/s, delivered over 10 weeks. In certain circumstances (e.g. illness or holidays), participants can pause their therapist support for 1 or 2 weeks, which extends the treatment length to a maximum of 12 weeks (of which 10 weeks include therapist support). The first three chapters can be completed at the family’s own pace (e.g. they can be completed within a few days), while the remaining seven chapters are intended to be completed at a pace of one per week. *Treatment completion* is defined as the completion of the first four child chapters, which are designed to include the core elements of each intervention (which we hypothesise are sufficient to allow the participants to continue to use the treatment techniques autonomously in the future). After the 10 to 12 weeks of intervention, families will continue to have online access to all chapters (without therapist support) for 1 year (12-month follow-up).

Table 2 contains an overview of the chapter contents, and Supplementary file 1 shows screenshots, of the two interventions. For the duration of the trial, all participants are allowed to receive treatment as usual for their general health and psychiatric comorbidities other than TS/CTD (e.g. management of ADHD medication). Additionally, TS/CTD medication is allowed as long as the dose remains stable between baseline and the 3-month follow-up.

**Table 2:** Overview of the BIP TIC and comparator chapters for children and parents

Chapter	BIP TIC child	Comparator child	BIP TIC parent	Comparator parent
1	Information about the internet format and platform* Basic information about tics*		Information about the internet format and platform* Information about the parent role* Contingency management (token economy*)	
2	How tics can be bothersome* Premonitory urges* Make a list of current tics*		Common thoughts, feelings, and behaviours of parents How to not comment on the tics	How to praise the child during the treatment activities
3	Response prevention practice	“Become an expert in tics”, including information about the natural course of tics and famous people with tics	How to praise the child during the treatment activities	How to prompt (remind) the child to use treatment strategies
4	Add exposure to the response prevention	Information about common comorbid psychiatric conditions	How to prompt (remind) the child to use treatment strategies	Information about common comorbid psychiatric conditions
5	Continued exposure with response prevention practice	Engage in healthy habits, such as daily routines, exercise, good nutrition, and sleep hygiene	Functional assessment and interventions	Healthy habits for the child and the parent
6	Continued exposure with response prevention practice How to cope with tics in school How to cope with bullying	Continued engagement in healthy habits How to cope with tics in school How to cope with bullying	Troubleshooting the exposure with response prevention practice	How to cope with tics in school
7	Continued exposure with response prevention practice How to tell others about tics	Continued engagement in healthy habits How to tell others about tics	Continued practice of treatment strategies	Common thoughts and feelings of parents
8	Continued exposure with response prevention practice	Information about risk and protective factors for tics	Continued practice of treatment strategies	Information about risk and protective factors for tics
9	Continued exposure with response prevention practice	Information about research studies on tics The future for people with tics	Continued practice of treatment strategies	Parental self-care
10	Summary of chapters 1 to 9* Make a plan for continued practice in the future*		Summary of parental treatment strategies* Make a plan for continued parental support in the future*	

Note: Each parent chapter also includes the key information from the corresponding child chapter, so that the parent does not miss out on what the child is learning in their chapter

*Denotes that the same content appears in both BIP TIC and the comparator

Abbreviations: *BIP TIC* therapist-guided internet-delivered behaviour therapy (exposure with response prevention) for children and adolescents with Tourette syndrome or chronic tic disorder, *comparator* therapist-guided internet-delivered education for children and adolescents with Tourette syndrome or chronic tic disorder

#### BIP TIC

BIP TIC is primarily based on an existing evidence-based ERP manual for TS/CTD [9, 33]. Additional components (e.g. functional assessment and interventions) are mainly based on the CBIT manual [7, 34]. The primary focus of BIP TIC is ERP. In ERP, the child practices to suppress tics (referred to as *response prevention*), while gradually provoking their *premonitory urges* (i.e. unpleasant sensations preceding tic occurrence) to make tic suppression more challenging (referred to as *exposure*). The aim of ERP is to increase the child’s ability to voluntarily suppress tics in various situations for prolonged periods of time. To help motivate the child to engage in ERP practice, the child modules include a built-in stopwatch which generates high-score lists of tic suppression times. The parent modules contain the key information from the child modules, as well as parent-specific information on how to support the child’s ERP practice and other treatment activities.

#### Comparator

The active *comparator* was designed to resemble the comparator used in previous large RCTs of face-to-face BT for TS/CTD [7, 8], thus ensuring maximum comparability with those trials. It primarily consists of education about TS/CTD and common comorbid conditions, as well as various behavioural exercises (e.g. sharing knowledge about TS/CTD with family/friends and engaging in healthy everyday habits, such as improved sleep hygiene and regular physical exercise). Development of expertise in TS/CTD is emphasised throughout the intervention. Similarly to BIP TIC, the comparator parent modules contain the key information from the child modules, as well as parent-specific information on how to support the child’s various treatment activities.

The comparator is designed to match BIP TIC in all aspects, except for the chapter content (e.g. online format, platform/appearance, number of chapters, approximately the same chapter length, format of therapist support, similar use of homework assignments). Some chapter content is common to both interventions, including basic education about TS/CTD, the possibility to use contingency management (token economy) to enhance treatment activity, keeping a list of current tics, information on how to cope with tics in the school environment, how to tell other people about tics, and several elements of the parental coping strategies. Information on common comorbid conditions, healthy habits, and risk and protective factors is unique to the comparator, while information on ERP and functional assessment and interventions is only included in BIP TIC. The comparator ensures that the participants receive an intervention over and above what would be typically received in standard care, and aims to control for online access to basic information about TS/CTD, therapist support, and homework assignments.

### Outcome measures

#### Primary outcome measure

**Fig. 2:**
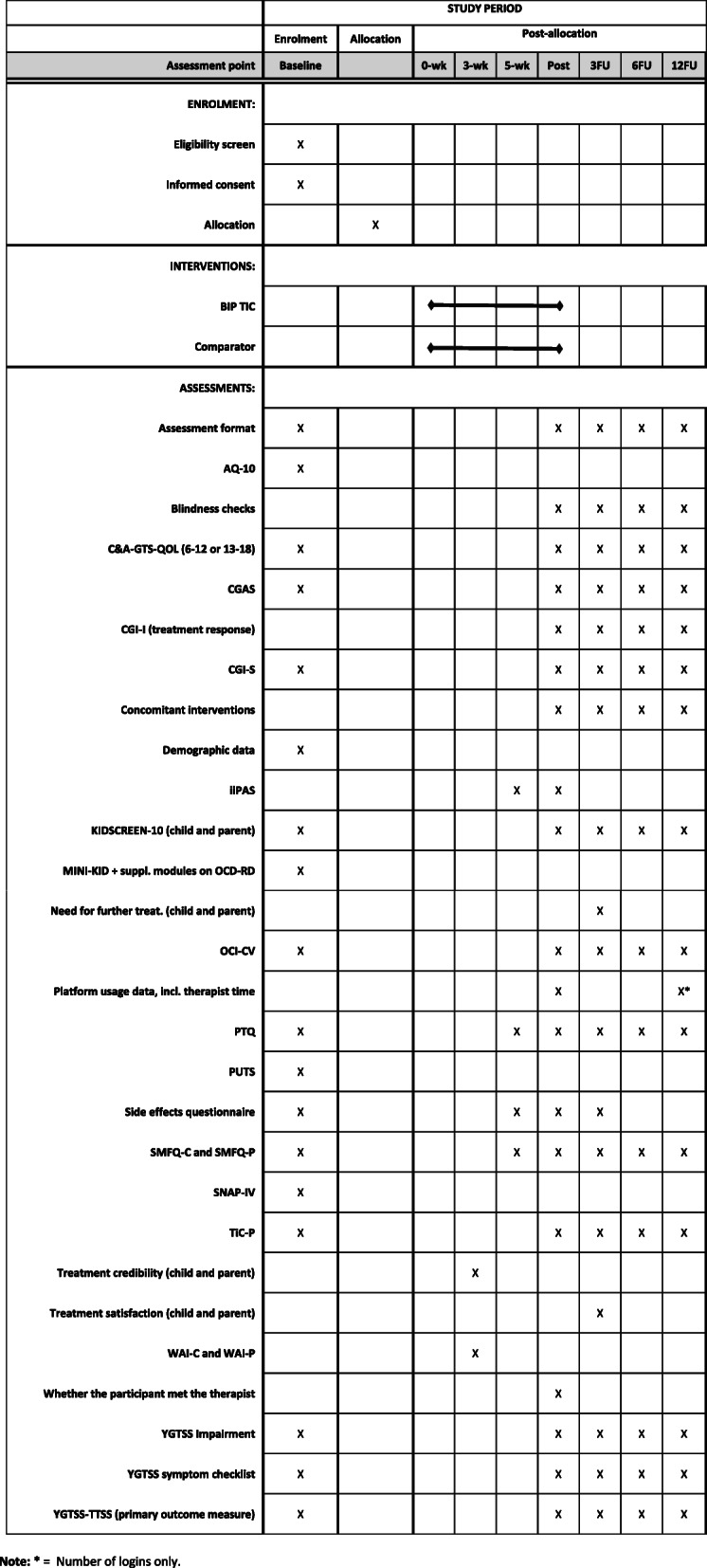
SPIRIT 2013 schedule of enrolment, interventions, and assessmentsAbbreviations: 0-wk = 0 weeks in to treatment, the equivalent of the treatment start; 3FU-12FU = assessment points 3-12 months after the end of treatment; 3-wk-5wk = assessment points 3-5 weeks in to treatment; AQ-10 = Autism Spectrum Quotient, 10-item version; BIP TIC = therapist-guided internet-delivered behaviour therapy (exposure with response prevention) for children and adolescents with Tourette syndrome or chronic tic disorder; C&A-GTS-QOL = Child and Adolescent Gilles de la Tourette Syndrome–Quality of life scale; CGAS = Children’s Global Assessment Scale; CGI-I = Clinical Global Impression – Improvement scale; CGI-S = Clinical Global Impression – Severity scale; comparator = therapist-guided internet-delivered education for children and adolescents with Tourette syndrome or chronic tic disorder; iiPAS = Internet Intervention Patient Adherence Scale; MINI-KID = Mini-International Neuropsychiatric Interview for children and adolescents; OCD-RD =obsessive-compulsive and related disorders; OCI-CV = Obsessive-Compulsive Inventory – Child version; PTQ = Parent Tic Questionnaire; post = post-treatment, assessment point directly after the end of treatment; PUTS = Premonitory Urge for Tics Scale; SMFQ-C = Short Mood and Feelings Questionnaire – Child version; SMFQ-P = Short Mood and Feelings Questionnaire – Parent version; SNAP-IV = Swanson, Nolan, and Pelham rating scale; TiC-P = Trimbos/iMTA questionnaire for costs associated with psychiatric illness; WAI-C = Working Alliance Inventory – Child version; WAI-P = Working Alliance Inventory – Parent version; YGTSS = Yale Global Tic Severity Scale; YGTSS-TTSS = Yale Global Tic Severity Scale – Total tic severity score

An overview of all outcome measures and assessment points is presented in Fig. 2. The primary outcome is tic severity as measured by the YGTSS-TTSS [29]. This clinician-rated semi-structured interview is the most commonly used outcome measure in the field [35], enabling comparisons with previous RCTs of TS/CTD. The YGTSS-TTSS is derived by adding the Motor tic severity score (5 items, score range 0–25) and the Vocal tic severity score (5 items; score range 0–25), resulting in an ordinal variable ranging from 0 to 50 points, with higher scores indicating higher tic severity. The YGTSS-TTSS will be administered at all assessment points (except week 3 and week 5), with the 3-month follow-up being the primary endpoint. The assessments will primarily be conducted face-to-face at the clinic or remotely via videoconference software, with telephone as an alternative option (e.g. if technical problems). The YGTSS-TTSS has sound psychometric properties [29, 36, 37].

Extensive steps will be taken to minimise measurement bias on the primary outcome. All assessors will be extensively trained by a clinical expert (PA; see the full study protocol (Supplementary file 2) for a complete description of the training procedures) and inter-rater reliability will be established. All YGTSS-TTSS assessments will be video recorded for monitoring, spot check, and inter-rater reliability purposes.

#### Secondary outcome measures

As with the YGTSS-TTSS, all clinician-rated measures are collected face-to-face, via videoconference or via telephone. All self- and parent-reported measures are remotely administered via an online service, which ensures automatic and complete entry of each measure into the trial database.

Concurrently with the administration of the YGTSS-TTSS, the YGTSS symptom checklist and the YGTSS Impairment will also be administered [29]. The YGTSS Impairment is a single-item clinician rating of distress and impairment associated with the presence of tics.

The Clinical Global Impression (CGI) scales will be used as secondary clinician-rated measures of TS/CTD symptom severity (CGI Severity; CGI-S) and global improvement (CGI Improvement; CGI-I) compared to baseline [38]. In line with previous trials of BT for TS/CTD [7, 8], treatment response will be operationalised as scores of “Very much improved” (1) or “Much improved” (2) on the CGI-I. The CGI is widely used in mental health trials [39].

Other secondary clinician-rated outcomes include general functioning measured with the Children’s Global Assessment Scale (CGAS) [40, 41], and participant adherence to the internet-delivered interventions measured with the Internet Intervention Patient Adherence Scale (iiPAS) [42].

The Parent Tic Questionnaire (PTQ) will be used as a parent-reported measure of tic severity [43]. Child-reported TS-specific quality of life will be measured by the Child and Adolescent Gilles de la Tourette Syndrome–Quality of Life (C&A-GTS-QOL) scale (two different versions for ages 6-12 and 13-18) [44]. Depressive symptoms will be measured by the Short Mood and Feelings Questionnaire (child and parent-reported versions; SMFQ-C and SMFQ-P, respectively) [45, 46]. OCD symptoms will be measured by the child-reported Obsessive-Compulsive Inventory – Child Version (OCI-CV) [47, 48]. The parent-reported Side Effects Questionnaire will be used to record side effects (adverse events) [49]. Since all participants in the study have tics, the wording of one item has been modified to refer to an increase in tic frequency, rather than tic prevalence. Additional questionnaires on treatment satisfaction (9 items) and need for further TS/CTD treatment after treatment completion (1 item) have been developed for the study and will be administered to both children and parents.

To compare treatment credibility between the two interventions, a questionnaire developed by the research team (3 items) will be administered to both children and parents 3 weeks into the treatment. The questionnaire asks about the treatment’s suitability for managing tics, expected amount of symptom improvement, and degree of motivation for treatment. Similarly, to assess differences between groups in therapist working alliance, the Working Alliance Inventory (child- and parent-reported versions; WAI-C and WAI-P, respectively) will be administered [50] 3 weeks into the treatment. The wording of the WAI-C has been slightly adjusted by the research team to better suit the youngest participants.

For the health economic analyses, the KIDSCREEN-10 will be administered to both children and parents to assess health-related quality of life of the child and estimate quality-adjusted life years (QALYs) [51, 52]. The parent-reported Trimbos/iMTA questionnaire for costs associated with psychiatric illness (TiC-P) [53] is frequently used in health economic studies and has been adapted for young people and parents for use in this trial to assess healthcare and societal resource use.

#### Additional measures

Additional measures will be collected exclusively at baseline to further describe the sample. These data may also be used for post hoc analyses investigating predictors and moderators of treatment response. The measures include clinician-administered and parent-reported demographic questionnaires, both specifically developed for the trial. Further, the Premonitory Urge for Tics Scale (PUTS) will be used as a child-reported measure of premonitory urges [54], the Swanson, Nolan, and Pelham rating scale (SNAP-IV) as a parent-reported measure of ADHD and oppositional defiance disorder symptoms [55, 56], and the Autism Spectrum Quotient (AQ-10) as a parent-reported measure of autistic symptoms [57].

Further, using interviews specifically developed for the trial, the assessors will collect data on concomitant interventions (e.g. medication or psychological interventions), assessment format (face-to-face, via videoconference software, or via telephone), and whether the participants met their therapist (face-to-face or videoconference) at any time prior to post-treatment. Platform usage data will be collected automatically in the BIP platform, including data on therapist time and data from the BIP TIC stopwatch (see the full study protocol (Supplementary file 2) for a complete description).

### Safety procedures

Adverse events will be monitored through the parent-reported Side Effects Questionnaire [49] (see Fig. 2 for assessment points). Additionally, a log will be kept for adverse events reported through participant contact with therapists, assessors, and other trial staff. High scores on certain questionnaires, such as the SMFQ, might also be indicators of adverse events. To regularly monitor suicide risk, we have added an additional item to the SMFQ-C (not included in the total score) asking about suicidal ideation during the last 2 weeks. High SMFQ-C and SMFQ-P scores, or high scores on the suicide item, will automatically raise a flag in the online monitoring system and directly notify members of the research team to contact the participant (via telephone) for an additional assessment. Twice weekly meetings (ward rounds) will be held within the research team where the participants’ treatment progress and potential adverse events are discussed. Adverse events are logged regardless of whether they are judged to be related to treatment. The relation between potential serious adverse events and treatment will be decided by the principal investigator, based on all available information. Serious adverse events will be considered as potentially treatment-related up to the 3-month follow-up, where the systematic reporting of adverse events will end. Using data from our pilot study [25], some adverse events are defined a priori as expected (see the full study protocol (Supplementary file 2) for a complete list). All other adverse events are defined as unexpected. The adverse events reporting will be monitored by the KTA. Appropriate action will be taken in the case of serious adverse events, such as ensuring that the participant will contact suitable health care services.

### Blinding

The principal investigator, outcome assessors, statistician, and health economists are blind to group allocation. Study participants are not blind but do not have a priori information about the content of each intervention (they are informed that they will be allocated to one of two behavioural treatments for TS/CTD). Participants are instructed not to reveal details about the treatment they received to the blind assessor. Blind assessors will record whether the participating families inadvertently reveal their treatment allocation. In addition, assessors will try to guess each participant’s allocation at each assessment point and motivate their choice (e.g. due to clinical improvement or random choice). If the treatment allocation is accidently revealed, a new blind assessor will watch an edited version of the video recording and will independently conduct the clinician-rated YGTSS, CGI-S, CGI-I, and CGAS ratings that will be used in the analyses. Subsequent assessments for that participant will then be conducted by a new blind assessor.

The primary data analysis will be conducted after the last participant has completed their 3-month follow-up assessment (primary endpoint), but assessors will remain blind to individual participants’ allocation for the full duration of the trial (i.e. until the 12-month follow-up). The trial coordinator, statistician, and health economists will be blind when performing the primary analysis through the use of dummy variables for participant ID and group allocation (see Supplementary file 2 for more information). The therapists will not be blind to treatment allocation, hence no emergency unblinding system is required for the trial.

### Patient and public involvement

Prior to the development of BIP TIC, we assembled a focus group in Stockholm, including five children with TS/CTD and their parents. From the focus group, we learnt that young people and their parents are enthusiastic about digital interventions for TS/CTD. They especially liked the idea of having access to help and information on their own device at their own chosen time, in addition to remote therapist support. This feedback informed the initial development of BIP TIC.

At the end of our pilot trial [25], we gathered extensive user experience data and conducted detailed qualitative interviews with the families. Participants evaluated the intervention as highly acceptable, safe, and helpful in reducing tics. Satisfaction ratings were high [25]. This feedback from young people and their parents, together with feedback from treating therapists and the input from our collaborators in England, has shaped BIP TIC and the comparator to the current versions that will be used in this RCT (see Supplementary file 2 for more information).

Relevant Swedish patient organisations (*Riksförbundet Attention* and *Svenska OCD-förbundet*) and healthcare providers have been informed about the trial.

### Power analysis

Because the primary outcome measure (YGTSS-TTSS) is integer-valued (ordinal) in nature, we estimated the power for the difference in *median* scores between the two treatment groups at the primary endpoint, using data from our pilot trial [25]. Specifically, we used a Wald test for the coefficient of the interaction term in a linear quantile random intercept model for the median of the outcome [58–60]. The model contained the intercept, the binary treatment indicator (BIP TIC, comparator), the numeric time variable (baseline, post-treatment, 3-month follow-up), and the treatment-by-time interaction term. We calculated the power under different samples sizes and differences in median outcome between the two treatment groups at the primary endpoint. For each combination of sample size and difference, we simulated 500 samples under a random intercept model with normal intercept and normal residual error. The regression parameters and variance components were obtained from our pilot data [25]. The regression coefficients were 28.56 for the intercept, and − 3.11 for time, while the standard deviation of the random intercept was 2.95, and that of the residual was 2.04. These calculations showed that, with 200 participants (100 in each arm), we would have 97% power to detect a statistically significant difference in medians of 3 points on the YGTSS-TTSS at the primary endpoint. Allowing for a 10% dropout rate, this trial will aim to recruit 220 participants.

### Statistical analyses

Statistical analyses will be conducted under guidance of the Biostatistics Core Facility [61] at Karolinska Institutet (clinical efficacy and 12-month durability) and the Department of Public Health and Caring Sciences at Uppsala University (health economics). The statistician, the health economists, the trial coordinator, and the principal investigator will have full, unrestricted access to the study data. See the appendix of Supplementary file 2 for the full statistical analysis plan.

The demographic information collected at baseline will be summarised and presented in a table, by randomisation arm. Categorical variables will be reported as counts and percentages. Continuous variables will be summarised as means, medians, and interquartile ranges. According to CONSORT recommendations, no statistical tests will be performed to assess baseline differences between study arms [62]. No interim analyses are planned.

#### Primary outcome analysis

The primary outcome analysis will be based on all available data up to this assessment point and conducted according to the intention-to-treat (ITT) principle. As the primary outcome (YGTSS-TTSS) is an integer-valued variable (ranging from 0 to 50), statistical modelling will focus on the median of the outcome rather than its mean. Specifically, all the randomised participants will be included to estimate a linear quantile mixed model [58–60], taking into account individual differences in pre-treatment symptomatic status and treatment response. The model will include fixed effects for time (the YGTSS-TTSS at baseline, post-treatment, and 3-month follow-up) and subject-specific effects as a random intercept factor to account for the variances between and within participants. Linear quantile mixed models use all available data, can account for correlation between repeated measurements on the same subject, can flexibly model time effects, and can handle missing data.

To enable comparisons with previous trials within the field, which traditionally have used regression models based on means rather than medians, we will also perform complementary analyses of the primary outcome measure at the primary endpoint using a linear mixed model (estimating a difference in means).

The estimated treatment effect will be reported with accompanying 95% confidence interval (CI) and *p* value. The magnitude of the treatment effects will be presented as between-group differences in median relative the interquartile range (for median comparisons) and as standardised between-group effect sizes (Cohen’s *d*; for mean comparisons) [63]. Throughout the trial, the alpha level of 0.05 will be used as the threshold for statistical significance.

### *Secondary outcomes analyses*

Secondary outcomes will be analysed using similar statistical methods as the primary outcome, that is, with linear quantile mixed models, and complemented with linear mixed models to facilitate comparison with previous trials. Dichotomous variables will be analysed using logistic regression. The results will be presented as estimates or odds ratios, as appropriate, for the regression coefficients, with their respective 95% confidence intervals and *p* values.

#### Long-term follow-up analyses

The long-term follow-up analyses will use linear quantile mixed models and logistic regression. In addition, to enable comparisons with previous trials in the field, we will also perform complementary linear mixed models (estimating a difference in means) for each of the linear quantile mixed models performed. Primarily, the regression models will include the 3-, 6-, and 12-month follow-up assessment points, and evaluate in a within-group analysis whether the potential short-term treatment effects demonstrated for BIP TIC are sustained at the 12-month follow-up. Additionally, we will also enter all available assessment points to a separate regression model to investigate whether there are significant between-group effects at the 12-month follow-up.

#### Health economic evaluation

A short-term health economic evaluation will compare BIP TIC and the comparator at the primary endpoint (3-month follow-up). Additionally, an equivalent long-term evaluation will be performed at the end of the follow-up period (12-month follow-up) using cumulative data collected up to that assessment point. The health economic evaluation will be performed from three different perspectives, with gradually increasing costs included for each perspective. The first perspective is the health organisation payer perspective, which includes direct treatment costs, of BIP TIC and the comparator, for the clinic. This comprises personnel costs such as therapist time, administration time, IT platform maintenance, and other overheads. The second perspective will additionally include other healthcare resource use outside the clinic for the children, such as costs for medical appointments (outside the trial) and medications. The third perspective will further comprise other societal costs, including productivity losses for the children related to absenteeism and presenteeism from school and leisure activities, and productivity losses for the parents due to absenteeism from work. The TiC-P [53] will be administered at each assessment point (except 3 and 5 weeks into the treatment) to collect information on frequencies of resource use for the children and absenteeism from work for the parents. Resource use costs will be estimated by multiplying frequencies by national Swedish tariffs and market prices. Total costs for each group will be aggregated over the trial period.

For each of the three perspectives above, we will conduct two types of analyses: (1) a cost-effectiveness analysis, using responder rate as the outcome [64]; and (2) a cost-utility analysis using QALYs as the outcome [64]. Health gains in terms of QALYs will be estimated by mapping KIDSCREEN-10 scores [51] onto Child Health Utility 9 Dimensions (CHU9D) utility weights [65]. Total QALY gains over the trial period will be estimated by using the area under the curve method [66]. Differences in QALYs and costs between both trial arms will be investigated using generalised linear models with suitable distributions [67].

To ascertain whether BIP TIC is cost-effective, relative to the comparator, incremental cost-effectiveness ratios expressed as cost per additional responder and cost per additional QALY will be presented. The uncertainty around the cost and effect estimates will be presented using a cost-effectiveness acceptability curve [64].

### Quality control

The trial will be conducted according to good clinical practice (GCP) principles. All trial staff will attend a course in GCP arranged by the KTA. Quality procedures, including documentation and randomisation, will be set up with the help of the KTA. GCP documents such as source data and task delegation lists will be established. The KTA will perform case-by-case monitoring of informed consent, eligibility criteria, data entry (i.e. 100% source data verification [SDV] of the primary outcome measure from baseline to the primary endpoint and 10% spot check SDV of all clinician-reported measures at all measure points), and adverse events. The trial coordinator (PA) will regularly monitor (spot check) the therapist-participant communication inside the BIP platform. Therapist drift (from the participants’ assigned allocations) will be addressed and recorded.

### Ethics and dissemination

The study is approved (full protocol versions 1.0, 2.0 and 2.3) by the Swedish Ethical Review Authority 2018/1788-31/2 (2019-01670; 2020-04836). We estimate that the study poses little risk to participants. All participants will be offered a thorough psychiatric assessment and access to a dedicated therapist and will be followed-up long term. No participant will be denied any current standard treatment between enrolment and the primary endpoint (other than face-to-face BT, which is rarely available). Medication for tics is permitted if stable. We predict that the comparator will be less effective than BIP TIC in reducing tics, but previous literature indicates that similar interventions may offer some therapeutic benefits (such as reducing tic-related impairment) [7]. Additionally, education on tics is included in several evidence-based protocols for BT [33, 34]. Given that many young people do not have access to any form of psychological intervention for TS/CTD, we believe that the comparator will outweigh any benefit of standard care (which typically does not include a psychological intervention).

Adverse events will be carefully monitored throughout the trial. Serious adverse events related to any of the two treatments are expected to be very rare. All participants that experience a serious adverse event (regardless of its cause) will be followed-up until the event is resolved, which may include referral to other healthcare services.

To ensure confidentiality, families will receive information about the risks and precautions that are being taken when using communication technology (e.g. encrypted server technology and double authentication login procedures). The study ID will be recorded on all paper datasheets and in the electronic trial database. A hard copy linking patient identity, contact details, screening ID, and study ID for all participants will be kept securely in a locked filing cabinet separate from datasheets. The hard copy can only be accessed by approved members of the research team. All data will be kept secure at all times and maintained in accordance with the requirements of GCP regulations.

The trial will be reported according to the CONSORT and *Consolidated Health Economic Evaluation Reporting Standards* (CHEERS) statements. We plan to write two primary scientific papers for peer review. Manuscript 1 will report on efficacy and cost-effectiveness at the primary endpoint (3-month follow-up). Manuscript 2 will report efficacy results for the long-term follow-up period (up to 12 months post-treatment), also including a long-term health economic evaluation. Additional publications may include analyses of predictors and moderators of treatment outcome. Results will be presented at scientific conferences, communicated to patient organisations, and disseminated to the general public.

## Discussion

This trial will evaluate the efficacy, durability, and cost-effectiveness of an IBT programme for young people with TS/CTD, compared to an active comparator. Two hundred and twenty participants (9–17 years) with TS or CTD will be randomised to receive one of the two treatments. Both treatments will be delivered via the internet during 10–12 weeks and include minimal asynchronous therapist support, primarily via text messages within the IBT platform. Data will be collected at multiple assessment points up to 12 months post-treatment, with the 3-month follow-up being the primary endpoint. The primary outcome is tic severity as measured by the YGTSS-TTSS. Outcome assessors will be blind to treatment allocation at all assessment points. A health economic evaluation of BIP TIC will be performed from multiple perspectives.

The results will be interpreted in light of the potential limitations of the study design. In a scenario where our superiority hypothesis is rejected, it will be difficult to attribute this to lack of efficacy of the intervention. This is because the comparator may also be somewhat effective in the reduction of tics. In fact, education about tics is a core component of all evidence-based treatment manuals for the disorder [33, 34]. However, we decided against including a third, pure waitlist control arm primarily on ethical grounds (i.e. each participant receives an intervention).

If the trial is successful, we will strive to implement the intervention in routine clinical care via Sweden’s national *Stöd och behandling* (English: “*Support and treatment*”) platform [68].

## Trial status

The current full study protocol in use is version 3.0 (Supplementary file 2), dating 3 February 2021. The first trial participant was randomised on 26 April 2019, when protocol version 2.0 was in use. Changes between protocol version 2.0 and 3.0 include as follows: (1) inclusion of an additional exclusion criterion to prevent the inclusion of multiple members (e.g. siblings) of the same family in the trial, (2) a few minor adaptations to the trial procedures due to the COVID-19 pandemic, and (3) added detail to the statistical analysis plan. See the Supplementary file 2 for a full description of all protocol changes. In case further major protocol amendments are needed in the future, these will be communicated to the relevant parties (e.g. ClinicalTrials.gov, this journal).

Inclusion of participants was completed on 9 April 2021. The total number of included participants was 221. The final participant completed treatment on 23 June 2021. This participant is expected to reach the 3-month follow-up assessment (primary endpoint) in September 2021 and the 12-month follow-up assessment (final data collection in the study) in June 2022.

## Supplementary Information

**Additional file 1: Supplementary file 1.** Screenshots of the BIP TIC and comparator interventions, delivered through the BIP platform.

**Additional file 2: Supplementary file 2.** Full study protocol, version 3.0.

**Additional file 3: Supplementary file 3.** SPIRIT 2013 checklist.

**Additional file 4: Supplementary file 4.** Funding documents in Swedish (original) and English (translation).

**Additional file 5: Supplementary file 5.** Ethical approval documents in Swedish (original) and English (translation).

## Data Availability

The data are pseudonymised according to national (Swedish) and European Union legislation and cannot be anonymised and published in an open repository. Participants in the trial consent for their data to be shared with other international researchers for research purposes. The data can be made available upon reasonable request on a case-by-case basis according to the current legislation and ethical permits.

## Abbreviations

0-wk: 0 weeks in to treatment, the equivalent of the treatment start
3FU-12FU: Assessment points 3-12 months after the end of treatment
3-wk-5wk: Assessment points 3-5 weeks in to treatment
ADHD: Attention deficit/hyperactivity disorder
AQ-10: Autism Spectrum Quotient, 10-item version
BIP: Barninternetprojektet (Swedish for “The Child Internet Project”)
BIP TIC: Therapist-guided internet-delivered behaviour therapy (exposure with response prevention) for children and adolescents with Tourette syndrome or chronic tic disorder
BT: Behaviour therapy
C&A-GTS-QOL: Child and Adolescent Gilles de la Tourette Syndrome–Quality of life scale
CBIT: Comprehensive Behavioural Intervention for Tics
CGAS: Children’s Global Assessment Scale
CGI: Clinical Global Impression scales
CGI-I: Clinical Global Impression – Improvement scale
CGI-S: Clinical Global Impression – Severity scale
CHEERS: Consolidated Health Economic Evaluation Reporting Standards
CHU9D: Child Health Utility 9 Dimensions
CONSORT: Consolidated Standards of Reporting Trials
CTD: Chronic tic disorder
DSM-5: 5th edition of the Diagnostic and statistical manual of mental disorders
ERP: Exposure with response prevention
GCP: Good clinical practice
HRT: Habit reversal training
IBT: Internet-delivered behaviour therapy
ITT: Intention-to-treat
iiPAS: Internet Intervention Patient Adherence Scale
KTA: Karolinska Trial Alliance
MINI-KID: Mini-International Neuropsychiatric Interview for children and adolescents
OCD: Obsessive-compulsive disorder
OCD-RD: Obsessive-compulsive and related disorders
OCI-CV: Obsessive-Compulsive Inventory – Child version
ORBIT: Online Remote Behavioural Intervention for Tics
Post: Assessment point after the end of treatment
PTQ: Parent Tic Questionnaire
PUTS: Premonitory Urge for Tics Scale
QALY: Quality-adjusted life year
RCT: Randomised controlled trial
SDV: Source data verification
SMFQ-C: Short Mood and Feelings Questionnaire – Child version
SMFQ-P: Short Mood and Feelings Questionnaire – Parent version
SNAP-IV: Swanson, Nolan, and Pelham rating scale
SPIRIT: Standard Protocol Items: Recommendations for Interventional Trials
TiC-P: Trimbos/iMTA questionnaire for costs associated with psychiatric illness
TS: Tourette syndrome
TTSS: Total Tic Severity Score
WAI-C: Working Alliance Inventory – Child version
WAI-P: Working Alliance Inventory – Parent version
YGTSS: Yale Global Tic Severity Scale
YGTSS-TTSS: Yale Global Tic Severity Scale – Total Tic Severity Score
