# Correction: Efficacy and cost-effectiveness of therapist-guided internet-delivered behaviour therapy for children and adolescents with Tourette syndrome: study protocol for a single-blind randomised controlled trial

**DOI:** 10.1186/s13063-022-06483-7

**Published:** 2022-06-30

**Authors:** Per Andrén, Lorena Fernández de la Cruz, Kayoko Isomura, Fabian Lenhard, Charlotte L. Hall, E. Bethan Davies, Tara Murphy, Chris Hollis, Filipa Sampaio, Inna Feldman, Matteo Bottai, Eva Serlachius, Erik Andersson, David Mataix-Cols

**Affiliations:** 1 Department of Clinical Neuroscience, Centre for Psychiatry Research, Karolinska Institutet, Gävlegatan 22, 113 30 Stockholm, Sweden; 2 Stockholm Health Care Services, Region Stockholm, Stockholm, Sweden; 3 Institute of Mental Health, Mental Health & Clinical Neurosciences, University of Nottingham, Nottingham, UK; 4 NIHR MindTech MedTech Co-operative, Institute of Mental Health, School of Medicine, Mental Health & Clinical Neurosciences, University of Nottingham, Innovation Park, Triumph Road, Nottingham, UK; 5 UCL Great Ormond Street Institute of Child Health (ICH), 30 Guilford Street, London, WC1N 1EH UK; 6 Psychological and Mental Health Services, Great Ormond Street Hospital for Children NHS Foundation Trust, Great Ormond Street, London, UK; 7 NIHR Nottingham Biomedical Research Centre, Institute of Mental Health, Division of Psychiatry and Applied Psychology, University of Nottingham, Innovation Park, Triumph Road, Nottingham, UK; 8 Department of Public Health and Caring Sciences, Uppsala University, Uppsala, Sweden; 9 Unit of Biostatistics, Institute of Environmental Medicine, Karolinska Institutet, Stockholm, Sweden

**Correction: BMC Plant Biol 22, 269 (2022)**   **https:// doi. org/ 10. 1186/ s13063-​021-​05592-z**

Following the publication of the original article [1], we were notified that the second author’s name was incorrectly tagged in the article’s XML.

Incorrect tagging:

GivenName: Lorena Fernández.

FamilyName: de la Cruz.

Correct tagging:

GivenName: Lorena.

FamilyName: Fernández de la Cruz.

The original article has been corrected.
