# Supplementary material for: Efficacy and cost-effectiveness of therapist-guided internet-delivered behaviour therapy for children and adolescents with Tourette syndrome: study protocol for a single-blind randomised controlled trial
Source: Trials. 2021 Sep 30;22:669. doi: 10.1186/s13063-021-05592-z (PMC8481317; doi:10.1186/s13063-021-05592-z)

Supplementary file 1. Screenshots of the BIP TIC and comparator interventions, delivered through the BIP platform.

1. Overview and start page, with list of chapters. This specific screenshot is from BIP TIC, but both treatments share the same appearance.

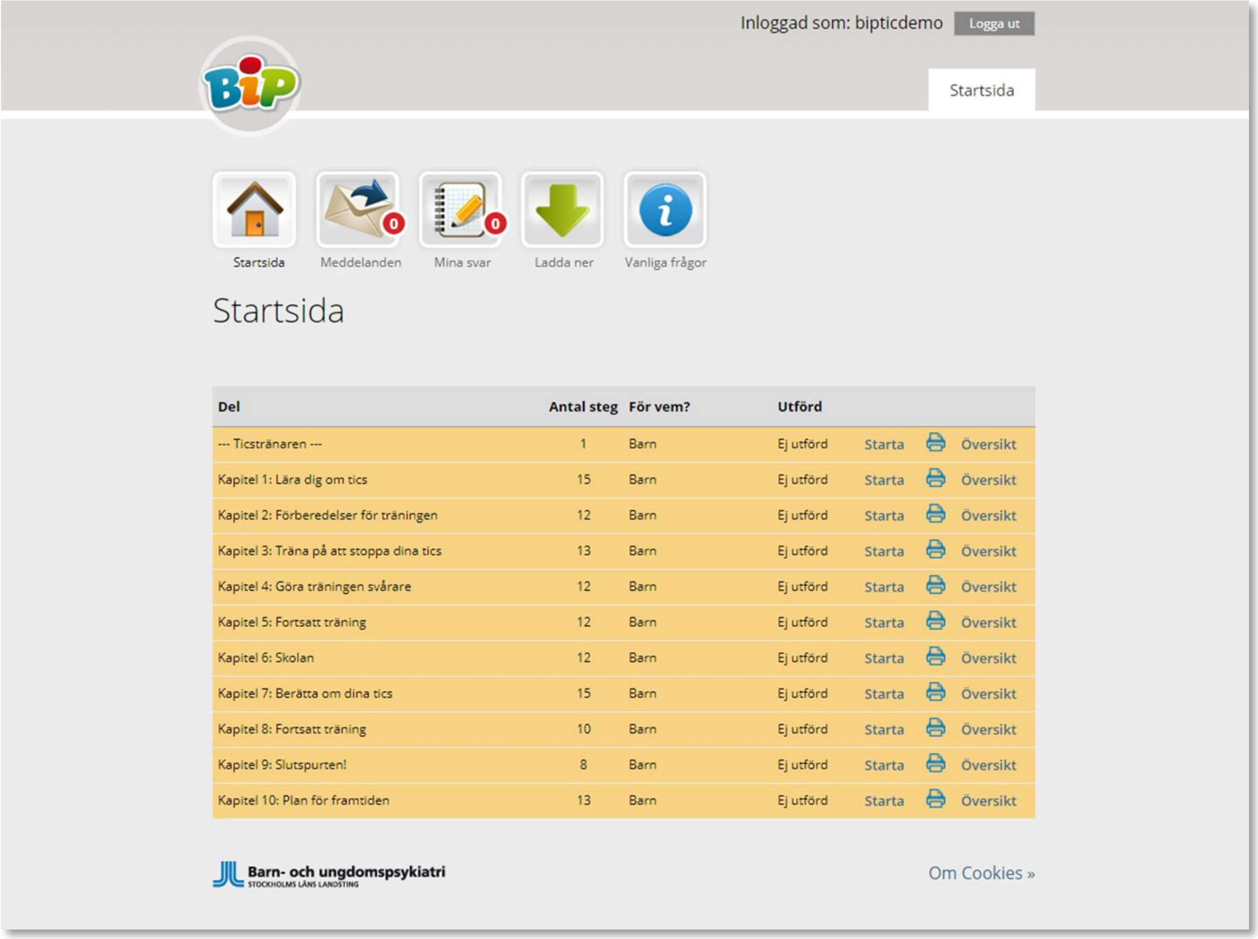

Del	Antal steg	För vem?	Utförd		
--- Ticstränaren ---	1	Barn	Ej utförd	Starta	Översikt
Kapitel 1: Lära dig om tics	15	Barn	Ej utförd	Starta	Översikt
Kapitel 2: Förberedelser för träningen	12	Barn	Ej utförd	Starta	Översikt
Kapitel 3: Träna på att stoppa dina tics	13	Barn	Ej utförd	Starta	Översikt
Kapitel 4: Göra träningen svårare	12	Barn	Ej utförd	Starta	Översikt
Kapitel 5: Fortsatt träning	12	Barn	Ej utförd	Starta	Översikt
Kapitel 6: Skolan	12	Barn	Ej utförd	Starta	Översikt
Kapitel 7: Berätta om dina tics	15	Barn	Ej utförd	Starta	Översikt
Kapitel 8: Fortsatt träning	10	Barn	Ej utförd	Starta	Översikt
Kapitel 9: Slutspurten!	8	Barn	Ej utförd	Starta	Översikt
Kapitel 10: Plan för framtiden	13	Barn	Ej utförd	Starta	Översikt

2. Encrypted messaging function, where the participant can send messages to the therapist. Therapists respond within 48 hours on weekdays. This function is used in both treatments.

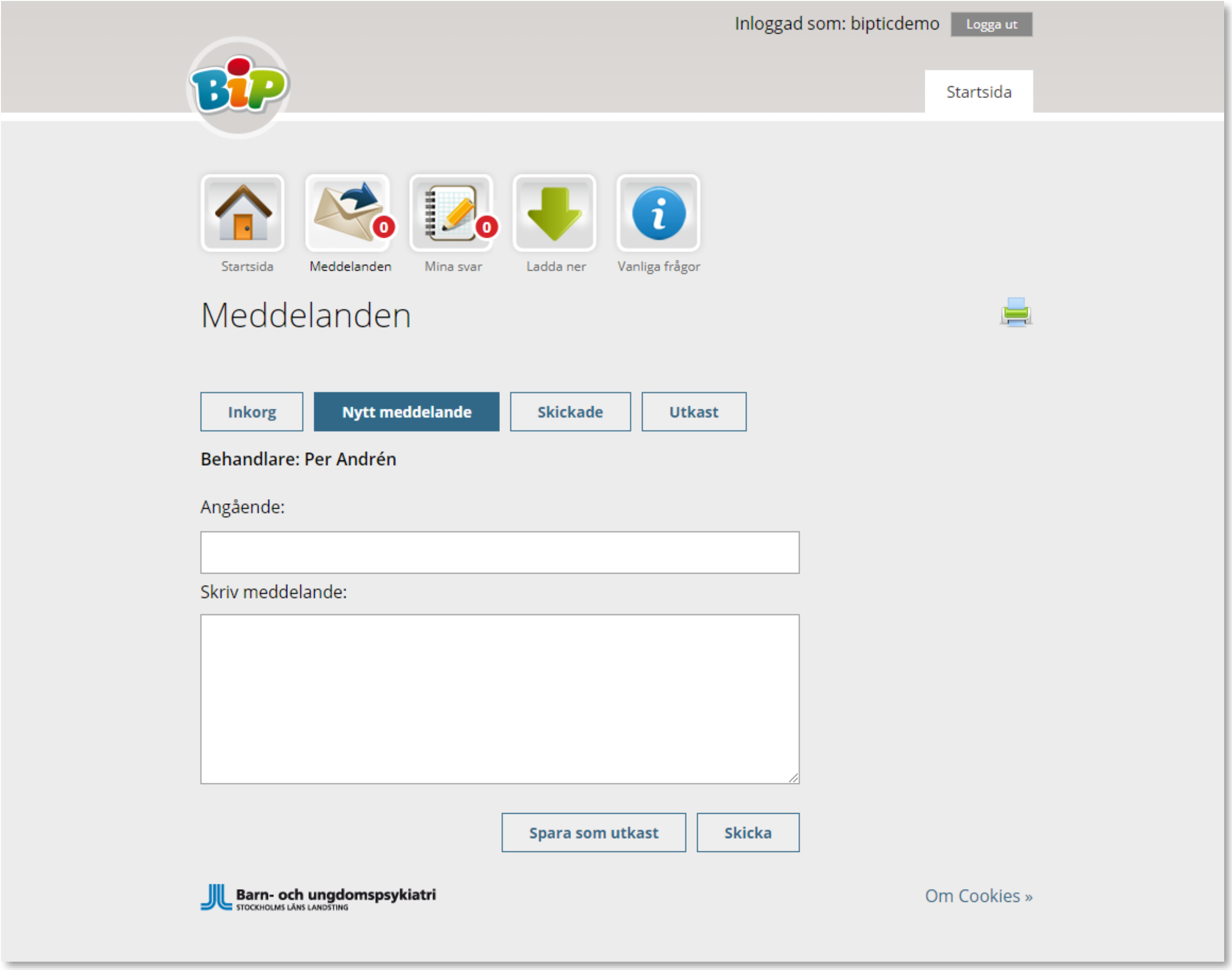

3. Educational films, used for delivering psychoeducation and the treatment rationale. This format is used in both treatments.

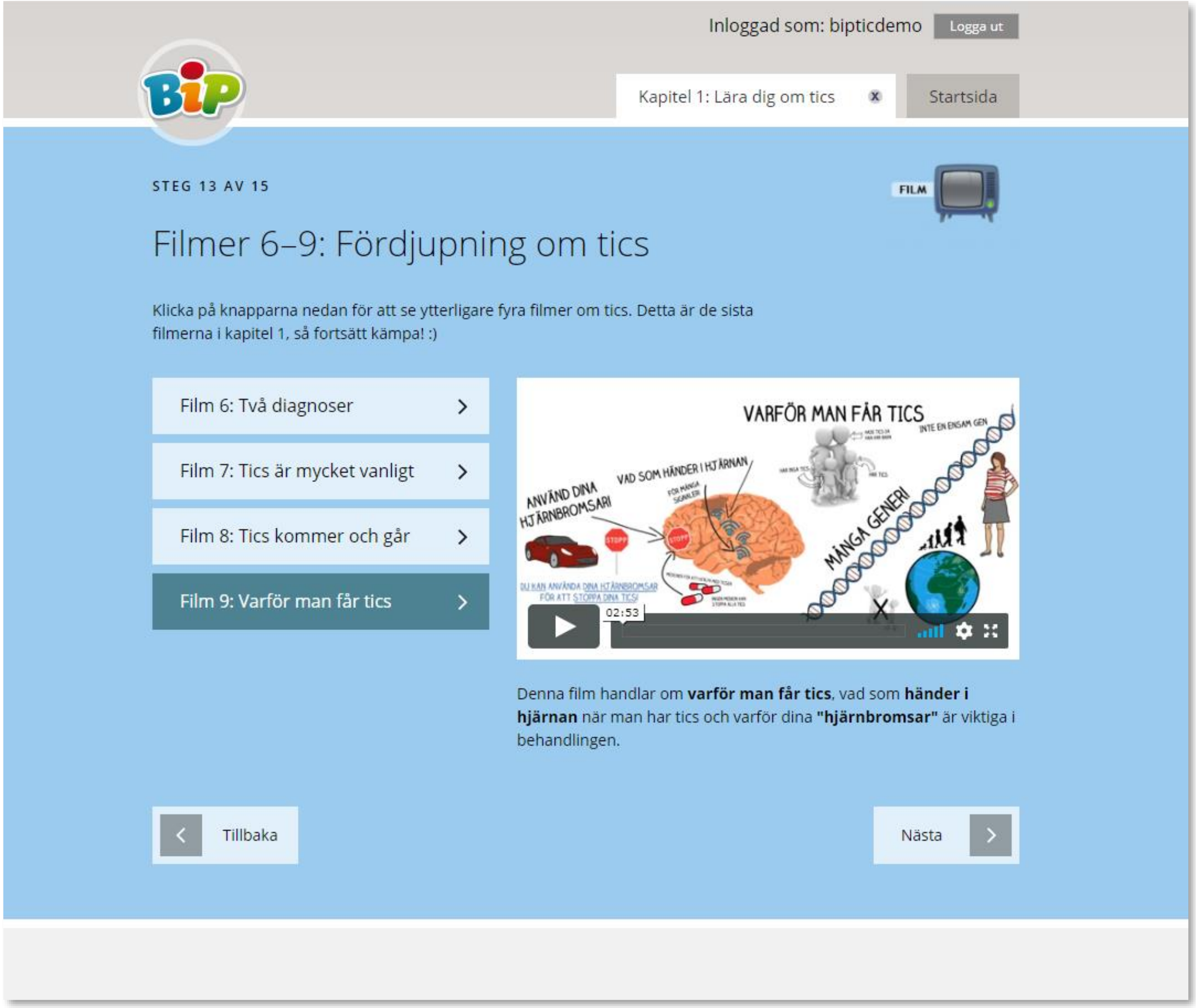

4. Worksheets and exercises. This format is used in both treatments.

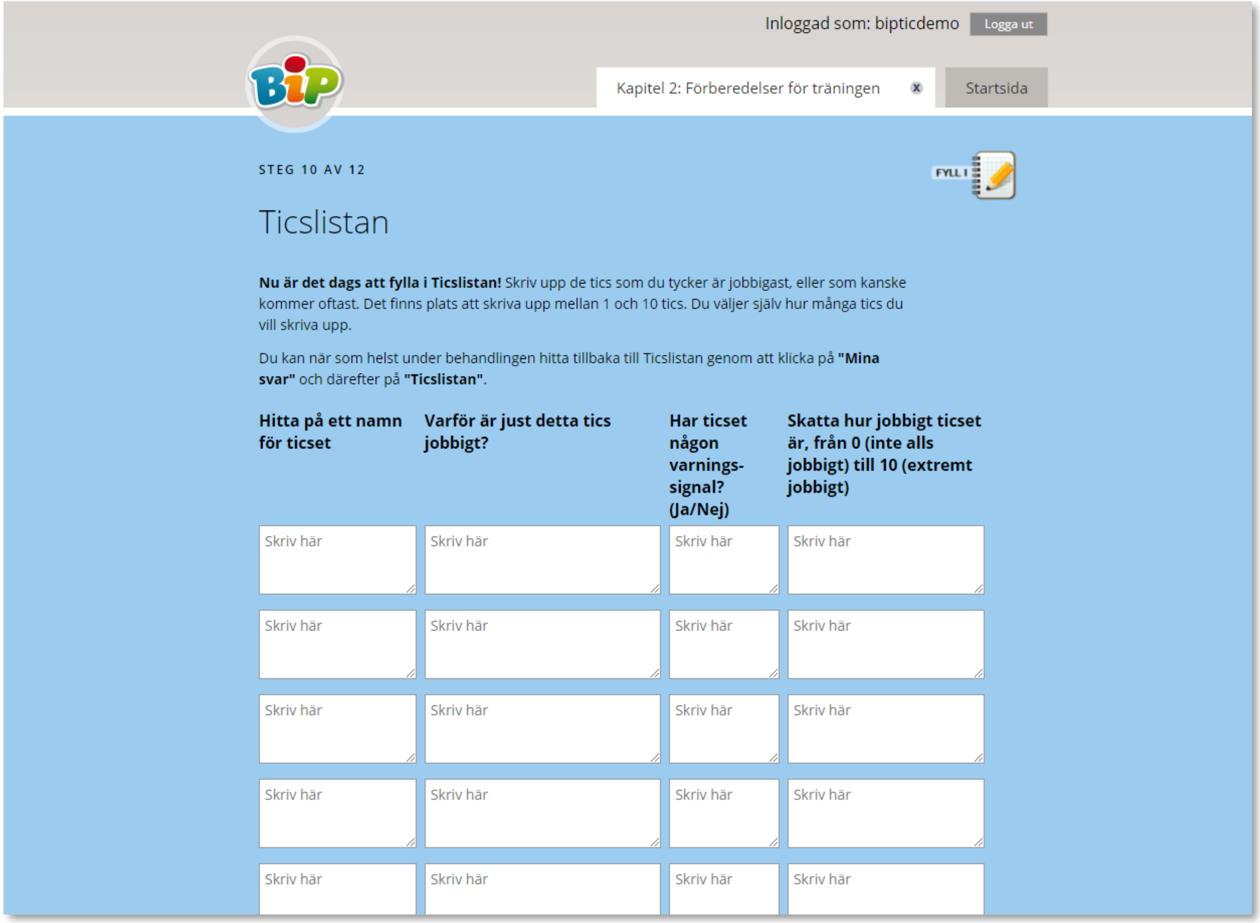

5. Stopwatch with high score lists, to help practice tic suppression. This function is used only in BIP TIC.

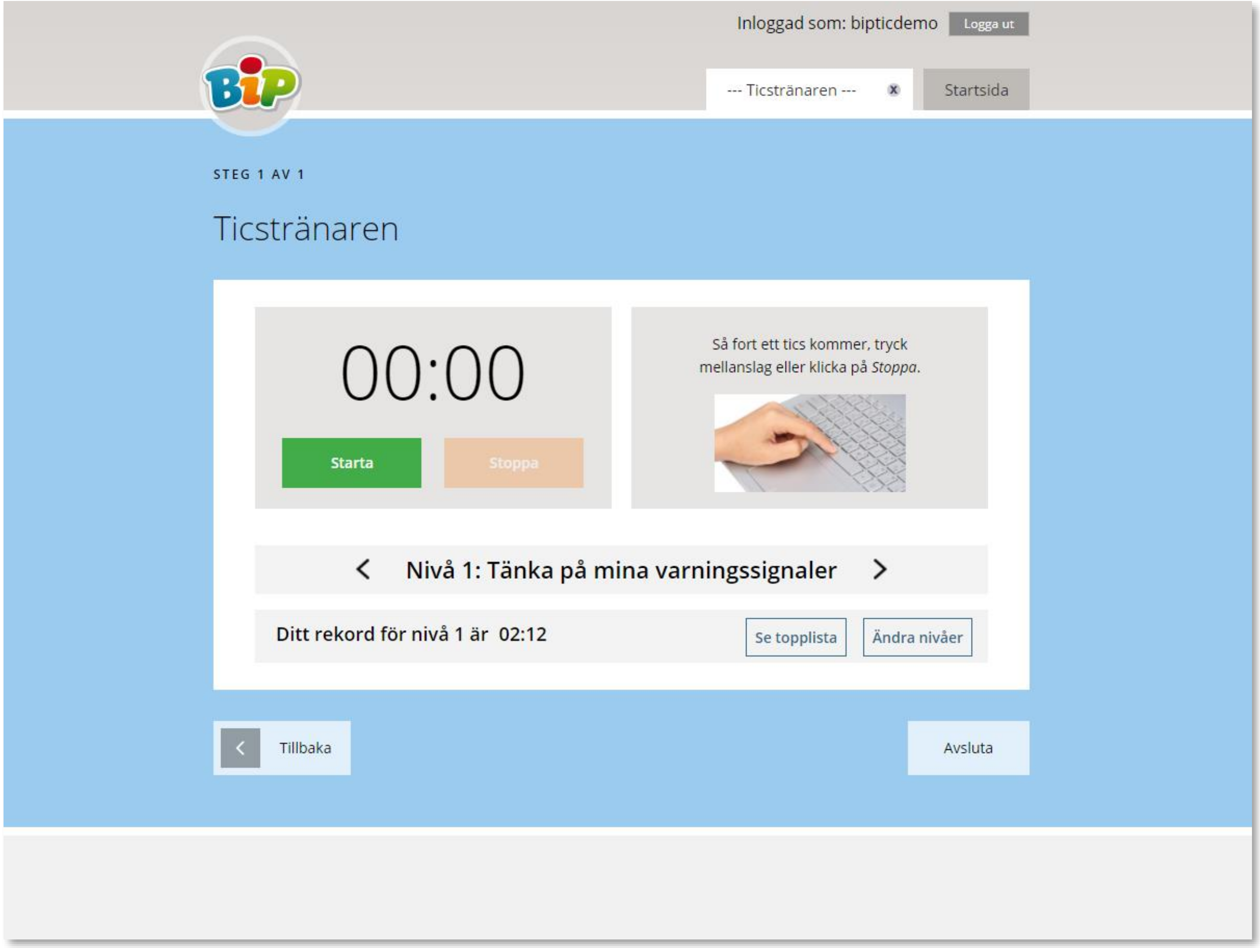

Supplement: Supplementary file 1 — Additional file 1: Supplementary file 1. Screenshots of the BIP TIC and comparator interventions, delivered through the BIP platform. [file 13063_2021_5592_MOESM1_ESM.pdf]
