# Supplementary material for: Efficacy and cost-effectiveness of therapist-guided internet-delivered behaviour therapy for children and adolescents with Tourette syndrome: study protocol for a single-blind randomised controlled trial
Source: Trials. 2021 Sep 30;22:669. doi: 10.1186/s13063-021-05592-z (PMC8481317; doi:10.1186/s13063-021-05592-z)
Supplement: Supplementary file 2 — Additional file 2: Supplementary file 2. Full study protocol, version 3.0. [file 13063_2021_5592_MOESM2_ESM.pdf]

Stockholms läns sjukvårdsområde
STOCKHOLMS LÄNS LANDSTING

BIP TIC RCT PROTOCOL

Long title of the trial	Clinical and cost-effectiveness of internet-delivered behaviour therapy for children and adolescents with Tourette's Disorder: a single-blind randomised controlled trial
Short title of trial	BIP TIC RCT
Version and date of protocol	Version 3.0, 2021-02-03
Sponsor	Barn- och ungdomspsykiatrin (BUP) Stockholm, Stockholms Läns Sjukvårdsområde, SLSO
Funder (s)	Forte (grant number 2017-01066), ALF (grant number 20180093), and Swedish Research Council (grant number 2018-00344)
Trial registration number	ClinicalTrials.gov: NCT03916055
Ethical review	Swedish Ethical Review Authority 2018/1788-31/2 (2019-01670; 2020-04836)
Sites	Child and Adolescent Psychiatry Research Center, Gävlegatan 22, Stockholm, Sweden
Principal investigator:	Professor David Mataix-Cols: david.mataix.cols@ki.se<br>Department of Clinical Neuroscience (CNS), K8,<br>CPF Mataix-Cols<br>Gävlegatan 22, Plan 8, 113 30 Stockholm, Sweden
Sponsor representative:	Göran Rydén

VERSION HISTORY

Version number	Version date	Reason for Change
1.0	20180918	First version
2.0	20190219	Amendment to the Ethical Review Board before trial start. The following changes were made to the protocol: Change of videoconference software, from WebEx to Zoom; change of measure, from CHU9D to KIDSCREEN-10; slight change of the inclusion criteria, highlighting that participants need to be able to receive e-mails (which primarily will be used to receive links to assessments in the data platform BASS and to initiate Zoom videoconference calls); elaboration of the description of the training procedures for the raters of the primary outcome measure YGTSS TTSS; clarification of the section 6.2 Randomisation, enrolment and masking; slight changes in the titles of the treatment chapters (modules); minor changes to section 9.2 Handling of adverse events, to further clarify the role of Karolinska Trial Alliance; addition of funders; and some minor changes to operating procedures (not specifically listed).
2.1	20190416	Added trial registration number. Typos and other small changes. No substantial changes.
2.2	20191126	Specified section regarding eligibility criteria, to not allow the inclusion of two participants with a close relationship to each other (siblings, cousins etc.), to avoid the risk of them being randomised into two different groups, to maintain the integrity of the study design.
2.3	20200911	Due to the COVID-19 pandemic, minimal adaptations to the assessment and consent procedures were implemented from March 2020 (see new section 6.1.3).
3.0	20210203	A detailed statistical and health economic analysis plan (BIP TIC RCT SAP) has been written and added as a new Appendix. The corresponding sections 8.1-8-3 in this study protocol have been updated. A new section (9.5) has been added with information on the reporting of adverse events. Changes to the study team have been noted (health economist Filippa Sampaio has joined the

		team). Minor edits and updates have been made throughout the document.

SIGNATURE

Protocol version number: 3.0

David Mataix-Cols, Principal Investigator (PI)

Date: February 3, 2021

Signature: David Mataix-Cols

Digitally signed by David Mataix-Cols
DN: cn=David Mataix-Cols, o=Karolinska Institutet, ou=Department of Clinical Neuroscience, email=david.mataix.cols@ki.se, c=SE
Date: 2021.02.03 17:57:17 +01'00'

TABLE OF CONTENTS

1 TRIAL PERSONNEL 7
2 SUMMARY 10
3 INTRODUCTION 12
3.1 BACKGROUND 12
3.2 CLINICAL DATA 13
4 OBJECTIVES 15
4.1 PRIMARY AND SECONDARY OBJECTIVES 15
5 PROJECT DESCRIPTION 15
5.1 DESIGN 15
5.2 STUDY SITES 16
5.3 POWER ANALYSIS 17
5.4 INCLUSION AND EXCLUSION CRITERIA 17
5.4.1 INCLUSION CRITERIA 17
5.4.2 EXCLUSION CRITERIA 18
5.5 MEASURES 19
5.5.1 BASELINE MEASURES 19
5.5.2 PRIMARY OUTCOME 20
5.5.3 SECONDARY CLINICIAN-RATED/ENTERED OUTCOMES 22
5.5.4 SECONDARY CHILD-REPORTED OUTCOMES 23
5.5.5 SECONDARY CAREGIVER/PARENT-REPORTED OUTCOMES 25
6 PROCEDURE 26
6.1 RECRUITMENT 26
6.1.1 RECRUITMENT RATE 26
6.1.2 SCREENING AND RECRUITMENT PROCEDURES 27
6.1.3 CHANGES TO PROTOCOL DUE TO THE COVID-19 PANDEMIC 30
6.2 RANDOMISATION, ENROLMENT AND MASKING 31
6.3 ASSESSMENT POINTS FOR EACH OUTCOME 31
6.4 BASELINE ASSESSMENT 33
6.5 TREATMENT 33
6.5.1 BIP TIC 34
6.5.2 ACTIVE CONTROL CONDITION 35
6.5.3 THERAPIST TRAINING, SUPERVISION AND MONITORING 36
6.6 SUBSEQUENT ASSESSMENTS 37

6.6.1 MID-TREATMENT ASSESSMENTS ... 37
6.6.2 POST-TREATMENT ASSESSMENT ... 37
6.6.3 3, 6 AND 12-MONTH FOLLOW-UPS ... 37
6.7 END OF TRIAL ... 37
6.8 PARTICIPANT WITHDRAWAL FROM TRIAL ... 37
6.9 DISCONTINUATION OF TRIAL ... 38
6.10 PROJECT MILESTONES ... 38
6.11 CONCOMITANT INTERVENTIONS ... 38
6.12 QUALITY CONTROL ... 39
7 DATA MANAGEMENT ... 39
7.1 DATA COLLECTION AND HANDLING ... 39
7.1.1 BIP PLATFORM ... 40
7.1.2 BASS ... 40
7.1.3 ZOOM ... 41
8 STATISTICAL ANALYSIS ... 41
8.1 PRIMARY OBJECTIVE: CLINICAL EFFICACY OF BIP TIC ... 41
8.2 SECONDARY OBJECTIVE #1: 12-MONTH DURABILITY OF THE TREATMENT EFFECTS ... 42
8.3 SECONDARY OBJECTIVE #2: HEALTH ECONOMIC EVALUATION OF BIP TIC ... 42
8.4 INTERIM ANALYSIS ... 44
8.5 PREDICTOR AND MODERATOR ANALYSIS ... 44
9 RECORDING AND REPORTING OF ADVERSE EVENTS AND REACTIONS ... 44
9.1 DEFINITIONS ... 44
9.2 EXPECTED ADVERSE EVENTS ... 45
9.3 ASSESSMENT OF ADVERSE EVENTS ... 45
9.4 HANDLING OF ADVERSE EVENTS ... 45
9.5 REPORTING OF ADVERSE EVENTS ... 46
9.6 NOTIFICATION OF SERIOUS BREACHES TO GCP AND/OR THE PROTOCOL ... 46
10 PUBLIC AND PATIENT INVOLVEMENT ... 47
11 DATA SHARING ... 48
12 ETHICAL CONSIDERATIONS ... 48
13 IMPLICATIONS ... 49
14 FINANCE ... 50
15 PUBLICATION PLANS ... 50
16 REFERENCES ... 50

17 APPENDIX……….

1 TRIAL PERSONNEL

Principal Investigator (PI):	Professor David Mataix-Cols<br>Karolinska Institutet<br>Department of Clinical Neuroscience (CNS), K8,<br>Gävlegatan 22, Plan 8 113 30 Stockholm, Sweden<br>david.mataix.cols@ki.se<br>+46 72-398 68 89
Sponsor representative:	Göran Rydén<br>Barn- och ungdomspsykiatrin (BUP) Stockholm, Stockholms Läns Sjukvårdsområde, SLSO<br>Sachsgatan 10, 118 61 Stockholm<br>goran.ryden@sll.se<br>+46 8-514 520 88
Statistician:	Professor Matteo Bottai<br>Karolinska Institutet<br>Institutet för miljömedicin (IMM), C6<br>Biostatistik<br>Box 210 171 77 Stockholm<br>matteo.bottai@ki.se
Health economists:	Dr Inna Feldman<br>Uppsala University<br>Department of Public Health and Caring<br>BMC, Husargatan 3, Uppsala<br>inna.feldman@pubcare.uu.se<br>+46 18-471 65 61
	Dr Filipa Sampaio<br>Uppsala University<br>Department of Public Health and Caring<br>BMC, Husargatan 3, Uppsala<br>filipa.sampaio@pubcare.uu.se
Co-investigators:	Dr Eva Serlachius<br>Karolinska Institutet<br>Department of Clinical Neuroscience (CNS), K8,<br>Gävlegatan 22, Plan 8 113 30 Stockholm, Sweden<br>eva.serlachius@ki.se<br>+46 70 715 52 32
	Dr Lorena Fernández de la Cruz<br>Karolinska Institutet<br>Department of Clinical Neuroscience (CNS), K8,<br>Gävlegatan 22, Plan 8 113 30 Stockholm, Sweden

lorena.fernandez.de.la.cruz@ki.se
+46 76 847 79 99

Dr Fabian Lenhard
Karolinska Institutet
Department of Clinical Neuroscience (CNS), K8,
Gävlegatan 22, Plan 8 113 30 Stockholm, Sweden
fabian.lenhard@ki.se
+46 73 755 59 91

Per Andrén
Karolinska Institutet
Department of Clinical Neuroscience (CNS), K8,
Gävlegatan 22, Plan 8 113 30 Stockholm, Sweden
per.andren@ki.se
+46 70 435 17 40

Dr Erik Andersson
Karolinska Institutet
Department of Clinical Neuroscience (CNS), K8,
Nobels Väg 9, 171 77 Stockholm
erik.m.andersson@ki.se
+46 73-671 63 35

Dr Kayoko Isomura
Karolinska Institutet
Department of Clinical Neuroscience (CNS), K8,
Gävlegatan 22, Plan 8 113 30 Stockholm, Sweden
kayoko.isomura@ki.se
+46 76-009 52 02

Professor Chris Hollis
University of Nottingham
Division of Psychiatry and Applied Psychology,
E Floor, South Block, Queen's Medical Centre, Nottingham, NG7 2UH
chris.hollis@nottingham.ac.uk
+44 115 8230258

Dr Charlotte Hall
University of Nottingham
Institute of Mental Health
Floor B, Innovation Park, Triumph Road, Nottingham, NG7 2TU
charlotte.hall@nottingham.ac.uk
+44 115 8232438

Dr Tara Murphy
Great Ormond Street Hospital for Children NHS Foundation Trust
Great Ormond Street, London WC1N 3JH
tara@thegrowingbrain.com
+44 20 7405 9200

Dr E Bethan Davies
NIHR MindTech Medtech Co-operative, University of Nottingham
Floor B, Institute of Mental Health
Innovation Park, Triumph Road, Nottingham, United Kingdom NG7 2TU
bethan.davies@nottingham.ac.uk
+44 115 7484238

2 SUMMARY

**Title:**	Clinical and cost-effectiveness of internet-delivered behaviour therapy for children and adolescents with Tourette's Disorder: a single-blind randomised controlled trial
**Short title:**	Internet-delivered behaviour therapy for young people with Tourette's Disorder
**Objectives:**	Primary objective: To determine the clinical efficacy of *BIP TIC* (a therapist-and parent-guided, internet-delivered behavioural intervention for Tourette's Disorder [TD]), for reducing tic severity (as measured by the primary outcome variable YGTSS Total Tic Severity Score) in children and adolescents with TD, compared with a control intervention (therapist- and parent-guided internet-delivered education on tics).<br><br>Secondary objectives: To establish the 12-month durability of the treatment effects, and to assess the cost-effectiveness of *BIP TIC*, compared with therapist- and parent-guided online education, from multiple perspectives.
**Type of trial:**	Single-blind parallel-group randomised controlled superiority trial.
**Trial design and methods:**	All potential participants are initially screened via the telephone, or in some cases at the clinic. This is followed by an inclusion assessment conducted either at the clinic or via the videoconference software Zoom. Participants who are eligible and have consented will be randomised into one of two trial arms. In the experimental arm, participants receive 10 weeks of therapist- and parent-guided internet-delivered exposure and response prevention (ERP). In the control arm, participants receive 10 weeks of therapist- and parent-guided internet-delivered education on tics.<br><br>Participants will complete outcome measures at baseline, mid-treatment, post-treatment, and 3, 6 and 12 months post-treatment. The primary outcome variable is the Total Tic Severity Score (TTSS) on the Yale Global Tic Severity Scale (YGTSS), and the primary endpoint is the follow-up 3-months post-treatment.<br><br>Secondary outcomes include measures of tics (PTQ), OCD (OCI-CV), mood (SMFQ), adverse events, treatment credibility and satisfaction, among others. Quality of life (KIDSCREEN-10) and resource use (modified TiC-P) will also be collected for the health economic evaluation. Researcher completed measures of global functioning (CGI-

	Improvement; CGAS) will also be recorded. Follow-up assessments will be conducted at the clinic or via Zoom videoconference software, in both cases complemented with online questionnaires.
**Participant time in trial:**	Approximately 15 months.
**Total trial duration:**	Approximately 36-months (from first participant enrolled to last participant follow-up).
**Planned trial sites:**	All assessments and delivery of treatment will be administrated from a single site in Stockholm, the CAP Research Center. Several collaborating counties all across Sweden will assist on referring potential participants to the Stockholm site for inclusion in the trial.
**Sample:**	220 participants.
**Brief eligibility criteria:**	Eligible participants will be aged 9-17 years and have a DSM-5 diagnosis of TD or Persistent (Chronic) Motor or Vocal Tic Disorder. Exclusion criteria include receiving behaviour therapy within the past 12 months, recent changes to tic medication, and a lifetime history of intellectual disability, autism spectrum disorder, bipolar disorder, or psychosis, among others. Full details are presented in section 5.4.
**Statistical methodology and analysis:**	Data will be analysed using a pre-specified intention-to-treat statistical analysis plan. The primary outcome will be analysed using a linear quantile mixed model. Secondary outcomes will be analysed using analogous methods to the primary outcome.

3 INTRODUCTION

3.1 BACKGROUND

Tourette's Disorder (TD) is a common, disabling, childhood-onset condition affecting up to 1% of young people, and is associated with high levels of distress, psychosocial impairment and reduced quality of life [1]. The majority of patients additionally experience comorbidities such as attention deficit hyperactivity disorder (ADHD), obsessive-compulsive disorder (OCD), depression, anxiety, and self-injurious behaviour [2], making interventions complex. Evidence-based interventions for the treatment of tics in children and adolescents with TD include both pharmacological treatment and behaviour therapy (BT) [1, 3-5]. Two different types of behaviour therapy, *habit reversal training* (HRT) and *exposure and response prevention* (ERP), are both effective treatments for tics [3, 4], and the comprehensive behavioural intervention for tics (CBIT) package based on HRT shows similar efficacy to medication [4]. While there is no NICE guidance for the management of tics in children and young people with TD, existing European guidelines [3] and a recent UK Health Technology Assessment (HTA) evidence synthesis [1] recommend that BT should be offered first-line for tics in children and adolescents in a stepped-care approach. Despite these recommendations, only one in five young people with TD are currently able to access BT for tics, while over 70% receive medication[6], which is associated with a significant risk of adverse effects including weight gain and sedation [4, 5]. Furthermore, those young people who manage to access BT typically receive four or fewer face-to-face therapy sessions, less than half the number recommended.

The majority of young people surveyed with TD request greater access to behavioural interventions [7]. However, in Sweden, and across the world, there is a lack of expert therapists trained to deliver BT for TD. Therapists are distributed, requiring long distance travel to receive treatment, which is expensive, disruptive, and time-consuming for patients.

Over the last decade, internet-delivered cognitive behaviour therapy (ICBT) has been developed which enables us to offer less therapist intensive, but effective, interventions over long distances, which can increase the availability of evidence-based treatments. In ICBT, the patient logs in to a secure online platform, where the treatment is presented as a series of self-help texts and audio-visual materials, accompanied by homework assignments. During the entire treatment, a therapist provides guidance and gives feedback through a built-in email system. Crucially, ICBT only requires a fraction of the therapist time associated with regular CBT. ICBT is efficacious and cost-effective for a wide range of mental health and functional disorders in adults.[8, 9]

Barninternetprojektet (BIP) is an eHealth platform specifically designed for young people and their parents (www.bup.se/bip/). BIP has been shown to be an efficacious and cost-effective method of delivering treatment for children and adolescents with anxiety disorders,[10] specific phobia,[11] OCD,[12, 13] and functional gastrointestinal disorders.[14] Given the

dramatic shortage of help for TD patients, and the success of previous BIP trials, our team decided to develop BIP TIC, an internet-delivered behavioural intervention for young people with TD, and to evaluate its feasibility in a pilot randomised controlled trial.[15] The results showed that it is feasible to remotely deliver a behavioural intervention for TD via the internet, with high patient and parent satisfaction, requiring only minimal therapist time.

3.2 CLINICAL DATA

For many years, antipsychotics and noradrenergic agents were considered the first line treatment for tics. Although there is randomised controlled trial (RCT) evidence of short-term effectiveness of these drugs for treating tics, these drugs are often associated with significant adverse effects [16], and now current European guidelines are recommending the use of BT as first-line treatment for tics in a stepped-care approach [3].

The effectiveness of BT for reducing tics has been well established [1], with systematic reviews demonstrating a similar magnitude of effect for BT compared to pharmaceutical interventions [1, 4]. Commonly used interventions include: *habit reversal training (HRT),* where patients are taught to implement an action referred to as a competing response when a premonitory urge to tic is experienced; *exposure and response prevention (ERP),* where patients practice to tolerate premonitory urge sensations and suppress their tics. The largest trial used a program referred to as *comprehensive behavioural intervention for tics (CBIT),* which includes HRT and adds additional elements of relaxation training, functional analyses (identification of situations which could exacerbate tics) and social support[17]. A meta-analysis conducted by Hollis et al. showed clear evidence of effectiveness for treatment with HRT/CBIT for tics [1]. Additionally, although less studies have evaluated ERP, the evidence from available studies suggest both HRT and ERP may be equally effective in reducing tics[3]. An important benefit of BT is further the low occurrence of side effects (compared to medication).

Although BT is a recommended treatment for tics, there are a lack of trained therapists across many European countries, including Sweden, leaving many patients unable to access these treatments. A survey of 295 parents of children with TD identified that many families felt healthcare professionals were not knowledgeable about TD. Specifically, respondents noted the struggle to access limited BT resources, with 76% of parents saying they would want BT to be available for their child, highlighting the need for improved access to BT for TD.[7]

Across diagnostic conditions, studies have shown efficacy of ICBT compared to no-treatment as well as face-to-face treatment control conditions [18, 19], with an estimation of up to 50% in cost savings [19]. Therapist guidance during ICBT has been shown to be an important contributing factor to determining outcome. Although self-guided ICBT is superficially attractive, given the very low costs of implementation, research evidence demonstrates very low adherence rates, with therapist-guided ICBT having significantly greater clinical- and cost-effectiveness [20, 21].

There are some clinical data to support the use of our BIP platform (therapist guided ICBT for young people and their parents). An RCT comparing ICBT and a waiting list control in adolescents with OCD, found a significant reduction in OCD symptoms 3-months post-treatment [22]. Additionally, there were no reported adverse events, and participants were generally satisfied with the format of treatment. Symptom reduction was also noted in an RCT using the BIP platform for anxiety [23], as well as in a pilot trial using BIP for specific phobia [24]. These findings together support the use of therapist guided ICBT delivered via the BIP platform.

There is little research evidence regarding the effectiveness of ICBT and TD. In a recent review of digital health interventions, Hollis et al. found that the majority of interventions have been designed to help children and young people at risk for developing, or with a diagnosis of an anxiety disorder and/or depression, with areas such as TD being largely overlooked [25]. Some evidence is available though, including two pilot RCTs comparing videoconference delivered CBIT (e.g. Skype) to traditional face-to-face delivery in children with TD. Results showed significant tic reduction for both groups, with no difference between the modes of delivery [26, 27]. The method of delivering CBIT was rated as highly acceptable by the participants [26].

Delivering BT via videoconference might solve the long travel distances, but still demands the same amount of specialist therapists (providing the treatment in real time). Following the success of previous ICBT trials using the BIP platform, our team decided to develop BIP TIC: a therapist-guided internet-delivered behavioural intervention for young people with TD, and to evaluate its feasibility in a pilot randomised controlled trial [15]. In this trial, we randomised 23 participants aged 8-16 years to receive either therapist-guided internet-delivered HRT (called BIP TIC HRT) or therapist-guided internet-delivered ERP (called BIP TIC ERP). Participants in both groups showed improvement 3-months after treatment completion, but only participants in the BIP TIC ERP arm showed significantly reduced tic severity, as measured by the primary outcome measure Total Tic Severity Score, of the Yale Global Tic Severity Scale (YGTSS). Although not a fully powered-trial, the findings show that BIP TIC ERP may be effective in reducing tics. Furthermore, we found no severe adverse events. There were no drop-outs and no data loss at any of the assessment points, and 83% of parents and children rated the treatment as *good* or *very good*, indicating the trial was highly acceptable. Therapist time (consisting of e-mail contact within the BIP platform, and occasional phone calls) was about 25 minutes per patient and week, considerably less than the traditional weekly 60-minute-session in face-to-face or videoconference BT. In conclusion, the results showed that it is feasible to remotely deliver a behavioural intervention for TD via the internet, with high patient and parent satisfaction, requiring only minimal therapist time (making it potentially more available and cost-effective than videoconference delivered BT).

4 OBJECTIVES

4.1 PRIMARY AND SECONDARY OBJECTIVES

Primary:

1. To determine the clinical efficacy of BIP TIC (ERP-version) for reducing tic severity (as measured by the YGTSS Total Tic Severity Score [28]) in children and adolescents with TD and Persistent (Chronic) Motor or Vocal Tic Disorder, compared with an active control intervention (therapist- and parent-guided internet-delivered education on tics). The primary endpoint is the follow-up 3 months post-treatment.

Secondary:

2. To establish the 12-month durability of the treatment effects.
3. To conduct a health-economic evaluation of BIP TIC (ERP-version), compared with therapist- and parent-guided internet-delivered education on tics, from multiple perspectives, both in the short term (primary endpoint) and the long term (12-month follow-up).

5 PROJECT DESCRIPTION

5.1 DESIGN

A parallel-group, single blind, randomised controlled superiority trial for children and adolescents with TD or Persistent (Chronic) Motor or Vocal Tic Disorder. Participants will be randomised to receive 10 weeks of BIP TIC (therapist- and parent-guided internet-delivered behaviour therapy) or an active control condition (therapist- and parent-guided internet-delivered education on tics). The control condition aims to control for online access to basic information on tics, online therapist support, and homework assignments.

The default treatment length is 10 weeks, but if certain reasons (e.g. illness or holidays) the design allows participants to pause their therapist-support for a maximum of two weeks, which extends the treatment length to a maximum of 12 weeks.

Participants will be followed-up at mid-treatment, post-treatment, and follow-ups 3, 6 and 12 months post-treatment. The post-treatment and 3-month follow-up are “intention-to-treat” assessment points, where participants are encouraged not to change medication or start alternative psychological treatment for their tics. The 6- and 12-month follow-ups are naturalistic follow-ups, participants may be using alternative treatments in accordance to standard practice recommended by their usual treating clinician. Assessors will be blind to treatment allocation at all assessment points. A flow chart of the trial design is shown in **Figure 1**.

Figure 1. CONSORT flow diagram.

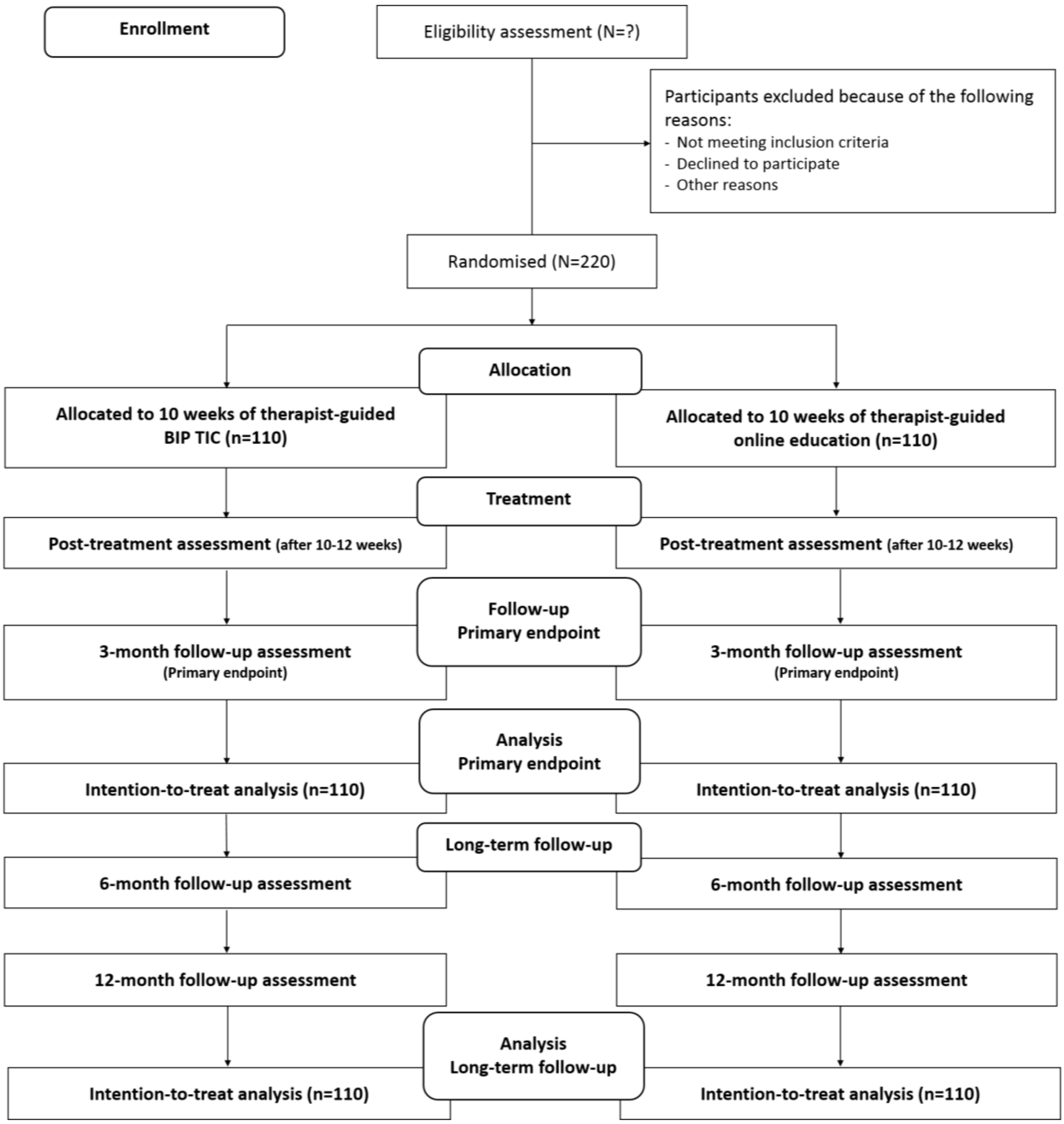

5.2 STUDY SITES

The project is a collaboration between the Department of Clinical Neuroscience at Karolinska Institutet and the Child and Adolescent Mental Health Service (Barn- och ungdomspsykiatrin; BUP) in Stockholm.

All assessments and delivery of treatment will be administrated from a single site in Stockholm, the CAP Research Center, at Gävlegatan 22. Participants will formally be considered as patients at the clinic *BUP Klinisk forskningsenhet (KFE)* within BUP Stockholm,

Stockholm läns landsting, from enrolment to the 12-month follow-up. Several collaborating counties across Sweden will assist on referring potential participants to the Stockholm site for inclusion in the trial. Swedish county councils that have, to date, agreed to participate as recruitment sites are (names of key collaborators in brackets): Region Gävleborg (Dr Najah Khalifa), Västra Götalandsregionen (Dr Lena Wallin/Karin Mellin), Västerbottens läns landsting (Dr Linda Halldner-Henriksson), Landstinget Västmanland (Prof Kent Nilsson), Region Jämtland Härjedalen (Dr Mikael Lec-Alsén), Landstinget Dalarna (Dr Anna Ståhlkloo), Region Skåne (Matti Cervin) and Landstinget Västernorrland. Additional counties will be invited to participate as the study progresses, with the aim to have full national coverage.

5.3 POWER ANALYSIS

Due to our primary and secondary outcome variables being integer-valued (ordinal) in nature, we estimated the power for the difference in *median* outcome between the two treatment groups at the primary endpoint using data from our pilot trial [15]. Specifically, we used a Wald test for the coefficient of the interaction term in a linear quantile random intercept model for the median of the outcome [29-31]. The model contained the intercept, the binary treatment indicator (BIP TIC, control condition), the numeric time variable (baseline, post-treatment, 3-month follow-up), and the treatment-by-time interaction term. We calculated the power under different samples sizes and differences in median outcome between the two treatment groups at the primary endpoint. For each combination of sample size and difference, we simulated 500 samples under a random intercept model with normal intercept and normal residual error. The regression parameters and variance components were obtained from our pilot data. The regression coefficients were 28.56 for the intercept, and –3.11 for time, while the standard deviation of the random intercept was 2.95, and that of the residual was 2.04. These calculations showed that with 200 patients (100 in each arm) we would have 97% power to detect a statistically significant difference in medians of 3 points on the YGTSS Total Tic Severity Score at the primary endpoint. Allowing for a 10% dropout rate, this trial will aim to recruit up to 220 participants.

5.4 INCLUSION AND EXCLUSION CRITERIA

5.4.1 INCLUSION CRITERIA

1) Aged 9 to 17 years.
   - Confirmed by the caregiver and subsequently by the patient record system *TakeCare*.
2) A diagnosis of TD or Persistent (Chronic) Motor or Vocal Tic Disorder, based on the 5th edition of the Diagnostic and Statistical Manual of Mental Disorders [DSM-5; 32].
   - Confirmed by the assessor at the face-to-face or videoconference inclusion assessment.
3) A Total Tic Severity Score (TTSS) of >15 (or >10 if only motor or vocal tics, but not both, have been present the last week) on the YGTSS [YGTSS; 28].

- Confirmed by the assessor at the face-to-face or videoconference inclusion assessment.

4) A minimum of one available caregiver (parent) to support the child/adolescent throughout the treatment.
    - Confirmed by the caregiver at the telephone screening or/and face-to-face or videoconference inclusion assessment.
5) Regular access to a desktop or laptop computer connected to the internet, with the ability to receive e-mails, as well as a mobile phone to receive SMS (one of each is enough per family).
    - Confirmed by the caregiver at the telephone screening or/and face-to-face or videoconference inclusion assessment.

5.4.2 EXCLUSION CRITERIA

1) Previous behaviour therapy for tics, for a minimum of eight sessions with a qualified therapist within the 12 months prior to assessment.
    - Confirmed by the caregiver at the telephone screening or/and face-to-face or videoconference inclusion assessment.
2) Simultaneous psychological treatment for TD or Persistent (Chronic) Motor or Vocal Tic Disorder.
    - Confirmed by the caregiver at the telephone screening or/and face-to-face or videoconference inclusion assessment.
3) Initiation or adjustment of any psychotropic medication for TD or Persistent (Chronic) Motor or Vocal Tic Disorder within the eight weeks prior to assessment.
    - Confirmed by the caregiver at the telephone screening or/and face-to-face or videoconference inclusion assessment. Also followed up at each assessment point in the trial (except mid-treatment).
4) A diagnosis of organic brain disorder, intellectual disability, autism spectrum disorder, psychosis, bipolar disorder, anorexia nervosa, or alcohol/substance dependence.
    - Confirmed by the assessor at the telephone screening or/and face-to-face or videoconference inclusion assessment, with help of information from the caregiver and the child/adolescent, and the MINI-KID interview.
5) Immediate risk to self or others, requiring urgent medical attention, such as suicidality, or self-injurious tics.
    - Confirmed by the assessor at the telephone screening or/and face-to-face or videoconference inclusion assessment.
6) Child or caregiver (parent) not able to read and communicate in Swedish.
    - Confirmed by the caregiver or assessor at the telephone screening or/and face-to-face or videoconference inclusion assessment.

Related to above, it is not possible to be included into the trial if a potential participant has a close relationship to an already included participant (e.g. sibling, cousin), to avoid being

randomised into two different groups, with the risk of information "leaking" between the groups. This is important for the integrity of the controlled study design.

5.5 MEASURES

Clinician-rated/administered measures will first be recorded on paper, then entered in to the trial database, called BASS. Child- and caregiver/parent-reported measures will be completed via the internet directly into BASS. More information about BASS can be found in section 7.1.2. For the specific time points of administration for each measure, see section 6.3 (**Table 1**).

5.5.1 BASELINE MEASURES

Self-referral questionnaire
Technically a tool for recruitment rather than an outcome measure, the child/adolescent (probably the caregiver/parent in the majority of cases) answers a few questions when signing up their interest for the trial. The questions ask about relation (child/adolescent or caregiver/parent), child/adolescent's name, caregiver's name, telephone number for contact, time of the day when they prefer to be contacted, and how they learned about the trial.

Demographic data – clinician-entered
Following the face-to-face or videoconference inclusion assessment, the assessor enters baseline information into the trial database. This includes information on screening number, age, sex, primary responsible caregiver assisting during the treatment, tic diagnosis, comorbid diagnoses, distance to the clinic, age of onset for motor and vocal tics respectively, history of suicide attempt, family history of tic disorders, family history of other psychiatric disorders, current medication for tic disorder or other psychiatric disorder and previous psychological treatment for tic disorder.

Demographic data – parent-reported
At baseline, the caregiver/parent will answer a questionnaire designed by the research team, asking about the caregiver's relation to the child, who the child lives with, if the child has any siblings, the caregivers' educational level, the caregivers' occupation, where the child and caregivers are born, how the caregiver learned about the trial, if the child has been in previous contact with health care services due to her/his tics, or other psychiatric symptoms. The questionnaire takes approximately 5 minutes to complete.

Mini International Neuropsychiatric Interview for Children and Adolescents (MINI-KID)
The MINI-KID [33] is a short structured diagnostic interview for DSM-IV and ICD-10 psychiatric disorders in children and adolescents. It will be used to assess psychiatric comorbidity at baseline and may be used to exclude participants. All clinicians will receive training in MINI-KID prior to the trial.

Obsessive-compulsive disorder and related disorders (OCD-RD)

The OCD-RD is a short diagnostic interview originally based on the Structured Clinical Interview (SCID) for DSM-IV, but adapted by our research team to align with the DSM-5 criteria for OCD, body dysmorphic disorder, hoarding disorder, trichotillomania and excoriation disorder. Additionally, it also screens for olfactory reference syndrome and paediatric acute-onset neuropsychiatric syndrome. It will be used alongside the MINI-KID to gather additional information on psychiatric comorbidity at baseline.

Autism Spectrum Quotient (AQ-10)

AQ-10 [34] is a brief 10-item scale that is used to screen for autistic symptoms. The scale is divided into two separate versions, for children 4 to 11 years and adolescents 12 to 17 years. The questionnaire takes approximately 5 minutes to complete.

Swanson, Nolan, and Pelham Rating Scale (SNAP-IV)

The SNAP-IV [35] is a behavioural rating scale that employs the core symptoms of ADHD and oppositional defiance disorder (ODD), as defined by the 4th edition of the Diagnostic and Statistical Manual of Mental Disorders (DSM-IV) [36]. SNAP-IV consists of 30 items that are rated on a 4-point scale (not at all, just a little, quite a bit, very much). The items are divided between three sub-scales: inattention (9 items), hyperactivity/impulsivity (9 items), and oppositional (8 items). Items for inattention and hyperactivity/impulsivity can be combined to also create a “combined ADHD” score. The questionnaire has established validity and reliability [37]. The questionnaire takes approximately 5 minutes to complete.

Premonitory Urge for Tics Scale (PUTS)

The PUTS [38] is a self-reported measure specifically designed to measure the current frequency of different types of premonitory urges (sensations commonly preceding tics) in patients with tic disorders. PUTS consists of 9 items, scored on a scale from 1 to 4 (not at all – very much), with a total score ranging from 9 to 36. The questionnaire has established validity and reliability [38]. The questionnaire takes less than 5 minutes to complete.

5.5.2 PRIMARY OUTCOME

This trial’s primary outcome is tic severity, as measured by the **Total Tic Severity Score (TTSS)** of the Yale Global Tic Severity Scale (YGTSS).[28] This semi-structured interview is the gold standard in the TD field, which will facilitate comparability with previous trials of behavioural and pharmacological interventions for TD. While other outcomes are also relevant, such as tic-related impairment or quality of life, deviating from the gold standard measure would make comparisons to previous studies difficult. For this reason, impairment and quality of life are secondary outcomes in this trial.

First, the **YGTSS symptom checklist** is administered, which lists 46 tic disorder symptoms. This not a traditional outcome measure per se, but the information is needed to subsequently score the TTSS. The checklist information will be saved for future post hoc analyses connected to the presence of certain categories of tics.

Second, the YGTSS TTSS is scored. The TTSS is derived by adding the Total Motor Tic Score (5 items, score range 0-25) and the Total Phonic Tic Score (5 items; score range 0-25), resulting in an ordinal variable ranging from 0-50 points.

We will take extensive steps to minimise measurement bias of the primary outcome. All raters of YGTSS will be trained according to the procedures below. The training will be supervised by a clinical expert, the trial coordinator Per Andrén (PA).

1) Training will consist of attending a short lecture by PA, where the YGTSS is explained. The raters will also read a short instructional text describing how the YGTSS is used within this trial.

2) Raters will then view at least 3 pre-recorded YGTSS assessments. They will be asked to rate these, however this will be solely for training purposes. Raters are encouraged to make a list of any questions/queries. The questions and ratings will then be discussed with PA.

3) The next step will be a testing phase where 3 different YGTSS assessments will be used against an expert rater (PA) to determine the extent of agreement. In line with the methodology reported by Jeon et al. [39], the raters have to be within 15% of the expert rater for the **Total Motor Tic score**, the **Total Vocal Tic score** and the **Total Tic Score** on each of the 3 recordings. The 15% will always be rounded up in cases where 15% of a score results in a score which is not a whole integer (i.e. 15% of 25 = 3.75 points, this would be rounded up to 4 points). The 15% can be in either direction of the score.

4) Raters who do not meet the criteria will be given additional training and asked to re-score the same YGTSS assessments until the specified agreement criteria are met.

5) The raters' agreement with the expert rater will be re-assessed every 6 months during the trial. The expert rater will rate 1 recent YGTSS assessment (video recording) from each rater, and compare his expert rating with the rater's original rating. If any rater falls outside the 15% agreement with the expert rater, they will be required to engage in further training, as and when this is appropriate during the trial.

We will ask the participants' permission to video record all YGTSS assessments for possible spot checks of the methodology and to measure inter-rater reliability.

YGTSS interviews will be conducted face-to-face at the clinic, or remotely via videoconference software. If technical problems with post-treatment or follow-up assessments, they will instead be conducted via telephone (with the family on speaker telephone). When possible, the post- and follow-up assessments will be conducted by the same blind assessor (for the

same participant). The YGTSS takes between 15 to 35 minutes to administer (longer time at baseline than at follow-ups).

5.5.3 SECONDARY CLINICIAN-RATED/ENTERED OUTCOMES

YGTSS Impairment

The YGTSS Impairment [28] is a single-item measure (range 0-50), anchored by 0 (*no impairment*) and 50 (*severe impairment*). The rating focuses on distress and impairment experienced in interpersonal, academic, and occupational realms, following the presence of tics. It is administrated directly following the administration of the YGTSS symptom checklist and the YGTSS TTSS. The YGTSS Impairment can be completed in less than 5 minutes.

Clinical Global Impression Scale – Severity (CGI-S)

The CGI-S [40] is a single item clinician rating of symptom severity, in this trial used as an overall rating of the tic disorder severity. Ratings are made on a seven-graded scale from 1 ("no symptoms") to 7 ("extreme symptoms"). The CGI-S can be completed in less than a minute by an experienced rater.

Clinical Global Impression Scale – Improvement (CGI-I)

The CGI-I [40] provides a clinician-rated opinion of global improvement. The measure consists of one single item, asking about the level of improvement compared to admission, which is rated according to the following: 1=very much improved; 2=much improved; 3=minimally improved; 4=no change from baseline (the initiation of treatment); 5=minimally worse; 6=much worse; 7=very much worse". By convention, scores of "very much improved" (1) or "much improved" (2) define treatment response in clinical trials of TD [17]. The questionnaire has established validity and reliability [41]. All assessors will practice rating CGI-I together and inter-rater reliability will be calculated. The CGI-I can be completed in less than a minute by an experienced rater.

Children's Global Assessment Scale (CGAS)

The CGAS [42] is a single item 1-100 scale that integrates psychological, social, and academic functioning in children as a measure of psychiatric disturbance. It is being used in this trial to provide an assessor-rated opinion of global improvement (not restricted to only tic symptoms). The questionnaire is of compulsory use within BUP Stockholm and has established validity and reliability [43]. The CGAS can be completed in less than a minute.

Internet Intervention Patient Adherence Scale (iiPAS)

The iiPAS (paper in preparation) is an adaptation of the PEAS [44] and is designed to measure the young participant's adherence to the internet-delivered treatment. iiPAS consists of 5 items, each rated on a scale from 0 to 4, including ratings of engagement in homework, communication with the therapist and login frequency. The measure is developed by our research group, and will be rated at mid- and post-treatment. iiPAS can be completed in less than 5 minutes.

Concomitant interventions
To assess what other treatments the child/adolescent is accessing during the trial period, the family is interviewed by the assessor at post-treatment and follow-ups. This includes questions about medications (type, dose, indication and time period) and psychological treatment (type, number of sessions, indication and time period).

Blindness checks
As mentioned in section 6.2, to measure blinding integrity, all blind assessors will record whether the participating families inadvertently reveal their group allocation, and subsequently guess each participant's treatment allocation at each assessment point (post-treatment and follow-ups) and motivate their choice.

Did the participant meet the therapist?
Some, but not all, participants might meet/talk to their internet therapist face-to-face or via videoconference software (at some point) during the trial, for instance if their future therapist is the same person conducting the inclusion assessment. To be able to adjust for this potential confounder, the child/adolescent will be asked at post-treatment if s/he ever have met/talked to her/his therapist face-to-face or via videoconference software.

BIP platform usage data + therapist telephone time
Usage data will be extracted from the BIP platform and entered into the trial database. This data is collected automatically for each participant, including therapist time, number of completed chapters, number of logins, average time between logins, average pages visited, frequently visited pages, number of characters submitted, and the name of the therapist.

Telephone is not the primary way of contact during the treatment, but when used each therapist logs the time in an Excel spreadsheet. This is subsequently (at post-treatment) entered into the trial database.

Additionally, for children/adolescents allocated to BIP TIC, data is automatically collected from the *Tic Stopwatch*. This is a feature built in to the BIP TIC treatment (active group), aimed to assist the child/adolescent in practicing their tic suppression. While suppressing her/his tics, the Tic Stopwatch asks the participant every second minute to rate the intensity of their premonitory urges (sensations usually preceding tics) on a scale from 0 to 10. After each practice round, the child is also asked to rate how difficult it was to control the tics (during the previous round, on a scale from 0 to 10). The purpose of these questions is to help the participant be focused on the practice and her/his bodily sensations. The resulting data can potentially be used for future evaluation of treatment processes and predictors of treatment outcome.

5.5.4 SECONDARY CHILD-REPORTED OUTCOMES

The Child and Adolescent Gilles de la Tourette Syndrome–Quality of Life Scale (C&A-GTS-QOL)
The C&A-GTS-QOL [45] is a disease-specific measure of health-related quality of life

designed for children and adolescents with TD. Two versions exist, for the ages of 6-12 and 13-18 years respectively, with just minimal wording differences. The questionnaire consists of 27 items, each rated on a 5-point scale. In addition to the total score, C&A-GTS-QOL also consists of four subscales (psychological, physical, obsessive-compulsive, and cognitive). The questionnaire has established acceptability, validity and reliability [46]. The questionnaire takes approximately 5 minutes to complete.

Short Mood and Feeling Questionnaire – child version (SMFQ) + additional suicide item

The SMFQ [47] is a 13-item self-reported measure of depressive symptoms. Each item is scored on a 3-point scale (0 = not true, 1 = sometimes, 2 = true). The total score is derived by summing together the values for each 13 items. The questionnaire has established validity and reliability [47, 48]. Additionally, for this trial, we have also added a 14th item, aimed to assess suicide risk. The questionnaire takes approximately 5 minutes to complete.

Obsessive-compulsive inventory – child version (OCI-CV)

The OCI-CV [49] is a 21-item self-reported measure of OCD symptoms. Each item is scored on a 3-point scale (0 = never, 1 = sometimes, 2 = always). The OCI-CV generates a total score and six sub-scores (doubting/checking, obsessing, hoarding, washing, ordering and neutralizing). The questionnaire has established validity and reliability [49]. The questionnaire takes approximately 5 minutes to complete.

Treatment credibility

To assure that there are no differences between the groups in treatment credibility or expectancy of treatment outcome, we will administer a short questionnaire created by the research team, similar to the one used in our pilot trial [15]. The questionnaire consists of 3 items, asking how well the treatment suits children for managing tics, how much improvement they expect from the treatment, and how motivated they feel to work with the treatment. Each item is scored on a 5-point Likert scale, from 0 to 4. The questionnaire takes around 1 minute to complete.

Working alliance inventory – child version (WAI-C)

The WAI [50] is a 6-item scale measuring the participants perceived working alliance with their therapist. The scale is used to control for non-specific therapeutic effects between the different treatment conditions. The wording of the child version has been adjusted slightly by the research team to better suit the trial's youngest participants. The questionnaire takes less than 5 minutes to complete.

Treatment satisfaction

To assess treatment satisfaction, we use a 9-item scale created by our research team. Eight of the items are scored on a 5-point scale, asking how helpful the treatment was, if the participant would recommend the treatment to others, and more. The 9th item is a three-choice option asking whether the participant would prefer treatment at a clinic, treatment

via the internet, or has no preference. The questionnaire takes less than 5 minutes to complete.

Need for further treatment
This is a single-item questionnaire created by the research team, asking whether the participant considers her/himself in need of further treatment for her/his tics. The item is scored on a scale from 0 (no need for further treatment) to 4 (extensive need for further treatment). The questionnaire takes around 1 minute to complete.

KIDSCREEN-10 – child version

The KIDSCREEN-10 [51] is a generic paediatric quality of life measure. KIDSCREEN-10 consists of 11 items (of which 10 of the items make up an index), each with a 5-level response category. The questionnaire has shown proper psychometric properties [52]. Quality adjusted lifetime years (QALYs) will be estimated by mapping KIDSCREEN-10 scores onto Child Health Utility 9 Dimensions (CHU9D) utility weights [53]. The questionnaire takes less than 5 minutes to complete.

5.5.5 SECONDARY CAREGIVER/PARENT-REPORTED OUTCOMES

Parent Tic Questionnaire (PTQ)
The PTQ [54] assesses the number, frequency, and intensity of motor and vocal tics in young people. The questionnaire is completed by a parent, and has established validity and reliability [54]. Motor and vocal tic severity scores are computed by summing the scores for all motor and vocal tics, respectively, and a total severity score is computed by summing the two sub-scores, resulting in an ordinal scale ranging from 0 to 224 points. The questionnaire takes less than 10 minutes to complete.

Short Mood and Feeling Questionnaire – parent version (SMFQ)
See section 5.5.4 for more information. The parent version is a 13-item version without the additional suicide item.

Side effects questionnaire
Side effects (adverse events) will be recorded on a modified version of the Side effects questionnaire [55]. The scale consists of 17 short items relating to common adverse events (such as headaches, anxiety, sleep, and low mood). The caregiver/parent is asked to respond on a 5-point scale ranging from “not at all” to “all the time” to describe the presence of each item. Item 17 asks if the patient has tics, which in this trial has been modified to instead read “increased tics”, as tics will be present among all our participants. The Side effects questionnaire will be completed by the caregiver/parent with input from child/adolescent. The questionnaire takes less than 5 minutes to complete.

Treatment credibility
The caregiver/parent will answer equivalent questions as the child. See section 5.5.4 for more information.

Working alliance inventory – parent version (WAI-P)
The caregiver/parent will answer equivalent questions as the child [50]. The parent version is the original Swedish translation (compared to the child version that has been adjusted by the research team). See section 5.5.4 for more information.

Treatment satisfaction
The caregiver/parent will answer equivalent questions as the child. See section 5.5.4 for more information.

Need for further treatment
The caregiver/parent will answer an equivalent question as the child. See section 5.5.4 for more information.

KIDSCREEN-10 – parent version

See section 5.5.4 for more information.

Trimbos/iMTA Questionnaire for Costs associated with Psychiatric Illness (TiC-P)
The TiC-P [56] will be used to assess healthcare and societal resource use. The questionnaire includes items on healthcare resource use (e.g., healthcare visits), supportive resources (e.g., private tutoring), medications, prescription-free drugs, school absenteeism, academic productivity loss, and parental productivity loss and is frequently used in heath economic studies. The TiC-P version used in the study has been adapted by the research team for the use among young people and parents. The questionnaire takes from around 5 to 15 minutes to complete, depending on the family's service use.

6 PROCEDURE

6.1 RECRUITMENT

6.1.1 RECRUITMENT RATE
Recruitment is planned to be conducted within a 24-month period. Our sample size calculations show we need to recruit 220 participants. The 24-month period is in practice a 19-month period, if extracting 2.5 months each year when potential participating families are on holidays (8 weeks during summer and 2 weeks around Christmas). During these 19 months, the average recruitment rate needs to be approximately 11 participants per month. As there is often a lag period at the beginning of recruitment, before our national advertising campaign takes full effect, we anticipate a slightly slower start to recruitment. To assure we are on track with our recruitment rate, we will refer to our project milestones (see section 6.10), where

60-70 patients need to have been recruited after 12 months (approximately 7 participants per week, with holidays excluded), and 135-155 patients after 18 months (approximately 11 participants per week, with holidays excluded).

Recruitment to our pilot trial was quick (23 participants in 10 weeks), despite being limited to primarily the Stockholm area. Experience from our pilot trial indicates that participation in the trial will be attractive as it guarantees access to a behavioural intervention, which is frequently requested but often unavailable in most parts of Sweden.

An important aspect of the proposed trial is that we aim for a broad inclusion of participants to ensure adequate representation of cases with TD. All participants who are referred to our specialist clinic *BUP OCD & relaterade tillstånd* will, if eligible, be offered participation in the trial. From January 2017 to July 2018, approximately 50 patients with TD or Persistent (Chronic) Motor or Vocal Tic Disorder were assessed at the clinic. Since patients at a Stockholm specialist clinic might differ from the average TD sufferer, we will also recruit nationally from child and adolescent mental health services and paediatric neurology clinics, patient organisations, and accept self-referrals from all over Sweden. We have already an established network across several counties in Sweden, among which many clinics already have shown interest in referring participants to the trial. This broad recruitment strategy will ensure that the results are more generalizable to the entire TD population in Sweden.

6.1.2 SCREENING AND RECRUITMENT PROCEDURES

Figure 2. Flow-chart describing the steps from advertisement to randomisation.

Advertisement: Mail/e-mail information to various health care services (including child and adolescent psychiatry, paediatrics and neurology clinics) and patient organisations (e.g. Riksförbundet Attention, Svenska OCD-förbundet). Post trial information on the trial website (www.bup.se/bip) and social media. Potentially also publish paid advertisements in traditional media.

R1: Self-referral via trial website (whole Sweden):

Names + contact information is temporarily stored in our online trial database (BASS). If incomplete contact information, the participant is excluded (and immediately deleted from BASS).

R2: Referral to research clinic *BUP Klinisk forskningsenhet (KFE)* via TakeCare or regular mail (whole Sweden):

Specific referral for trial participation. Paper referrals are scanned and stored in medical record software TakeCare.

R3: Referral to specialist clinic *BUP OCD & relaterade tillstånd* via TakeCare (Stockholm only):

Ordinary referral for assessment/treatment at the clinic. Referral is stored in TakeCare.

Telephone screening:
R1 and R2: Caregiver is contacted via the telephone. Information is given about the trial. Preliminary screening of eligibility criteria. The potential participant is given a screening number. If no answer after numerous tries at different times, the participant is excluded and the reason noted (if known) in the screening log. **R3:** Caregiver is contacted via the telephone prior to coming to the clinic. The possibility of participating in the trial is presented. Information is given about the trial. Preliminary screening of eligibility criteria. The potential participant is given a screening number. This interview might also in some cases instead be performed face-to-face at the clinic.

Family interested in participation and preliminary eligible.

Family not interested in participation or not eligible.

Information sent via regular mail to the family:
The family receives trial information (informed consent form and age appropriate participant information sheet), login information to answer baseline child- and parent-reported online questionnaires (through BASS), and a pre-stamped envelope. If approached at the clinic, this information is instead handed out directly. The child/adolescent and one of the caregivers are encouraged to answer the questionnaires before attending the inclusion assessment. This gives the assessor important information before the appointment, and also introduces the family to the anticipated workload of answering questionnaires during trial participation.

Excluded from trial:
Personal information in BASS and on notes/papers are immediately deleted/destroyed. Screening number and reason for exclusion are stored for CONSORT reporting purposes.

F2F: Face-to-face inclusion assessment
VC: Videoconference inclusion assessment

All families will be able to choose between a face-to-face and a videoconference inclusion assessment, depending on their willingness to travel to the clinic in Stockholm. Both types of assessments will be identical, with only the format of delivery differing. They will include:

1. Trial information. Trial information is given, and the family can ask questions about the implication of participation.
2. Signature of informed consent form. The family will be invited to sign the informed consent form. In VC, the signatures will be shown via the web camera. If not willing to sign, the participant will be excluded and reason (if expressed) noted in the screening log.
3. Verification of information collected via telephone screening.
4. Yale Global Tic Severity Scale (YGTSS) administration: Administration of the YGTSS symptom checklist, TTSS and Impairment ratings. The administration is video recorded.
5. Diagnosing of Tourette's Disorder or Persistent (Chronic) Motor or Vocal Tic Disorder: According to DSM-5.
6. MINI-KID + OCD-RD diagnostic interviews: To assess comorbidities.
7. Collection of clinician-rated/entered demographic data.
8. Final verification of eligibility criteria.

Included in trial:
The family hands in (F2F) or is informed to mail (in the pre-stamped envelope; VC) the informed consent form, and is given a copy of it (F2F) or encouraged to photocopy/photograph it (VC). If not already completed, the family is reminded to answer the baseline questionnaires. Login information to the BIP treatment platform is given (but not yet usable). Date for treatment start is decided (at longest a week after the assessment). Name of therapist is given.

Excluded from trial:
Personal information in BASS and on notes/papers are immediately deleted/destroyed. Screening number and reason for exclusion are stored for CONSORT reporting purposes.

Randomisation:
When the informed consent form has reached the clinic (VC), and the baseline questionnaires have been answered, the participant will be given a trial ID, be randomised, and subsequently start treatment within one week.

As shown in **Figure 2**, we plan to advertise the trial to clinics across Sweden, patient organisations, as well as directly to the public via our website and social media. If needed, we might also publish paid advertisements in traditional media. Participants can be referred to the trial in three different ways: via self-referral (through the trial website), via clinic referral to our research clinic *BUP Klinisk forskningsenhet* (KFE; through medical record system TakeCare or regular mail, whole Sweden), and via clinic referral to our specialist clinic *BUP OCD & relaterade tillstånd* (through medical record system TakeCare, Stockholm only).

All referred potential participants will be initially contacted via the telephone by a member of the research team (*Telephone screening*), or in some cases approached face-to-face at the clinic. The main purpose of this contact is to perform a preliminary screening of eligibility, which includes asking brief questions about current and previous tics, and each inclusion/exclusion criteria (see section 5.4 for full information). Some criteria, like the YGTSS TTSS cut-off score, will instead be assessed during the next step (*Inclusion assessment*). The researcher will also inform briefly about the trial and outline the time commitment associated with participation, as well as answer any questions. The telephone interviews are by default done with the caregiver, but the child/adolescent could in some cases provide additional information.

If preliminarily eligible and interested, the participants will be booked for an inclusion assessment, either face-to-face at the clinic or via videoconference software Zoom. Before the assessment, the potential participants will be provided with information (via regular mail, or handed out at the clinic), including the informed consent form, an age appropriate participant information sheet, and login information to answer baseline child- and parent-reported online questionnaires. This will provide the research team with important information already before the inclusion assessment, giving the opportunity to further assess potential risks (for instance high scores on the depression measures SMFQ), to promote participant safety. It will also give the participants an opportunity to experience the data collection work load of the trial, making it possible for them to decline their participation at an early stage.

The participant is encouraged to sign the informed consent form prior to (or in the very beginning of) the inclusion assessment, which will be verified by the assessor (in videoconference assessments the signatures will be shown via the web camera). In addition to the core question about trial participation, the child/adolescent and all legal caregivers are also invited to consent that the video recordings of the YGTSS interviews are kept beyond the end of the trial for internal (with the research team) education and training purposes. The latter will be optional and will not affect participation in the trial.

If eventually included in the trial, the participant will be instructed to mail the consent form to the research team (in the already received pre-stamped envelope). In the unlikely event that new safety information results in significant changes to the risk/benefit assessment, the consent form will be reviewed and updated, along with an amendment sent to the Regional

Ethical Review Board in Stockholm, and if necessary, participants will be re-consented as appropriate.

The inclusion assessment will be conducted by a member of the research team (under supervision of a clinical expert). Where possible, this will be the same person who conducted the telephone screening. The assessor will explain the aim, methods, benefits and hazards of participating. Further, s/he will explain that the participants are under no obligation to enter the trial and that they can withdraw at any time, without having to give a reason. See **Figure 2** for complete information about what the assessment includes. If all inclusion criteria, and no exclusion criteria, are met, the participant will be included in the trial. No deviations or waivers to the eligibility criteria are allowed. If the assessor is uncertain whether a potential participant is eligible, the PI will be informed and have the final decision. Patients who fail screening because they have recently altered their tic medication (within 8 weeks), or received behaviour therapy within the specified exclusion time frame (12 months), may be eligible for re-screening at a later date when these time exclusions have passed. If possible, participants included following a face-to-face inclusion assessment will be offered to briefly greet their future therapist face-to-face, and this will be recorded.

When the consent form has reached the research team (and is approved to be correctly signed), and all baseline measures have been answered, the participant will be assigned a trial ID and be randomised. The signed form will be retained in a locked file cabinet. A log will be kept matching screening IDs with trial IDs. The participants will be invited to choose which day to start treatment, as long as they start within one week. Even if the participant has not logged in to the platform within one week, the treatment is considered as started, and the chosen date remains.

If the participant is not included in the trial (regardless of reason), but still requires clinical attention, our team will initiate a referral to other suitable services, whenever possible.

6.1.3 CHANGES TO PROTOCOL DUE TO THE COVID-19 PANDEMIC

Due to the COVID-19 pandemic, minimal adaptations to the assessment and consent procedures were implemented from March 2020 (that is, from study participant 082), as follows:

- To minimise the risk of infection, participants may no longer choose between face-to-face assessments at our clinic or video conference assessments. Instead, all assessments are performed via video conference software.
- To avoid delays, participants may be included and randomised immediately after showing the signed informed consent form to the researcher via the web camera during the inclusion assessment (i.e. before the informed consent form has reached the clinic via regular mail).

The ethics review authority will be made aware of these minor changes to the protocol, which are likely to remain in place until the end of the study.

6.2 RANDOMISATION, ENROLMENT AND MASKING

Participants will be randomised at a 1:1 ratio to BIP TIC (therapist- and parent-guided internet-delivered behaviour therapy) and a control condition (therapist- and parent-guided internet-delivered education on tics). Randomisation will be conducted by one or several researchers (according to a task delegation list) using an online randomisation service (Randomize.net; set up and monitored by the Karolinska Trial Alliance [KTA; karolinskatrialalliance.se/en). Randomly varying block sizes will be generated using a computer random number generator. One or several researchers (according to a task delegation list) will be responsible for the enrolment of participants and assigning participants to therapists. Participants will be informed that they will be allocated to one of two behavioural interventions for TD/Persistent (Chronic) Motor or Vocal Tic Disorder.

Assessors conducting post- and follow-up assessments will be blind to treatment allocation, for the full duration of the trial. The outcome measures are identical for both groups, ensuring that the assessors remain blind. At each follow-up assessment, participants will be reminded by their assessor to not reveal their arm allocation. To measure blinding integrity, all assessors will record whether the participating families inadvertently reveal their group allocation, and subsequently guess each participant's treatment allocation at each assessment point and motivate their choice. If the treatment allocation is accidently revealed, that very part will be cut out of the video recording. A new blind assessor will then watch the video recording and conduct the rating that will be used in the trial. Subsequent assessments for that participant will then be conducted by a different assessor (than the first one; blind to treatment allocation), where possible. The primary analysis will be performed after the trial's final participant has finished his/her 3-month follow-up assessment (primary endpoint). Assessors will remain blind for the 6- and 12-month naturalistic follow-up assessments. The trial will end when the trial's final participant has finished his/her 12-month follow-up assessment.

In case of a medical emergency, participants will be encouraged to immediately seek appropriate health care, and to inform their therapist about this, who in turn will inform the trial coordinator. The trial coordinator will be in charge of following up the incident. This whole procedure can be done without unblinding the outcome assessors, so an emergency unblinding system is not required for the trial.

6.3 ASSESSMENT POINTS FOR EACH OUTCOME

Table 1. Assessment points for each outcome.

Outcome \ Assessment point	Screening	Baseline	Mid-treatment	Post-treatment	3-month follow-up (**primary endpoint**)	6-month follow-up	12-month follow-up
**Screening/Baseline measures**							
Self-referral questionnaire (child- or parent-reported)	X (self-referred participants only)						
Demographic data (clinician-entered)		X					
Demographic data (parent-reported)		X					
MINI-KID (clinician-administered)		X					
OCD-RD (clinician-administered)		X					
AQ-10 (parent-reported)		X					
SNAP-IV (parent-reported)		X					
PUTS (child-reported)		X					
**Clinician-rated/entered outcomes**							
YGTSS symptom checklist		X		X	X	X	X
YGTSS TTSS (**primary outcome measure**)		X		X	X	X	X
YGTSS Impairment		X		X	X	X	X
CGI-S		X		X	X	X	X
CGI-I				X	X	X	X
CGAS		X		X	X	X	X
iiPAS			After 5 weeks	X			
Concomitant interventions				X	X	X	X
Blindness checks				X	X	X	X
Did the participant meet the therapist?				X			
BIP platform usage data + therapist telephone time				X			X (Number of logins only)
Assessment format (face-to-face or video conference)		X		X	X	X	X
**Child-reported outcomes**							

C&A GTS-QOL 6-12 or 13-18, depending on the participant's age		X		X	X	X	X
SMFQ Child + additional suicide item		X	After 5 weeks	X	X	X	X
OCI-CV		X		X	X	X	X
Treatment credibility			After 3 weeks				
WAI-C			After 3 weeks				
Treatment satisfaction					X		
Need for further treatment					X		
KIDSCREEN-10 child		X		X	X	X	X
**Caregiver/parent-reported**							
PTQ		X	After 5 weeks	X	X	X	X
SMFQ Parent		X	After 5 weeks	X	X	X	X
Side effects questionnaire		X	After 5 weeks	X	X		
Treatment credibility			After 3 weeks				
WAI-P			After 3 weeks				
Treatment satisfaction					X		
Need for further treatment					X		
KIDSCREEN-10 parent		X		X	X	X	X
TiC-P		X		X	X	X	X

Note: For full names of abbreviations, see section 5.5.

6.4 BASELINE ASSESSMENT

See section 6.1.2 and **Table 1** for complete information about the baseline assessment.

6.5 TREATMENT

Both treatments are internet-delivered, using the BIP platform. This platform is previously developed by our research team, and is currently used in several trials. BIP is specifically designed for use by children and adolescents, and their parents, with age-appropriate appearance, animations and interactive scripts (www.bup.se/bip).

Each intervention consists of 10 chapters (modules), delivered over 10 weeks. During this time, participants (both the child/adolescent and the caregiver/parent) have regular contact with a trained therapist inside the platform. The therapist provides feedback, answer questions, and reminds to complete the next module if required. Communication is possible via traditional text messages (resembling e-mail) or via comments on specific worksheets. When the participant receives a message in the platform, s/he also gets an SMS reminder to her/his phone (in some cases only sent to the parent). The therapist usually logs in daily (on

workdays; at minimum every 48 hours). Therapist time is automatically logged in the platform. There are no specified limits to the therapist time, however, in our pilot trial [15], the average therapist time per participant per week was 25 minutes. Participants are typically in contact with their therapist several times a week. Phone calls are possible if the participant/therapist for a certain reason feels it is needed, but are generally kept to a minimum. The participants are welcome to contact their therapist at any time, who will reply during office hours. Telephone time is manually logged in an Excel spreadsheet by the therapist, and later entered to the trial database.

The therapist is available for 10 weeks. In certain circumstances (e.g. illness or holidays), participants can pause their therapist-support for one or two weeks, which extends the treatment length to a maximum of 12 weeks. The participants can work through the first three modules as quickly as they prefer. For the following modules, the quickest possible pace is one chapter per week. After 9 weeks, all modules will be made available for the participant (regardless of how many modules s/he has completed). After the 10-12 weeks, the child/adolescent and caregiver/parent can continue to access all treatment modules for the whole follow-up period (12 months), but without therapist-support.

For both treatments, treatment completion is defined as completion of the first four child chapters, which contain the minimum amount of information required for the patients to understand the treatment rationale and the tasks that they are supposed to carry out.

6.5.1 BIP TIC

The active treatment condition is called BIP TIC. The treatment manual (constructed by our research team) consists of evidence-based interventions adapted from previously published treatment manuals on exposure and response prevention (ERP) [57], with some components (functional analysis and interventions) also originating from the CBIT-protocol [58]. Each of the 10 modules includes age-appropriate texts, animations and exercises. The intervention is primarily based on ERP techniques. During the treatment, participants are instructed to practice suppressing their tics, this is known as ‘response prevention’. Then, with the help of their caregiver/parent, the participant is instructed to provoke premonitory urges (a sensation usually felt before a tic is expressed), while still suppressing tics, which is known as ‘exposure’.

The child/adolescent and the caregiver/parent are provided with their own separate login to the BIP platform. The parent login consists mainly of information regarding parent coping strategies, social support and functional analysis relating to tics. Both the child and the caregiver have individual access to the same therapist. Caregivers could have up to two parallel logins to the caregiver treatment, if for instance living separately.

The content of the modules is as follows:

1. *Child*: Learn about tics.
2. *Child*: Preparation of practice.
3. *Child*: Practicing stopping your tics (response prevention).
4. *Child*: Making the practice more challenging (exposure and response prevention).
5. *Child*: Continued practice.
6. *Child*: School.
7. *Child*: Talking about your tics.
8. *Child*: Continued practice.
9. *Child*: The final sprint.
10. *Child*: Plan for the future.

1. *Parent*: Introduction.
2. *Parent*: Thoughts, feelings and behaviours of parents.
3. *Parent*: Praise.
4. *Parent*: Prompts (remind child/adolescent to use treatment strategies).
5. *Parent*: Situations and reactions (functional analysis and interventions).
6. *Parent*: Troubleshooting.
7. *Parent*: Continued practice.
8. *Parent*: Continued practice.
9. *Parent*: Continued practice.
10. *Parent*: Plan for the future.

6.5.2 ACTIVE CONTROL CONDITION

The active control treatment condition (comparator) consists mainly of psychoeducational information about TD and common comorbid conditions. The treatment is designed to match BIP TIC in all aspects except for the module content (same platform, same treatment length, same therapist-support etc.). The modules are approximately matched in length, but differ in content.

The control condition reviews the definition of tics, natural history, common presentations, prevalence, aetiology, risks and protective factors, strategies for describing tics to other people, among others. Problem-solving and development of expertise in tic disorders is emphasised. The active intervention includes strategies for promoting positive behaviours which will be rewarded by a parent as a parallel element to the ERP practice in the BIP TIC arm. The control condition will not include any information on ERP or functional analysis and interventions. The control condition is an adapted version of the psychoeducational intervention used in the large, multi-centre, CBIT-trial [17].

The content of the modules is as follows:

1. *Child*: Introduction.
2. *Child*: Tics and tic list.
3. *Child*: Become and expert in tics.

4. *Child*: Other conditions (common comorbid conditions).
5. *Child*: Healthy habits.
6. *Child*: School.
7. *Child*: Tell others about your tics
8. *Child*: Risk and protective factors.
9. *Child*: Tics and the future.
10. *Child*: Plan for the future.

1. *Parent*: Introduction.
2. *Parent*: Praise.
3. *Parent*: Prompts (remind child/adolescent to use treatment strategies).
4. *Parent*: Other conditions (common comorbid conditions).
5. *Parent*: Healthy habits.
6. *Parent*: School.
7. *Parent*: Thoughts and feelings of parents.
8. *Parent*: Risk and protective factors.
9. *Parent*: Looking after yourself.
10. *Parent*: Plan for the future.

6.5.3 THERAPIST TRAINING, SUPERVISION AND MONITORING

All therapists will receive training before the trial starts. They will first have a seminar on the use of the BIP platform. Second, they will read the TD manual by Verdellen et al. [57] to familiarise themselves with the principles of ERP, as well as the therapist manuals developed for the trial. Third, they will practise to deliver BIP TIC, and receive feedback from a TD expert who will act as a 'dummy participant'. Fourth, therapists will also practise to deliver the comparator treatment (with TD experts as 'dummy participants'), with explicit instructions not to provide any advice on the use of key behavioural components (ERP).

During the trial, TD experts (supervisors) will regularly log in to the system and monitor the communication between patients and therapists, and provide feedback to therapists, to ensure adherence to protocol. Regular supervision of the text communication between patients and therapists is critical for quality control and adherence to protocol (e.g., to ensure that therapists delivering the comparator do not inadvertently leak information from BIP TIC). Protocol violations will be recorded.

Regular meetings ("ward rounds") within the research team will be held to discuss treatment progress for individual patients, and follow up on clinical needs and potential adverse events.

6.6 SUBSEQUENT ASSESSMENTS

6.6.1 MID-TREATMENT ASSESSMENTS

Data is collected at two different time points mid-treatment: 3 and 5 weeks into treatment. See **Table 1** for information regarding which measures are administered at each time point. There is a +14 days time period for data collection.

6.6.2 POST-TREATMENT ASSESSMENT

The post-treatment assessment is conducted face-to-face at the clinic or via videoconference software (depending on the participant's preference). The YGTSS interview will be video recorded. If technical problems with the videoconference software Zoom, the assessment will be made over telephone instead (with the family on speaker telephone). Child- and parent-reported questionnaires are answered online directly to our trial database BASS, preferably prior to the face-to-face/videoconference assessment. See **Table 1** for information regarding which measures are administered at this time point. The post-treatment assessment is planned to occur as quickly as possible after the end of the 10-week treatment, and never later than 2 months after the treatment.

6.6.3 3, 6 AND 12-MONTH FOLLOW-UPS

Follow-ups will be administered 3, 6 and 12 months after the end of the 10-week treatment. As with the post-treatment assessment, these assessments are conducted face-to-face at the clinic or via videoconference software (depending on the participant's preference). The YGTSS interview will be video recorded. If technical problems with the videoconference software Zoom, the assessment will be made over telephone instead (with the family on speaker telephone). Child- and parent-reported questionnaires are answered online directly to our trial database BASS, preferably prior to the face-to-face/videoconference assessment. See **Table 1** for information regarding which measures are administered at each time point. For these follow-ups, there is a -1 month/+2 month time period for data collection.

6.7 END OF TRIAL

The trial will end when the final data (at the 12-month follow-up) has been collected for the final participant.

6.8 PARTICIPANT WITHDRAWAL FROM TRIAL

Participants are free to withdraw from the trial at any point. If participants indicate to the research team/therapist that they would like to withdraw from the trial, they will be asked if previously collected data can be kept for trial analysis, if not, all data will immediately be deleted (except for screening number). After the withdrawal, participants will not be requested to complete any further measures, but will be asked to provide non-obligatory feedback regarding their reason for withdrawal. This reason (if given) will be logged for CONSORT reporting purposes. Once participants have withdrawn from the trial, it will not be possible to re-enter or resume treatment. Withdrawn participants will not be replaced in the trial.

Participants will not be withdrawn from the trial if they are inactive in treatment. If the child states that s/he do not wish to continue her/his treatment, the parent will still be encouraged to continue working with her/his separate modules. In these cases, participants will still be invited to the follow-up assessments (following intention-to-treat principles). Participants will be informed that it is helpful for the trial to collect outcome measures, even if they did not complete their treatment. In cases where the participants clearly state that they do not want to complete any more follow-up measures, or withdraw from the trial entirely, this will be followed.

Failure to complete outcome measures at one follow-up time point will not imply withdrawal from the trial, and the participants will still be invited to complete measures at the next follow-up time point, unless they explicitly state otherwise.

6.9 DISCONTINUATION OF TRIAL

Outcome measures will not be analysed until the end of the trial period (more specifically from the point that the last participant reaches the primary endpoint) and will therefore not inform decisions to stop the research. However, serious adverse events (see section 9.1 for definitions) will be reviewed, and if there is any indication that these are linked to the intervention consideration will be given to discontinuing the trial on the advice of the Karolinska Trial Alliance (KTA) and trial Sponsor.

6.10 PROJECT MILESTONES

Specific intermediate objectives will be monitored in collaboration with the Karolinska Trial Alliance (KTA), the Sponsor and our funders. The criteria have been decided based on the experiences from our pilot trial [15]. If these criteria are not met, we will re-evaluate our trial procedures.

1) The trial needs to have recruited 60-70 patients by the end of the first year of recruitment. 18 months after the start of the project, we should have recruited 60-70% of the required sample (135-155 patients).
2) A minimum of 80% of participants need to have completed at least the first 4 chapters (which contain the main active ingredients of the treatments).
3) At least 80% of the randomised participants need to have completed the primary outcome measure at the primary endpoint.
4) Our independent monitors (KTA) are satisfied with progress and data quality.

6.11 CONCOMITANT INTERVENTIONS

Participants are free to continue any medication during the trial, which for example enables continuation of medication for ADHD, which is very common within this patient group. Medication for the indication of tics (primarily antipsychotics and α_2-agonists) is required to have been stable (type of drug and dosage) for the last eight weeks prior to inclusion in the

trial. Additionally, the participant is encouraged to (if possible according to her/his treating clinician), not to alter her/his tic medication until after the 3-month follow-up.

Participants must not have received previous behaviour therapy for tics, for a minimum of eight sessions with a qualified therapist, within the 12 months prior to inclusion into the trial. Further, the participants are asked to not start any parallel/new psychological treatment (of any type) for the indication of tics until after the 3-month follow-up.

As stated in section 5.1, the post-treatment and 3-month follow-up are "intention-to-treat" assessment points, where participants are encouraged not to change medication or start alternative psychological treatment (for their tics). The 6- and 12-month follow-ups are naturalistic follow-ups, where participants may be using alternative treatments to a higher extent, in accordance to standard practice recommended by their usual treating clinician.

Data on use of medication and psychological interventions are collected at each assessment point. Any deviations to protocol will be noted and potentially considered in the statistical analysis.

6.12 QUALITY CONTROL

The trial will be conducted according to good clinical practice principles (GCP). Data quality and safety aspects will be regularly monitored by an independent party, the Karolinska Trial Alliance (KTA). Statistical analyses will be conducted under the guidance of the Karolinska Institutet Biostatistics Core Facility (www.biostatcore.ki.se), as well as under guidance by Dr Inna Feldman and Dr Filipa Sampaio at Uppsala University (regarding cost-effectiveness) (see section 8 for more information).

7 DATA MANAGEMENT

All aspects of data management of the trial will comply with the General Data Protection Regulation (GDPR) and good clinical practice (GCP).

Notes will be made in the medical record software TakeCare (included participants only) according to BUP Stockholm standard routines for internet-delivered treatment (in research trials).

7.1 DATA COLLECTION AND HANDLING

Data will be collected both manually (paper Case Report Forms [CRFs]) and digitally (child- and parent-reported questionnaires completed via the internet directly into the trial database [BASS]). Some data will also be extracted digitally from the treatment platform BIP, and then entered to BASS (see "BIP platform usage data" in section 5.5.3).

The CRFs will not bear the participant's name, instead the trial identification number will be used for identification. CRFs will be stored securely in a locked file cabinet. Data from the

CRFs will consecutively be entered manually into BASS by a member of the research team. The accuracy of the data entry for the primary outcome measure (YGTSS TTSS) will be checked by the Karolinska Trial Alliance (KTA). The delegation log will identify all those personnel with responsibilities for data collection and handling, including those who have access to the trial database.

Video recordings will be stored on a secure server (VPN). The recordings will be used for spot methodology checks and future training of trial assessors.

7.1.1 BIP PLATFORM

BIP is the IT platform that delivers the treatments. BIP may contain personal sensitive information such as text messages between the therapist and child/adolescent or caregiver/parent. BIP has a fine-grained privilege-system which ensures that personnel (e.g. therapists) only can view information and edit settings that pertain to their role in the research project (e.g. only view their personally assigned participants). All data sent between the BIP platform and the user are encrypted using a 2048-bit SSL certificate. The system is set up so that only a port 80 and 443 is visible on the internet to the public. A firewall called ModSecurity is also installed on the server where different rules can be activated. The server has rules activated to prevent attacks with SQL injection and brute force. The server will also be continuously updated by the server-company (GleSYS) that installs security-patches when they are released, as another measure to prevent hackers (or similar).

7.1.2 BASS

BASS is the name of the trial online database. BASS is used both for manual entry of clinician assessed/gathered information, as well as for remote (via the internet) administration of child- and parent-reported questionnaires. Regardless of the method above, all data goes into the same database. The participants will login directly to BASS at baseline, but will for all following assessment points instead be directly forwarded to BASS when logging in to BIP (when time to answer a questionnaire). When the questionnaire has been completed, the participant is automatically forwarded back to BIP. BASS has a fine-grained privilege-system which ensures that personnel only can view information and edit settings that pertain to their role in the research project, including only viewing their relevant participants. All data sent between the platforms and the user are encrypted using a 2048-bit SSL certificate. The system is setup so that only a port 80 and 443 is visible on the internet to the public.

Both BIP and BASS systems store names of participants and telephone numbers (for the use of sending SMS). BASS also store e-mail addresses (for the use of sending links to self- and parent-rated questionnaires). Logging into the BIP and BASS platform requires username, password, and a temporary code sent by SMS (i.e. two-factor authentication which is required for systems storing sensitive data). Once the trial is finished, all personal information (names and telephone numbers) will be removed from both systems.

When the trial has ended, data will be extracted from BASS by the trial coordinator (Andrén), then properly prepared/cleaned (by a member of the research team, including adding a variable for the treatment allocation), and then securely transferred to the parts responsible for statistical analyses (see section 8). Some analyses will also be made locally by the research team.

7.1.3 ZOOM

Zoom is the software solution that will be used for the videoconference assessments. Zoom can be used in the common modern browsers (e.g. Chrome, Safari and Firefox), as well as an external application. Through Zoom, an e-mail is sent from the research team with a link from where the participant initiate the video conference. All video conference calls will be end-to-end encrypted using the encryption standard AES-256 for maximum available data security. The AES is included in the ISO/IEC 18033-3 standard on Security techniques for information technology.

8 STATISTICAL ANALYSIS

Statistical analyses will be conducted under guidance of the Biostatistics Core Facility at Karolinska Institutet (clinical efficacy and 12-month durability; www.biostatcore.ki.se) and the Department of Public Health and Caring Sciences at Uppsala University (health economics). Full detail on the planned statistical analyses and the health economic evaluation can be found in the Appendix, the BIP TIC RCT - Statistical and health economic analysis plan (BIP TIC RCT SAP, current version 1.0, 2021-02-03). In the possible case that any information regarding statistical analyses or the health economic evaluation presented in this protocol would differ from the BIP TIC RCT SAP, the BIP TIC RCT SAP is considered the original source to proceed from.

8.1 PRIMARY OBJECTIVE: CLINICAL EFFICACY OF BIP TIC

Summary of baseline data:

We will follow the CONSORT guidelines in reporting our data. The demographic information collected at baseline will be summarised and presented in a table, by randomisation arm. Categorical variables will be reported as counts and percentages. Continuous variables will be summarised as means, medians, and interquartile ranges. According to CONSORT recommendations, no statistical tests will be performed to assess baseline differences between study arms [59].

Primary outcome analysis plan:

The principal analyses will follow a pre-specified intention-to-treat statistical analysis plan (the BIP TIC RCT SAP), which will be finalised prior to unblinding. The primary outcome variable is the YGTSS Total Tic Severity Score, at the primary endpoint (3-month follow-up). As this is a composite measure resulting in an ordinal variable, statistical modelling will

employ medians rather than means. Specifically, all the randomised participants will be included to estimate a linear quantile mixed model [29-31], taking into account individual differences in pre-treatment symptomatic status, and treatment response. The model will include fixed effects of time (baseline, post-treatment, and 3-month follow-up) and subject effects as a random intercept factor to account for the variances between and within participants. Linear quantile mixed models use all available data, can properly account for correlation between repeated measurements on the same subject, have greater flexibility to model time effects, and can handle missing data.

Additionally, to enable comparisons with previous trials within the field, which traditionally have used regression models based on means rather than medians, we will also perform complementary analyses of the primary outcome measure at the primary endpoint using a linear mixed model (estimating a difference in means).

The procedures for handling missing data are described in detail in the BIP TIC RCT SAP.

Analysis of secondary outcomes:

Secondary outcomes will be analysed using similar statistical methods as the primary outcome. Ordinal variables resulting from the various questionnaires will be analysed with linear quantile mixed-models, which will be complemented with linear mixed models to facilitate comparison with previous trials Dichotomous outcomes will be analysed using logistic regression. The results will be presented as estimates or odds ratios, as appropriate, for the regression coefficients with their respective 95% confidence intervals and *p*-values. We will also report numbers needed to treat, based on the proportions of responders in each group (scores of "very much improved" [1] or "much improved" [2] on the CGI-I [41]). When many tests may be carried out, we will interpret the results in the context of multiple testing.

8.2 SECONDARY OBJECTIVE #1: 12-MONTH DURABILITY OF THE TREATMENT EFFECTS

All trial participants will be naturalistically followed up and assessed 6 and 12 months after the end of the 10-week treatment. Linear quantile mixed-effects models will be implemented for the primary and secondary outcome measures. As in the primary objective, for each outcome measure, the model will include fixed effects of time (5 time points) and subject effects as a random intercept factor to account for the variances between and within participants. In addition, we will perform complementary linear mixed models (estimating a difference in means) for each of the linear quantile mixed models performed. The linear mixed models will be performed to enable comparisons with previous trials in the field.

8.3 SECONDARY OBJECTIVE #2: HEALTH ECONOMIC EVALUATION OF BIP TIC

For the reporting of the health economic evaluation, we will follow the Consolidated Health Economic Evaluation Reporting Standards (CHEERS) guidelines. The short-term health economic evaluation will compare BIP TIC and the control condition at the primary endpoint

(3-month follow-up) and the results will be published together with the efficacy results of the trial (primary objective). As a long-term evaluation, we will then perform the same analyses at the end of the follow-up period (12-month follow-up) using cumulative information collected throughout the follow-up. These results will be published together with the results of the naturalistic follow-up (first secondary objective).

The health economic evaluation will be performed from three different perspectives, with gradually increasing costs included for each perspective:

1. The first perspective is the health organisation payer perspective, which includes direct treatment costs, of BIP TIC and the comparator, for the clinic. This comprises personnel costs such as therapist time, administration time, IT platform maintenance, and other overheads.
2. The second perspective will additionally include other healthcare resource use outside the clinic for the children, such as costs for medical appointments (outside the trial) and medications.
3. The third perspective will further comprise other societal costs, including productivity losses for the children related to absenteeism and presenteeism from school and leisure activities, and productivity losses for the parents due to absenteeism from work.

The Trimbos/iMTA Questionnaire for Costs associated with Psychiatric Illness (TiC-P [56]) will be administered (in an adapted version for young people and parents) at each assessment point (except 3 and 5 weeks into the treatment) to collect information on frequencies of resource use for the children and absenteeism from work for the parents. Resource use costs will be estimated by multiplying frequencies by national Swedish tariffs and market prices. Total costs for each group will be aggregated over the trial period.

For each of the three perspectives above, we will conduct two types of analyses:

1. A cost-effectiveness analysis, using responder rate as the outcome [60].
2. A cost-utility analysis using Quality Adjusted Life Years (QALYs) as the outcome [60].

Health gains in terms of QALYs will be estimated by mapping KIDSCREEN-10 scores [51] onto Child Health Utility 9 Dimensions (CHU9D) utility weights [53]. Total QALY gains over the trial period will be estimated by using the area under the curve method [61]. Differences in QALYs and costs between both trial arms will be investigated using generalised linear models with suitable distributions [62].

To ascertain whether BIP TIC is cost-effective, relative to the comparator, incremental cost-effectiveness ratios expressed as cost per additional responder and cost per additional QALY will be presented. The uncertainty around the cost and effect estimates will be presented using a cost-effectiveness acceptability curve [60].

8.4 INTERIM ANALYSIS

There are no interim analyses planned.

8.5 PREDICTOR AND MODERATOR ANALYSIS

Post-hoc analyses may be conducted investigating predictors and moderators of treatment response. More specifically, the following variables can be hypothesised to potentially predict or moderate treatment outcomes: a) tic severity (measured by the YGTSS TTSS [28]), b) degree of premonitory urge (measured by the PUTS [38]), c) OCD symptoms (measured by the OCI-CV [49]), d) ADHD symptoms (measured by the SNAP-IV [35]), e) autism symptoms (measured by the AQ-10 [34]), and f) medication status [63]. Additionally, we predict that adherence during treatment (measured by the iiPAS, therapist time, number of logins and sent messages) and also motivation to complete the treatment (measured using the Treatment credibility scale, item 3, at week 3) will significantly predict or moderate treatment outcomes.

9 RECORDING AND REPORTING OF ADVERSE EVENTS AND REACTIONS

9.1 DEFINITIONS

Table 2. Definitions of serious adverse events.

Term	Definition
Serious Adverse Event (SAE)	Any untoward occurrence that:<br>• results in death<br>• is life-threatening<br>• requires hospitalisation or prolongation of existing hospitalisation<br>• results in persistent or significant disability or incapacity<br>• consists of a congenital anomaly or birth defect<br>• is otherwise considered medically significant by the investigator
Suspected Unexpected Serious Adverse Reaction (SUSAR)	Any SAE that is deemed to be<br>• Related to the trial intervention<br>AND<br>• Unexpected (not listed in the protocol as an expected adverse event of the intervention)

9.2 EXPECTED ADVERSE EVENTS

The following adverse events were noted for BIP TIC in our pilot trial [15], and are considered as expected in the proposed trial. A majority of the reported adverse events subsided during the second half of the treatment, indicating that they were temporary.

- Increased tics
- Increased anger/outbursts/disruptive behaviour
- Increased irritability
- Increased anxiety/stress
- Increased depressed mood
- Increased tiredness/fatigue
- Increased/decreased sleep
- Headaches

It is important to note that these adverse events may also be symptoms of the underlying condition (for instance natural waxing and waning of tics), rather than the intervention itself. It is further likely that participants will be receiving different types of medications during the trial. Adverse events will be closely monitored by the therapist and reported in accordance to the procedures outlined in this protocol.

9.3 ASSESSMENT OF ADVERSE EVENTS

A. RELATED EVENTS

The assessment of the relationship between adverse events and the administration of the treatment is a decision based on all available information. The final decision is taken by the PI. If the event is a result of the administration of any of the research procedures then it will be classed as related. The classification of adverse events as related or unrelated will primarily be used for SAEs.

B. EXPECTED EVENTS

If the event has been listed in the protocol (section 9.2) as an expected side effect of the intervention then the event will be classed as expected. If the event is not listed then it will be classed as unexpected.

9.4 HANDLING OF ADVERSE EVENTS

All adverse events will be noted by the trial coordinator in a specific log (including date, recorded clinical symptoms, and a brief description of the event), which in turn will be shared with the Karolinska Trial Alliance (KTA). The research team could be notified about adverse events in numerous ways, including through direct (text or telephone) communication with the participant, and completions of the Side effects questionnaire. Regular meetings ("ward rounds") will be held within the research team where treatment progress and potential adverse events are discussed. High scores on SMFQ (depressive symptoms) including its additional suicide item, might also be indicators of adverse events. Scores above 13 points, or

scores of 2 or 3 points on the suicide item, will automatically raise a flag in the BASS system, to directly notify members of the research team to follow this up (via the telephone) with the participant.

SAEs and SUSARs will be recorded in the patient's medical record (Take Care), in ELN, as well as in the trial coordinator's log. Appropriate action will be taken in the case of SAE and SUSAR, making sure the participant will get in contact with suitable health care services. Events will be considered as potentially treatment-related up to the 3-month follow-up, where the reporting of adverse events will terminate.

9.5 REPORTING OF ADVERSE EVENTS

As mentioned above, AEs and SAEs may be reported through various sources. Here follows a more detailed description of how the various sources will be combined when reporting AEs and SAEs in the resulting publications:

1) AEs/SAEs may be reported to the therapist or the researcher through communication with the participants.
2) AEs are also recorded through completion of the SMFQ. SMFQ data collected at baseline, 5 weeks into treatment, post-treatment and the 3-month follow-up will be used for AE reporting purposes. Any total score >13 or score >1 at the additional suicide item, at 5 weeks into treatment, post-treatment or the 3-month follow-up, is recorded as an AE **if** it is greater than their baseline score. Only the child version of the SMFQ will be used for AE reporting.
3) AEs are also recorded through the Side effects questionnaire completed at baseline, 5 weeks into treatment, post-treatment and the 3-month follow-up. A score on any item that is equal-to-or-greater-than 2 ("about half the time") **and** greater than their baseline score is recorded as an AE. The Side effects questionnaire is completed by the parent.

The listed sources above will be combined and AEs will be summarized by number (frequencies and percentages), nature, severity and whether they are expected or unexpected. If there are any SAEs, the trial coordinator and the PI will judge whether these are related to the treatments provided. No statistical tests comparing the treatment groups will be performed.

9.6 NOTIFICATION OF SERIOUS BREACHES TO GCP AND/OR THE PROTOCOL

For the purpose of this protocol we define a "serious breach" as a breach which is likely to a significant degree affect:

(a) the safety or physical or mental integrity of the participants of the trial.

OR

(b) the scientific value of the trial.

The PI will immediately notify the Sponsor of any case where the above definition applies during the trial. The PI and Sponsor will work together to identify the extent of the breach and to determine what urgent safety measures are required. The PI and Sponsor will devise a formal plan of corrective action to address the breach.

The Sponsor of the trial will notify the Regional Ethical Review Board in Stockholm in writing of any serious breach of (a) the conditions and principles of GCP in connection with that trial; or (b) the protocol relating to that trial, as amended from time to time. Reports of serious breaches will give details of when the breach occurred, the location, who was involved, the outcome and any information given to participants. An explanation regarding the cause of the serious breach will be given, and the Ethical Review Board will be informed what further action the Sponsor plans to take.

10 PUBLIC AND PATIENT INVOLVEMENT

Involving people with experience of TD is vital to develop an intervention that young people will want to use, and to design an evaluation inclusive of the perspectives of patients and parents. Prior to the development of BIP TIC, a focus group was convened in Stockholm, including five children with TD and their parents. From this, we learnt that young people and their parents are enthusiastic about digital interventions for tics that allow them to manage tic-related distress during daily life. They liked being able to rate their tics and detect patterns. They particularly liked the idea of having access to help and information on their own device at a time they chose in addition to remote therapist support. This feedback informed the initial development of BIP TIC.

At the end of our pilot trial [15], involving 23 children and their parents, we gathered extensive user experience data and conducted detailed qualitative interviews with the families. Participants evaluated the intervention as highly acceptable, safe, and helpful in reducing tics. Satisfaction ratings were very high. By the end of the treatment, 83% of both the children and the parents rated BIP TIC as being *good* or *very good*. Fifty-seven percent of the children and 48% of all parents reported that BIP TIC had helped them *much* or *very much* with their tics, and their child's tics, respectively. Eighty-seven percent of the children and 87% of the parents said it was *likely* or *very likely* that they would recommend this treatment to a friend. Only 4% of the children and 30% of the parents reported that they would have preferred face-to-face treatment instead of the internet-delivered intervention.

This positive feedback from patients and their parents, together with feedback from treating therapists, and from Elisabeth Pousette, a TD-sufferer and former Head of the Svenska OCD-förbundet, encouraged us to further develop BIP TIC and plan the current trial.

Specific changes made to BIP TIC based on patient feedback included: 1) reduction of the overall amount of text; 2) making chapters shorter; 3) adding more interactive material

(animations, videos); and 4) improving the flow of information throughout. All these improvements are now incorporated in the final versions of both BIP TIC and the active control condition.

PPI-activities have also been conducted by our UK collaborators in Nottingham/London, which has helped us develop BIP TIC further. This combined PPI-input has shaped BIP TIC and the control condition to the current versions that will be used in the trial. We plan to communicate the trial to patient organisations (Riksförbundet Attention and Svenska OCD-förbundet) and highlight the possibility for members to take part as participants. No further PPI-activities are planned for the current trial.

11 DATA SHARING

We will potentially share trial data with other TD researchers around the world. The use will primarily be for similar objectives as in the current trial, for instance to combine treatment outcome data for meta analyses. Shared data will be pseudonymised. The possibility of future data sharing is mentioned in the informed consent form.

12 ETHICAL CONSIDERATIONS

In our view, the proposed programme of work poses little or no risk to participants. All participants will be offered a thorough psychiatric assessment, access to a dedicated therapist throughout the trial, and will be followed-up long-term. No participant will be denied any current standard treatment (other than face-to-face behaviour therapy, which is very rarely available in Sweden, between enrolment and the primary endpoint). All participant families will be volunteers, competent to provide informed consent. Participants may withdraw from the trial at any time.

It is unlikely that participants will experience any serious adverse events during this trial as a result of the two treatments. Adverse events will be carefully monitored throughout the trial. There will be close liaison between the participant and the therapist throughout the trial so that any adverse reactions can be noted. All participants that experience an adverse event will be followed-up until the event is resolved. Where necessary, the participant's local teams or GP will be informed about the event.

BIP TIC differs from face-to-face therapy only in the format and mode of delivery, but still contains the same evidence-based ingredients. A thorough diagnostic and mental health status examination before enrolment will ensure that patients with more immediate needs or risks are not included in the trial and are offered alternative treatment. The regular text communication and the possibility for the participating families to contact their therapist at any time, ensures that appropriate action can be taken should any unforeseen risks arise.

We predict that the control condition will not be as effective as BIP TIC in reducing tics, but previous literature indicates that it will offer some therapeutic benefits. Education on tics is a key ingredient in all current behavioural therapy protocols for TD. Given that the majority of patients in Sweden do not receive access to any form of behavioural treatment, we believe that the control intervention will still outweigh any benefit of standard care (which is typically no therapeutic intervention). Moreover, in both arms (BIP TIC and the control condition), participants are permitted to continue on any tic medication and thus both groups will be receiving support for their tics, over and above standard care. Additionally, as noted above, if the family's problems are too severe for this trial, or the child is considered to be a risk to self or others, they are not considered eligible for the trial.

Participants may worry about computer safety and confidentiality. To prevent this, families will receive information about the risks and precautions that are being taken when using communication technology (e.g., encrypted server technology and double authentication with password and SMS-code).

Each participant will be assigned a unique trial identification number at the point of randomisation. This number will be recorded on all paper datasheets and in the electronic trial database. A hard copy of a record sheet linking patient identity, contact details, screening number, and trial identification number for all participants will be kept securely in a locked filing cabinet separate from datasheets. All data will be kept secure at all times and maintained in accordance with the requirements of GCP regulations. ELN will be used to log all significant decisions during the trial and to lock the final dataset before unblinding.

All staff will be GCP-trained. Swedish and international regulations will be followed and our research team has extensive experience and knowledge of the approval processes, systems, and good practice guidelines for clinical research in Sweden and internationally.

13 IMPLICATIONS

The planned trial is directly aligned with the principles of the Government's Vision for eHealth 2025 [64] which aims to place Sweden at the forefront of digitalisation and eHealth. The healthcare system is under pressure due to rising healthcare costs. Moreover, quality and access to healthcare are inequitably distributed across the country. Surveys indicate that both the Swedish society [65] and health professionals [66] are open to eHealth solutions that can facilitate access to healthcare.

The majority of children with TD do not receive their preferred treatment due to a lack of available expertise, long travel distances, or other barriers. BIP TIC has the potential to increase access to evidence-based treatment, reaching many more patients who would otherwise remain untreated.

If our hypotheses are correct and BIP TIC is an efficacious, durable, and cost-effective intervention for young people with TD, we will aim to directly implement it in regular healthcare via Sweden's national *Stöd och behanding* (English: *'Support and treatment'*) platform [67].

14 FINANCE

The trial is financed by Forte (grant number 2017-01066), ALF (grant number 20180093), and Swedish Research Council (grant number 2018-00344).

15 PUBLICATION PLANS

We plan to publish two primary papers. Paper 1 will report efficacy results at the primary endpoint (3 months post-treatment), including a short-term health economic evaluation. Paper 2 will report efficacy results for the naturalistic long-term follow-up period (up to 12 months post-treatment), also including a long-term health economic evaluation. Additional papers may include analysis of predictors and moderators of treatment outcome. All these papers will be submitted to general or specialty medical and psychological science journals.

16 REFERENCES

1. Hollis, C., et al., *Clinical effectiveness and patient perspectives of different treatment strategies for tics in children and adolescents with Tourette syndrome: a systematic review and qualitative analysis.* 2016.
2. Freeman, R.D., et al., *An international perspective on Tourette syndrome: selected findings from 3500 individuals in 22 countries.* Developmental medicine and child neurology, 2000. **42**(7): p. 436-447.
3. Verdellen, C., et al., *European clinical guidelines for Tourette syndrome and other tic disorders. Part III: behavioural and psychosocial interventions.* European child & adolescent psychiatry, 2011. **20**(4): p. 197-207.
4. Whittington, C., et al., *Practitioner Review: Treatments for Tourette syndrome in children and young people–a systematic review.* Journal of Child Psychology and Psychiatry, 2016. **57**(9): p. 988-1004.
5. Roessner, V., et al., *European clinical guidelines for Tourette syndrome and other tic disorders. Part II: pharmacological treatment.* European child & adolescent psychiatry, 2011. **20**(4): p. 173-196.
6. Carulla-Roig, M., et al., *Pharmacoepidemiology of Tourette and Chronic Tic Disorders in Sweden 2005-2013.* J Child Adolesc Psychopharmacol, 2018.
7. Cuenca, J., et al., *Perceptions of treatment for tics among young people with Tourette syndrome and their parents: a mixed methods study.* BMC psychiatry, 2015. **15**(1): p. 46.
8. Andersson, G., et al., *Guided Internet-based vs. face-to-face cognitive behavior therapy for psychiatric and somatic disorders: a systematic review and meta-analysis.* World Psychiatry, 2014. **13**(3): p. 288-95.

9. Hedman, E., B. Ljotsson, and N. Lindefors, *Cognitive behavior therapy via the Internet: a systematic review of applications, clinical efficacy and cost-effectiveness.* Expert Rev Pharmacoecon Outcomes Res, 2012. **12**(6): p. 745-64.
10. Vigerland, S., et al., *Internet-delivered cognitive behavioural therapy for children with anxiety disorders: A randomised controlled trial.* Behav Res Ther, 2016. **76**: p. 47-56.
11. Vigerland, S., et al., *Internet-delivered CBT for children with specific phobia: a pilot study.* Cogn Behav Ther, 2013. **42**(4): p. 303-14.
12. Lenhard, F., et al., *Internet-delivered cognitive behavior therapy for adolescents with obsessive-compulsive disorder: an open trial.* PLoS One, 2014. **9**(6): p. e100773.
13. Lenhard, F., et al., *Therapist-Guided, Internet-Delivered Cognitive-Behavioral Therapy for Adolescents With Obsessive-Compulsive Disorder: A Randomized Controlled Trial.* J Am Acad Child Adolesc Psychiatry, 2017. **56**(1): p. 10-19 e2.
14. Bonnert, M., et al., *Internet-Delivered Cognitive Behavior Therapy for Adolescents With Irritable Bowel Syndrome: A Randomized Controlled Trial.* Am J Gastroenterol, 2017. **112**(1): p. 152-162.
15. Andrén, P., et al., *Therapist- and parent-guided Internet-delivered behaviour therapy for paediatric Tourette's Disorder: a pilot randomised controlled trial with long-term follow-up.* Under review, 2018.
16. Hollis, C., et al., *Clinical effectiveness and patient perspectives of different treatment strategies for tics in children and adolescents with Tourette syndrome: a systematic review and qualitative analysis.* Health Technology Assessment, 2016. **20**(4): p. 1-450.
17. Piacentini, J., et al., *Behavior therapy for children with Tourette disorder: a randomized controlled trial.* JAMA, 2010. **303**(19): p. 1929-37.
18. Andersson, G., et al., *Guided Internet - based vs. face - to - face cognitive behavior therapy for psychiatric and somatic disorders: a systematic review and meta - analysis.* World Psychiatry, 2014. **13**(3): p. 288-295.
19. Hedman, E., B. Ljótsson, and N. Lindefors, *Cognitive behavior therapy via the Internet: a systematic review of applications, clinical efficacy and cost–effectiveness.* Expert review of pharmacoeconomics & outcomes research, 2012. **12**(6): p. 745-764.
20. Johansson, R. and G. Andersson, *Internet-based psychological treatments for depression.* Expert review of neurotherapeutics, 2012.
21. Cuijpers, P., et al., *Self-guided psychological treatment for depressive symptoms: a meta-analysis.* PloS one, 2011. **6**(6): p. e21274.
22. Lenhard, F., et al., *Internet-delivered cognitive behavior therapy for adolescents with obsessive-compulsive disorder: an open trial.* PLoS One, 2014. **9**(6): p. e100773.
23. Vigerland, S., et al., *Internet-delivered cognitive behavioural therapy for children with anxiety disorders: A randomised controlled trial.* Behaviour research and therapy, 2016. **76**: p. 47-56.
24. Vigerland, S., et al., *Internet-delivered CBT for children with specific phobia: a pilot study.* Cognitive behaviour therapy, 2013. **42**(4): p. 303-314.
25. Hollis, C., et al., *Annual Research Review: Digital health interventions for children and young people with mental health problems –a systematic and meta - review.* Journal of Child Psychology and Psychiatry, 2017. **58**(4): p. 474-503.
26. Himle, M.B., et al., *A randomized pilot trial comparing videoconference versus face-to-face delivery of behavior therapy for childhood tic disorders.* Behaviour research and therapy, 2012. **50**(9): p. 565-570.
27. Ricketts, E.J., et al., *A randomized waitlist-controlled pilot trial of voice over Internet protocol-delivered behavior therapy for youth with chronic tic disorders.* Journal of telemedicine and telecare, 2016. **22**(3): p. 153-162.

28. Leckman, J.F., et al., *The Yale Global Tic Severity Scale: initial testing of a clinician-rated scale of tic severity.* Journal of the American Academy of Child and Adolescent Psychiatry, 1989. **28**(4): p. 566-73.
29. Geraci, M. and M. Bottai, *Quantile regression for longitudinal data using the asymmetric Laplace distribution.* Biostatistics, 2007. **8**(1): p. 140-154.
30. Geraci, M. and M. Bottai, *Linear quantile mixed models.* Statistics and Computing, 2014. **24**(3): p. 461-479.
31. Liu, Y. and M. Bottai, *Mixed-Effects Models for Conditional Quantiles with Longitudinal Data.* International Journal of Biostatistics, 2009. **5**(1).
32. American Psychiatric Association, *Diagnostic and Statistical Manual of Mental Disorders: DSM-5*. 5th ed. 2013, Washington, D.C.: American Psychiatric Publishing. xliv, 947 p.
33. Sheehan, D.V., et al., *Reliability and validity of the Mini International Neuropsychiatric Interview for Children and Adolescents (MINI-KID).* Journal of Clinical Psychiatry, 2010. **71**(3): p. 313-26.
34. Allison, C., B. Auyeung, and S. Baron-Cohen, *Toward brief "Red Flags" for autism screening: The Short Autism Spectrum Quotient and the Short Quantitative Checklist for Autism in toddlers in 1,000 cases and 3,000 controls [corrected].* J Am Acad Child Adolesc Psychiatry, 2012. **51**(2): p. 202-212 e7.
35. Swanson, J.M., et al., *Clinical relevance of the primary findings of the MTA: success rates based on severity of ADHD and ODD symptoms at the end of treatment.* J Am Acad Child Adolesc Psychiatry, 2001. **40**(2): p. 168-79.
36. American Psychiatric Association. and American Psychiatric Association. Task Force on DSM-IV., *Diagnostic and statistical manual of mental disorders : DSM-IV*. 4th ed. 1994, Washington, DC: American Psychiatric Association. xxvii, 886 p.
37. Bussing, R., et al., *Parent and teacher SNAP-IV ratings of attention deficit hyperactivity disorder symptoms: psychometric properties and normative ratings from a school district sample.* Assessment, 2008. **15**(3): p. 317-28.
38. Woods, D.W., et al., *Premonitory Urge for Tics Scale (PUTS): initial psychometric results and examination of the premonitory urge phenomenon in youths with Tic disorders.* Journal of Developmental & Behavioral Pediatrics, 2005. **26**(6): p. 397-403.
39. Jeon, S., et al., *Detecting a clinically meaningful change in tic severity in Tourette syndrome: a comparison of three methods.* Contemp Clin Trials, 2013. **36**(2): p. 414-20.
40. Guy, W., *ECDEU assessment manual for psychopharmacology.* US Department of Health, and Welfare, 1976: p. 534-537.
41. Busner, J. and S.D. Targum, *The clinical global impressions scale: applying a research tool in clinical practice.* Psychiatry (Edgmont), 2007. **4**(7): p. 28-37.
42. Shaffer, D., et al., *A children's global assessment scale (CGAS).* Archives of General Psychiatry, 1983. **40**(11): p. 1228-31.
43. Green, B., et al., *The Children's Global Assessment Scale in clinical practice: An empirical evaluation.* Journal of the American Academy of Child & Adolescent Psychiatry, 1994. **33**(8): p. 1158-1164.
44. Simpson, H.B., et al., *Development of a Patient Adherence Scale for Exposure and Response Prevention Therapy.* Behavior Therapy, 2010. **41**(1): p. 30-37.
45. Cavanna, A.E., et al., *Disease-specific quality of life in young patients with Tourette syndrome.* Pediatric neurology, 2013. **48**(2): p. 111-114.
46. Su, M.T., et al., *The English Version of the Gilles de la Tourette Syndrome–Quality of Life Scale for Children and Adolescents (C&A-GTS-QOL) A Validation Study in the United Kingdom.* Journal of child neurology, 2017. **32**(1): p. 76-83.
47. Angold, A., et al., *Development of a short questionnaire for use in epidemiological studies of depression in children and adolescents.* International journal of methods in psychiatric research, 1995.

48. Burleson Daviss, W., et al., *Criterion validity of the Mood and Feelings Questionnaire for depressive episodes in clinic and non - clinic subjects.* Journal of Child Psychology and Psychiatry, 2006. **47**(9): p. 927-934.
49. Foa, E.B., et al., *Development and validation of a child version of the obsessive compulsive inventory.* Behavior Therapy, 2010. **41**(1): p. 121-32.
50. Andrusyna, T.P., et al., *The factor structure of the Working Alliance Inventory in cognitive-behavioral therapy.* The Journal of psychotherapy practice and research, 2001. **10**(3): p. 173.
51. The KIDSCREEN Group Europe, *The KIDSCREEN Questionnaires - Quality of life questionnaires for children and adolescents. Handbook. :*. 2006, Lengerich: Pabst Science Publishers.
52. Ravens-Sieberer, U., et al., *KIDSCREEN-52 quality-of-life measure for children and adolescents.* Expert Rev Pharmacoecon Outcomes Res, 2005. **5**(3): p. 353-64.
53. Chen, G., et al., *From KIDSCREEN-10 to CHU9D: creating a unique mapping algorithm for application in economic evaluation.* Health Qual Life Outcomes, 2014. **12**: p. 134.
54. Chang, S., et al., *Initial Psychometric Properties of a Brief Parent-Report Instrument for Assessing Tic Severity in Children with Chronic Tic Disorders.* Child & Family Behavior Therapy, 2009. **31**(3): p. 181-191.
55. Hill, P. and E. Taylor, *An auditable protocol for treating attention deficit/hyperactivity disorder.* Archives of disease in childhood, 2001. **84**(5): p. 404-409.
56. Bouwmans, C., et al., *Feasibility, reliability and validity of a questionnaire on healthcare consumption and productivity loss in patients with a psychiatric disorder (TiC-P).* BMC Health Serv Res, 2013. **13**: p. 217.
57. Verdellen, C.W., et al., *Tics - Therapist Manual*. 2011, Amsterdam: Boom Publishers.
58. Woods, D.W., et al., *Managing Tourette syndrome : a behavioral intervention for children and adults : therapist guide*. Treatments that work. 2008, Oxford, New York: Oxford University Press. ix, 132 p.
59. Moher, D., et al., *CONSORT 2010 Explanation and Elaboration: Updated guidelines for reporting parallel group randomised trials.* J Clin Epidemiol, 2010. **63**(8): p. e1-37.
60. Drummond, M., et al., *Methods for the economic evaluation of health care programmes*. New York, NY, ed. O.U. Press. 2005.
61. Matthews, J.N., et al., *Analysis of serial measurements in medical research.* BMJ, 1990. **300**(6719): p. 230-5.
62. Barber, J. and S. Thompson, *Multiple regression of cost data: use of generalised linear models.* J Health Serv Res Policy, 2004. **9**(4): p. 197-204.
63. Sukhodolsky, D.G., et al., *Moderators and predictors of response to behavior therapy for tics in Tourette syndrome.* Neurology, 2017.
64. Government Offices of Sweden - Ministry of Health and Social Affairs and Swedish Association of Local Authorities and Regions, *Vision for eHealth 2025 – common starting points for digitisation of social services and health care*, ed. G.O.o. Sweden. 2016, Stockholm, Sweden.
65. pwc. *The doctor is in – your smartphone Is Sweden ready for digital and virtual care?* 2015 2017-05-09]; Available from: http://www.pwc.se/sv/pdf-reports/the-doctor-is-in-your-smartphone.pdf.
66. pwc. *The digital patient is here – but is healthcare ready?* 2016 2017-05-09]; Available from: https://www.pwc.se/sv/pdf-reports/the-digital-patient-is-here.pdf.
67. *Stöd och behandling*. [cited 2021 21 January]; Available from: https://vardgivarguiden.se/it-stod/e-tjanster-och-system/1177/stod-och-behandling-sob/.

BIP TIC RCT - Statistical and health economic analysis plan

Version 1.0, 2021-02-03.

1 Introduction

1.1 Purpose

This statistical analysis plan (SAP) contains details of the main statistical analyses for the BIP TIC randomised controlled trial (RCT). These analyses are pre-specified in order for them not to be influenced by the collected trial data after unblinding. This SAP describes the statistical analysis of the longitudinal clinical outcomes and the health economic evaluation.

The SAP does not preclude the undertaking of further post-hoc or exploratory analyses, although the results of such analyses should be interpreted with caution. Furthermore, the SAP does not preclude the modification of any part of the trial analysis, should particular situations arise in which such adaptation is deemed necessary. Such adaptations, if performed, will be documented in the Karolinska Institutet Electronic Lab Notebook, and clearly labelled as post-hoc in the published paper.

1.2 Trial registration and protocol

The trial was prospectively registered at ClinicalTrials.gov (NCT03916055). Full information on the trial design, interventions, assessment points, measures, etc. is described in the full study protocol (current version 3.0). This SAP should be interpreted in the context of the information provided in the full study protocol.

1.3 Authorship

The SAP has been written by Per Andrén (PA; study coordinator and co-investigator) and edited by David Mataix-Cols (DMC; principal investigator), Lorena Fernández de la Cruz (LFC; co-investigator), Erik Andersson (EA; co-investigator), Matteo Bottai (MB; statistician), Inna Feldman (IF; health economist), and Filipa Sampaio (FS; health economist).

2 Study objectives

2.1 Primary objective

Objective #1: To determine the clinical efficacy of internet-delivered behaviour therapy (hereby referred to as BIP TIC) for reducing tic severity (as measured by the Yale Global Tic Severity Scale - Total Tic Severity Score [YGTSS-TTSS]; *primary outcome measure* [1]) in children and adolescents with Tourette's Disorder and Persistent (Chronic) Motor or Vocal Tic Disorder [2], compared with an active control intervention (hereby referred to as *the comparator*). The *primary endpoint* is the 3-month follow-up after the end of treatment (3FU).

Appendix

2.2 Secondary objectives

Objective #2: To establish the 12-month durability of the above-described treatment effects.

Objective #3: To conduct a health economic evaluation of BIP TIC, from multiple perspectives, both in the short term (primary endpoint) and the long term (12-month follow-up)..

3 Reporting

We will follow the Consolidated Standards of Reporting Trials (CONSORT) 2010 guidelines [3] when reporting clinical outcomes, and the Consolidated Health Economic Evaluation Reporting Standards (CHEERS) guidelines [4] when reporting health economic results.

We plan to publish two primary papers. Paper 1 will report efficacy results at the primary endpoint (3FU; objective #1), including a health economic evaluation (objective #3). Paper 2 will report on the long-term durability of the treatment effects (from 3 to 12 months after the end of treatment; objective #2), and also include a health economic evaluation for this time period (objective #3). Potential additional papers may include analyses of predictors and moderators of the treatment outcome (objectives yet to be determined); all these analyses will be considered post-hoc.

4 Statistical analyses of clinical outcomes

4.1 Responsibility

Statistical analyses for objectives #1 and #2 (clinical efficacy and 12-month durability of BIP TIC) will be performed by PA and then independently replicated by MB. Both PA and MB will be blind to group allocation during the data analyses.

4.2 Organisation of data and analyses

Data are collected at 5-7 assessment points, depending on the outcome measure. Data points include baseline, 3 weeks into treatment (3WK), 5 weeks into treatment (5WK), directly after the 10-week treatment (post-treatment), and 3, 6, and 12 months post-treatment (3FU, 6FU, and 12FU, respectively). Analyses will be performed in two stages. Stage 1 corresponds to paper 1 (see section 3. Reporting), and will contain data collected between baseline and the 3FU (the primary endpoint). Stage 2 corresponds to paper 2 (see section 3. Reporting), and will contain data collected at all assessment points, from baseline up to the 12FU. The two-stage analysis will require two corresponding database locks. The primary outcome analysis will be carried out in stage 1 (corresponding to objective #1).

The primary data analysis will be conducted after the last participant has completed the 3FU and all collected data have been entered into the trial database. Assessors will however remain blind for the individual participants' treatment allocations until data for the final 12FU assessment has been conducted. The SAP will be finalised, approved, and published prior to unblinding.

Prior to each database lock, basic checks will be performed on the collected data to ensure accuracy. PA, who will be blind to group allocation, will conduct these checks. Each baseline demographic variable and outcome variables (both primary and secondary) will be checked for:

- Missing values
- Values outside an acceptable range
- Other inconsistencies

If missing values or other inconsistencies are found, PA will investigate them and compare the data in the databases with the source data (if available). The data will then be either corrected or deemed as missing, as appropriate. The database will then be locked for data changes by PA. To ensure blinding, a researcher not involved in the analysis will create provisional dummy variables in place for the group allocation and participant ID variables, so that the researchers performing the analysis are not influenced by the knowledge of group allocation.

The statistical programming code to be used for the primary analysis (objective #1) will be prepared prior to unblinding. The primary analysis (objective #1) will be performed by PA and independently replicated by MB.

4.3 Interim analyses

No interim analyses are planned.

4.4 Recruitment and retention

A CONSORT 2010 flow diagram [3] will be presented to provide a detailed description of the participant flow throughout the study, including number of participants screened, reasons for exclusion, number of participants randomised to each group, and number of participants assessed at each follow-up point. See CONSORT flow diagram [3] for the exact content that will be presented.

4.5 Description of demographic variables at baseline

The demographic information collected at baseline will be summarised and presented in a table, by randomisation arm. Categorical variables will be reported as counts and percentages. Continuous variables will be summarised as means, medians, and interquartile ranges, as appropriate. According to CONSORT recommendations, no statistical tests will be performed to assess baseline differences between study arms [5].

4.6 Protocol deviations

Protocol deviations which could impact on the results of the analyses (such as concomitant psychological treatment or medication) will be described in detail. Further analyses taking such deviations into account (e.g., sensitivity analyses) may be performed, depending on the amount and extent of the deviations. All such analyses will be considered post-hoc.

Appendix

4.7 Primary outcome analysis (objective #1)

The primary outcome is tic severity, as measured by the YGTSS-TTSS, at the primary endpoint (3FU). The primary outcome analysis (objective #1) will be based on all available data up to this time point and conducted according to the intention-to-treat (ITT) principle.

As the YGTSS-TTSS is a composite measure resulting in an integer-valued variable ranging from 0 to 50, statistical modelling will focus on the median of the outcome (i.e., the 0.5-quantile) rather than its mean. Specifically, all the randomised participants will be included to estimate a linear quantile mixed model [6-8], taking into account individual differences in pre-treatment symptomatic status and treatment response. The model will include fixed effects for time (the YGTSS-TTSS at baseline, post-treatment, and 3-month follow-up) and subject-specific effects as a random intercept factor to account for the variances between and within participants. Linear quantile mixed models use all available data, can account for correlation between repeated measurements on the same subject, can flexibly model time effects, and can handle missing data.

To enable comparisons with previous trials within the field, which traditionally have used regression models based on means rather than medians, we will also perform complementary analyses of the primary outcome measure at the primary endpoint using a linear mixed model (estimating a difference in means). Similar to the linear quantile mixed model, the linear mixed model will include fixed effects of time (the YGTSS-TTSS at baseline, post-treatment, and 3-month follow-up) and subject effects as a random intercept factor to account for the variances between and within participants. Like linear quantile mixed models, linear mixed-effects models use all available data, can account for correlation between repeated measurements on the same subject, can flexibly model time effects, and can handle missing data. Results from the linear mixed-effects model will be presented as a complementary analysis to the linear quantile mixed model.

The estimated treatment effect will be reported with the accompanying 95% confidence interval (CI) and *p*-value. The alpha level of 0.05 will be used as the threshold for statistical significance throughout all trial analyses.

4.7.1 Model checking

The statistical model for the primary outcome analysis includes an assumption that the subject-specific random intercepts are normally distributed. This assumption will be checked through the construction of appropriate histograms and normal quantile plots. If these plots suggest that residuals are not normally distributed, then appropriate transformations of the primary outcome or application of the bootstrap method will be considered.

4.7.2 Missing data

Potential selection bias caused by missing data will be investigated by comparing the baseline characteristics of participants with and without missing values on the YGTSS-TTSS using logistic regression. Depending on the amount of missing values, predictors of missingness will be identified. We will then perform a sensitivity analysis by including any predictors of missingness as explanatory variables in the primary outcome model. Analyses investigating the impact of missing data will be considered supportive.

Appendix

4.8 Analyses of secondary outcomes

Secondary outcome measures (details shown in the full study protocol) will be analysed using similar statistical methods as the primary outcome, that is, with linear quantile mixed models, and complemented with linear mixed models to facilitate comparison with previous trials. Dichotomous variables will be analysed using logistic regression. The results will be presented as estimates or odds ratios, as appropriate, for the regression coefficients, with their respective 95% confidence intervals and *p*-values.

4.9 Treatment response

Treatment response will be operationalised as scores of “very much improved” (1) or “much improved” (2) on the Clinical Global Impression – Improvement scale (CGI-I) administered at 3FU [9]. We will also report numbers needed to treat, with 95% confidence intervals, based on the proportions of responders in each group at 3FU.

4.10 Naturalistic long-term follow-up analyses (objective #2)

All trial participants will be naturalistically followed up (i.e., participants will be able to seek other kinds of help after the primary endpoint at 3FU) and assessed at 6FU and 12FU. As previously described, the blind assessors will remain blind for the individual participants’ treatment allocations until data for the final 12FU assessment have been collected. The naturalistic long-term follow-up analyses will be carried out in stage 2 (corresponding to objective #2). The analyses will be performed on the second locked database and will be published in paper 2.

The stage 2 analyses will be carried out using linear quantile mixed models and logistic regression, which are described for stage 1. In addition, we will perform complementary linear mixed models (estimating a difference in means) for each of the linear quantile mixed models performed. The linear mixed models will be performed to enable comparisons with previous trials in the field.

Primarily, the stage 2 regression models will include the 3FU, 6FU, and 12FU assessment points, and will evaluate in a within-group analysis whether the potential treatment effects demonstrated for BIP TIC at 3FU in stage 1 are sustained at 12FU.

Additionally, we will also enter all available assessment points to a separate regression model to investigate whether there are significant between-group effects at 12FU. Results from such between-group analyses, however, will be interpreted with caution, given the risk of bias associated with long follow-up periods (e.g., patients with poor short-term outcomes are more prone to receive other potentially effective treatments during the follow-up period) [10].

4.11 Subgroup analyses

No subgroup analyses are planned.

4.12 Effect-sizes

For median comparisons, the magnitude of the treatment effects will be presented as between-group differences in median relative the interquartile range. For mean comparisons, the magnitude

of the treatment effects will be presented as standardised between-group effect sizes (Cohen's *d*) [11]. The latter method will be used to enable comparisons with previous trials in the field.

4.13 Adverse events

Adverse events (AEs) will be reported separately by treatment group at the 5WK, post-treatment, and 3FU assessment points. The AEs will be summarized by number (frequencies and percentages), nature, severity, and whether they are expected or unexpected. If there are any serious AEs, PA and DMC will judge whether these are related to the treatments provided. No statistical tests comparing the treatment groups will be performed. See the full study protocol for detailed information regarding the data collection of AEs, including definitions of expected and unexpected AEs.

4.14 Reliability

Inter-rater reliability of the YGTSS-TTSS will be estimated by intra-class correlation coefficients (ICC). ICC will be calculated on case examples before the study start, as well as on actual study participants during the trial.

5 Health Economic Evaluation (objective #3)

5.1 Responsibility

Analyses for objective #3 (health economic evaluation of BIP TIC) will be performed by PA and independently replicated by IF and FS.

5.2 Analyses

The short-term health economic evaluation (objective #3) will compare BIP TIC and the comparator at the primary endpoint (3FU). These results will be published together with the efficacy results (objective #1) in paper 1. As a long-term evaluation, we will then perform the same analyses at the end of the follow-up period (12FU) using cumulative information collected throughout the follow-up. These results will be published together with the results of the naturalistic follow-up (objective #2) in paper 2.

The health economic evaluation will be performed from three different perspectives, with gradually increasing costs included for each perspective:

1. The first perspective is the health organisation payer perspective, which includes direct treatment costs, of BIP TIC and the comparator, for the clinic. This comprises personnel costs such as therapist time, administration time, IT platform maintenance, and other overheads.
2. The second perspective will additionally include other healthcare resource use outside the clinic for the children, such as costs for medical appointments (outside the trial) and medications.
3. The third perspective will further comprise other societal costs, including productivity losses for the children related to absenteeism and presenteeism from school and leisure activities, and productivity losses for the parents due to absenteeism from work.

Appendix

The Trimbos/iMTA Questionnaire for Costs associated with Psychiatric Illness (TiC-P [12]) will be administered (in an adapted version for young people and parents) at each assessment point (except 3 and 5 weeks into the treatment) to collect information on frequencies of resource use for the children and absenteeism from work for the parents. Resource use costs will be estimated by multiplying frequencies by national Swedish tariffs and market prices. Total costs for each group will be aggregated over the trial period.

For each of the three perspectives above, we will conduct two types of analyses:

1. A cost-effectiveness analysis, using responder rate as the outcome [13].
2. A cost-utility analysis using Quality Adjusted Life Years (QALYs) as the outcome [13].

Health gains in terms of QALYs will be estimated by mapping KIDSCREEN-10 scores [14] onto Child Health Utility 9 Dimensions (CHU9D) utility weights [15]. Total QALY gains over the trial period will be estimated by using the area under the curve method [16]. Differences in QALYs and costs between both trial arms will be investigated using generalised linear models with suitable distributions [17].

To ascertain whether BIP TIC is cost-effective, relative to the comparator, incremental cost-effectiveness ratios expressed as cost per additional responder and cost per additional QALY will be presented. The uncertainty around the cost and effect estimates will be presented using a cost-effectiveness acceptability curve [13].

6 References

1. Leckman JF, Riddle MA, Hardin MT, Ort SI, Swartz KL, Stevenson J, Cohen DJ (1989) The Yale Global Tic Severity Scale: initial testing of a clinician-rated scale of tic severity. J Am Acad Child Adolesc Psychiatry 28: 566-573
2. American Psychiatric Association (2013) Diagnostic and Statistical Manual of Mental Disorders: DSM-5. 5th edn. American Psychiatric Publishing, Washington, D.C.
3. Schulz KF, Altman DG, Moher D (2010) CONSORT 2010 Statement: updated guidelines for reporting parallel group randomised trials. BMJ 340
4. Husereau D, Drummond M, Petrou S, Carswell C, Moher D, Greenberg D, Augustovski F, Briggs AH, Mauskopf J, Loder E, Force CT (2013) Consolidated Health Economic Evaluation Reporting Standards (CHEERS) statement. BMJ 346: f1049
5. Moher D, Hopewell S, Schulz KF, Montori V, Gotzsche PC, Devereaux PJ, Elbourne D, Egger M, Altman DG, Consolidated Standards of Reporting Trials G (2010) CONSORT 2010 Explanation and Elaboration: Updated guidelines for reporting parallel group randomised trials. J Clin Epidemiol 63: e1-37
6. Geraci M, Bottai M (2007) Quantile regression for longitudinal data using the asymmetric Laplace distribution. Biostatistics 8: 140-154
7. Geraci M, Bottai M (2014) Linear quantile mixed models. Stat Comput 24: 461-479
8. Liu Y, Bottai M (2009) Mixed-Effects Models for Conditional Quantiles with Longitudinal Data. International Journal of Biostatistics 5
9. Busner J, Targum SD (2007) The clinical global impressions scale: applying a research tool in clinical practice. Psychiatry (Edgmont) 4: 28-37
10. Herbert RD, Kasza J, Bo K (2018) Analysis of randomised trials with long-term follow-up. BMC Med Res Methodol 18: 48
11. Cohen J (1988) Statistical Power Analysis for the Behavioral Sciences. 2nd ed. Routledge, Hillsdale, NJ
12. Bouwmans C, De Jong K, Timman R, Zijlstra-Vlasveld M, Van der Feltz-Cornelis C, Tan Swan S, Hakkaart-van Roijen L (2013) Feasibility, reliability and validity of a questionnaire on healthcare

consumption and productivity loss in patients with a psychiatric disorder (TiC-P). BMC Health Serv Res 13: 217
13. Drummond M, Sculpher M, Torrance G, O'Brien B, Stoddart G (2005) Methods for the economic evaluation of health care programmes. New York, NY.
14. The KIDSCREEN Group Europe (2006) The KIDSCREEN Questionnaires - Quality of life questionnaires for children and adolescents. Handbook. : . Pabst Science Publishers, Lengerich
15. Chen G, Stevens K, Rowen D, Ratcliffe J (2014) From KIDSCREEN-10 to CHU9D: creating a unique mapping algorithm for application in economic evaluation. Health Qual Life Outcomes 12: 134
16. Matthews JN, Altman DG, Campbell MJ, Royston P (1990) Analysis of serial measurements in medical research. BMJ 300: 230-235
17. Barber J, Thompson S (2004) Multiple regression of cost data: use of generalised linear models. J Health Serv Res Policy 9: 197-204
