# Supplementary material for: Efficacy and cost-effectiveness of therapist-guided internet-delivered behaviour therapy for children and adolescents with Tourette syndrome: study protocol for a single-blind randomised controlled trial
Source: Trials. 2021 Sep 30;22:669. doi: 10.1186/s13063-021-05592-z (PMC8481317; doi:10.1186/s13063-021-05592-z)
Supplement: Supplementary file 4 — Additional file 4: Supplementary file 4. Funding documents in Swedish (original) and English (translation). [file 13063_2021_5592_MOESM4_ESM.pdf]

FORTE

Forskningsrådet för hälsa, arbetsliv och välfärd

BESLUT
Beslutnr: STYA-2017/0003
Beslutsdatum: 2017-09-27

BESLUT OM BIDRAG

Diarienummer: 2017-01066
Projektledare: David Mataix-Cols
Projekttitel: Klinisk- och kostnadseffektivitet av internetförmedlad beteendeterapi för barn och ungdomar med Tourettes syndrom

Beslutsinstans: STYA - Styrelsebeslut
Handläggare: Dag Hervieu

BESLUT
Forskningsrådet för hälsa, arbetsliv och välfärd har beslutat att ge bidrag enligt nedan:
2016-01-01 - 2016-12-31: 0 SEK
2017-01-01 - 2017-12-31: 0 SEK
2018-01-01 - 2018-12-31: 1 000 000 SEK
2019-01-01 - 2019-12-31: 1 000 000 SEK
2020-01-01 - 2020-12-31: 1 030 000 SEK

Beslutet tilldelas
Karolinska Institutet
Projektledare: David Mataix-Cols

Swedish Research Council for Health, Working Life and Welfare

Decision
Decision no.: STYA-2017/0003
Decisions date: 2017-09-27

DECISION ABOUT FUNDING

Diary number: 2017-01066
Principal investigator: David Mataix-Cols
Project title: Clinical and cost-effectiveness of internet-delivered behaviour therapy for children and adolescents with Tourette syndrome

Decision authority: STYA – Board decision
Administrator: Dag Hervieu

DECISION

The Swedish Research Council for Health, Working Life and Welfare has decided to grant funds according to below:
2016-01-01 - 2016-12-31: 0 SEK
2017-01-01 - 2017-12-31: 0 SEK
2018-01-01 - 2018-12-31: 1 000 000 SEK
2019-01-01 - 2019-12-31: 1 000 000 SEK
2020-01-01 - 2020-12-31: 1 030 000 SEK

The decision is granted to
Karolinska Institutet
Principal investigator: David Mataix-Cols

Stockholms läns landsting

Anslag forskning, utveckling och utbildning

/Driftmiljö

Min ansökan nr 20180093

Startsida
Utlysningar/anvisn.
Ansök här
Min ansökan
Beviljade anslag
Populärvet. beskr.
Statistik
Fakturering
Beredning
Granskningsgrupper
Acknowledgement

Beslutsuppgifter

» Se mina ansökningsuppgifter » Lämna rapport » Avsluta / logga ut

Här finns aktuell information för din ansökan. Du kan även se uppgifterna som du lämnat i din ansökan.

» Ändra huvudsökandes adressuppg. mm
» Ändra lösenord för ansökan.

Huvudsökande: Kayoko Isomura

Anslagsform: ALF projektmedel medicin, ny ansökan

Prioriteringsgrupp: PG 2b Psykiska sjukdomar och beroende

Projekttitel: Further development and evaluation of a remote digital behavioural intervention for children and adolescents with Tourette's syndrome

Medelpoängvärden:

Projektets kliniska frågeställning	5
Metod och vetenskaplig kvalitet	4,67
Kompetens och genomförbarhet	5
Patientnytta	4,83

Summerat medelvärde: 19,5

Högst och lägst medelvärde för beviljning i gruppen är 20 - 16

Genusperspektiv: Tillfredsställande

Utlåtande:

Sammanfattande bedömning
En ny beteendeterapi för personer med Tourettes syndrom ska prövas i en RCT. Projektet har bra och tydligt syfte/frågeställning som inte ger några frågetecken om vad som ska göras. Avgränsad och väl beskriven patientgrupp. Tydlig powerberäkning och inklusionskriterium. Mycket tydlig beskrivning av terapeututbildningen för den specifika interventionen. I projektet görs också en beräkning av behandlingens kostnadseffektivitet. Gruppen är väl sammansatt, kliniskt verksamma inom området och mycket kompetenta för projektets genomförbarhet.

Särskilda villkor:

Beslut: Medel beviljade enligt nedan.

	2019	2020	2021
**Beviljat:**	950 000 kr	900 000 kr	700 000 kr
**Disponibelt:**	--- *	--- **	--- **

*/ Beviljat belopp disponeras efter underskrivet kontrakt och när etikprövning är godkänd.
**/ Fleråriga anslag disponeras efter godkänt årligt intyg.

Stockholms läns landsting

Grant research, development and education

My application no. 20180093

Information about the decision

Main applicant: Kayoko Isomura

Grant form: ALF project funds medicine, new application

Priority group: PG 2b Mental illness and addiction

Project title: Further development and evaluation of a remote digital behavioural intervention for children and adolescents with Tourette syndrome

Mean point values: The project's clinical objective: 5
Method and scientific quality: 4,67
Competence and feasibility: 4,67
Patient benefit: 4,83

Summarised mean: 19,5

Highest and lowest mean for granting in the group is 20 – 16.

Gender perspective: Satisfactory

Statement: **Summarised assessment**
A new behavioural therapy for individuals with Tourette syndrome will be evaluated in a RCT. The project has a good and clear aim/objective and it is clear what will be performed. Well defined and described patient group. Clear power calculation and eligibility criteria. Very clear description of the therapist education for the specific intervention. The project will also evaluate the treatment's cost-effectiveness. The group is well put together, with active clinicians within the field, and is very competent for the implementation of the project.

Specific criteria:

Decision: Funds granted according to below.

	**2019**	**2020**	**2021**
**Granted:**	950 000 SEK	900 000 SEK,	700 000 SEK
**Available:**	--- *	--- **	--- **

*/ Granted amount is made available after the contract has been signed and ethical permission has been approved.
**/ Perennial grants are made available after the approval of yearly certificates.

Vetenskapsrådet

Diarienummer: 2018-00344
Projektledare: David Mataix-Cols
Projekttitel: Klinisk- och kostnadseffektivitet av internetförmedlad beteendeterapi för barn och ungdomar med Tourettes syndrom
Datum: 2018-11-22

UNDERRÄTTELSE OM BESLUT OM BIDRAG

Vetenskapsrådet har beslutat att bevilja din ansökan om bidrag. Beviljat belopp och fullständiga villkor återfinns i "Godkännande av villkor".

Denna underrättelse finns tillgänglig för sökande och medelsförvaltare i Vetenskapsrådets ansökningssystem Prisma.

Beslutsinstans: KKBF
Handläggare: Elisabeth Tehler

Vetenskapsrådet

Diary number: 2018-00344
Principal investigator: David Mataix-Cols
Project title: Clinical and cost-effectiveness of internet delivered behaviour therapy for children and adolescents with Tourette syndrome
Date: 2018-11-22

NOTIFICATION OF DECISION ABOUT FUNDING

The Swedish Research Council has decided to grant your application about funding. Granted amount and complete conditions are found in ”Approval of conditions”.

This notification is available to the applicant and the funding manager in the Swedish Research Council’s application system Prisma.

Decision authority: KKBF
Administrator: Elisabeth Tehler
