# Supplementary material for: Efficacy and cost-effectiveness of therapist-guided internet-delivered behaviour therapy for children and adolescents with Tourette syndrome: study protocol for a single-blind randomised controlled trial
Source: Trials. 2021 Sep 30;22:669. doi: 10.1186/s13063-021-05592-z (PMC8481317; doi:10.1186/s13063-021-05592-z)
Supplement: Supplementary file 5 — Additional file 5: Supplementary file 5. Ethical approval documents in Swedish (original) and English (translation). [file 13063_2021_5592_MOESM5_ESM.pdf]

EPN

BESLUT

Dnr: 2018/1788-31/2
Sökande: Stockholms läns landsting
Behörig företrädare: Göran Rydén
Projekt: Klinisk- och kostnadseffektivitet av internetförmedlad beteendeterapi för barn och ungdomar med Tourettes syndrom: en enkelblind randomiserad kontrollerad studie
Forskare som genomför projektet: David Mataix-Cols

Nämnden har vid sammanträdet den 10 oktober lämnat över till den vetenskaplige sekreteraren att avgöra ärendet sedan kompletteringar gjorts.

Sedan sökanden kommit in med begärda kompletteringar fattar den vetenskaplige sekreteraren följande

BESLUT

Nämnden godkänner forskningen.

På nämndens vägnar 2018-10-22

Pär Sparén
Vetenskaplig sekreterare

Beslut expedierat till behörig företrädare och ansvarig forskare

Postadress
FE 289
171 77 STOCKHOLM

Besöksadress
Tomtebodavägen 18 A
Solna

Telefon
08-524 870 00 (vx)

Fax
08-524 866 99

E-post
kansli@stockholm.epn.se

Hemsida
www.epn.se

EPN — Regionala Etikprövningsnämnden Stockholm

DECISION

Diary no.: 2018/1788-31/2

Applicant: Stockholm County Council

Authorised representative: Göran Rydén

Project: Clinical and cost-effectiveness of internet-delivered behaviour therapy for children and adolescents with Tourette's Disorder: a single-blind randomised controlled trial

Principal investigator: David Mataix-Cols

____

The review board has on the 10th of October given authority to the scientific secretary to decide on the case when complementary additions have been sent in.

The applicant has now sent in the requested complementary additions. The scientific secretary decides the following:

DECISION

The review board approves the research.

On behalf of the review board.

På nämndens vägnar 2018 -10- 22

Pär Sparén
Vetenskaplig sekreterare

Pär Sparén

Scientific secretary

The decision is dispatched to the authorised representative and the principal investigator.

Postadress FE 289 171 77 STOCKHOLM
Besöksadress Tomtebodavägen 18 A Solna
Telefon 08-524 870 00 (vx)
Fax 08-524 866 99
E-post kansli@stockholm.epn.se
Hemsida www.epn.se

BESLUT

Dnr: 2019-01670

2019-04-15

Sökande forskningshuvudman

Stockholms läns landsting

Forskare som genomför projektet

David Mataix-Cols

Projekttitel

Klinisk- och kostnadseffektivitet av internetförmedlad beteendeterapi för barn och ungdomar med Tourettes syndrom: En enkelblind randomiserad kontrollerad studie

Aktuell ändring

Ansökan om ändring, inkommen 2019-02-21

Tidigare ansökan: är godkänd av Regionala etikprövningsnämnden i Stockholm med diarienummer 2018/1788-31/2 (2019-01670).

Etikprövningsmyndigheten beslutar enligt nedan.

BESLUT

Etikprövningsmyndigheten godkänner den forskning som anges i ansökan om ändring.

.

På Etikprövningsmyndighetens vägnar

Kirsi Laakso Utvik

Ordförande

Vid beslutsfattandet har följande personer medverkat;

Ordförande
Kirsi Laakso Utvik

Ledamöter med vetenskaplig kompetens
Pär Sparén (medicinsk epidemiologi), Vetenskaplig sekreterare

---

Beslutet sänds till:
Ansvarig forskare: David Mataix-Cols

DECISION

Diary no.: 2019-01670

2019-04-15

Entity principally responsible for the research
Stockholm County Council

Principal investigator
David Mataix-Cols

Project title
Clinical and cost-effectiveness of internet-delivered behaviour therapy for children and adolescents with Tourette's Disorder: a single-blind randomised controlled trial

Current amendment
Amendment application, arrived 2019-02-21

Previous application: is approved by the Regional Ethical Review Board in Stockholm with diary number 2018/1788-31/2 (2019-01670).

The Swedish Ethical Review Authority decides according to below:

DECISION
The Swedish Ethical Review Authority approves the research that is described in the amendment application.

On behalf of the Swedish Ethical Review Authority

Kirsi Laakso Utvik
Chair

The decision making process was joined by;

Chair
Kirsi Laakso Utvik

Members with scientific expertise
Pär Sparén (medical epidemiology), Scientific secretary

The decision is sent to:
Principal investigator: David Mataix-Cols

admincontrol

2020-04836.pdf

Signers:

Name	*Method*	*Date*
RIKARD BACKELIN	BANKID	2020-10-06 14:42 GMT+2

This document package contains:

- Front page (this page)
- The original document(s)
- The electronic signatures. These are not visible in the document, but are electronically integrated.

This file is sealed with a digital signature. The seal is a guarantee for the authenticity of the document.

Document ID:
3B563F440CD549C99B1E73BA239DC6AA

THE SIGNED DOCUMENT FOLLOWS ON THE NEXT PAGE >

Dnr 2020-04836
Stockholm avdelning 2 medicin

BESLUT

Sökande forskningshuvudman
Region Stockholm

Forskare som genomför projektet
David Mataix-Cols

Projekttitel
Klinisk- och kostnadseffektivitet av internetförmedlad beteendeterapi för barn och ungdomar med Tourettes syndrom: En enkelblind randomiserad kontrollerad studie

Aktuell ändring
Ansökan om ändring inkommen 2020-09-14.

Grundansökan godkänd 2018-10-10 av Regionala Etikprövningsnämnden i Stockholm med diarienummer 2018/1788-31/2.

Etikprövningsmyndigheten beslutar enligt nedan.

BESLUT

Etikprövningsmyndigheten godkänner den forskning som anges i ansökan om ändring.

På Etikprövningsmyndighetens vägnar

Rikard Backelin
Ordförande

Beslutet har fattats efter föredragning av vetenskaplig sekreterare Britt Gustafsson.

Beslutet sänds till
Ansvarig forskare: David Mataix-Cols

This file is sealed with a digital signature.
The seal is a guarantee for the authenticity of the document.
Document ID:
3B563F440CD549C99B1E73BA239DC6AA

Diary no.: 2020-04836
Stockholm section 2 medicine

DECISION

Entity principally responsible for the research
Region Stockholm

Principal investigator
David Mataix-Cols

Project title
Clinical and cost-effectiveness of internet-delivered behaviour therapy for children and adolescents with Tourette's Disorder: a single-blind randomised controlled trial

Current amendment
Amendment application, arrived 2020-09-14

The original application is approved by the Regional Ethical Review Board in Stockholm with diary number 2018/1788-31/2.

The Swedish Ethical Review Authority decides according to below:

DECISION

The Swedish Ethical Review Authority approves the research that is described in the amendment application.

On behalf of the Swedish Ethical Review Authority

Rickard Backelin
Chair

The decision was made after a presentation by scientific secretary Britt Gustafsson.

The decision is sent to:
Principal investigator: David Mataix-Cols
